# Supplementary material for: Molecular Organisation of Tick-Borne Encephalitis Virus
Source: Viruses. 2022 Apr 11;14(4):792. doi: 10.3390/v14040792 (PMC9027435; doi:10.3390/v14040792)
Supplement: Supplementary file 1 [file viruses-14-00792-s001.zip › File_S8_PISA_analysis_of_lipid_pockets.pdf]

This website uses cookies. By continuing to browse this site, you are agreeing to the use of our site cookies. To find out more, see our Terms of Use.

OK

PISA Interface.

interface # 29 in ourmodelfortest2.pdb crystal.

Space symmetry group: P 1

interface #29/96

XML << < > >>

Interface Summary

XML

	Structure 1		Structure 2	
Selection range	[CPL]A:501		A	
class	Ligand		Protein	
symmetry operation	x,y,z		x,y,z	
symmetry ID	1_555		0_555	
Number of atoms				
interface	23	82.1%	39	1.0%
surface	26	92.9%	2439	65.1%
total	28	100.0%	3746	100.0%
Number of residues				
interface	1	100.0%	13	2.6%
surface	1	100.0%	481	97.4%
total	1	100.0%	494	100.0%
Solvent-accessible area, Å				
interface	347.5	52.3%	279.7	1.0%
total	664.6	100.0%	27709.2	100.0%
Solvation energy, kcal/mol				
isolated structure	4.2	100.0%	-444.3	100.0%
gain on complex formation	0.0	1.0%	-3.2	0.7%
average gain	-3.8	-88.5%	-1.5	0.3%
P-value	0.804		0.200	

View structure 1 | interface | structure 2

Download

structure 1 | interface | structure 2

This interface scored

0.404

in Complex Formation Significance Score (CSS).

CSS ranges from 0 to 1 as interface relevance to complex formation increases.

Achieved CSS implies that the interface plays an auxiliary role in complex formatior

Hydrogen bonds

XML

##	Structure 1	Dist. [Å]	Structure 2
1	A:CPL 501[O1P]	3.07	A:ARG 412[NH2]
2	A:CPL 501[O1P]	3.33	A:ARG 412[NH1]
3	A:CPL 501[O4P]	2.94	A:ARG 412[NH2]

No disulfide bonds found

No covalent bonds found

No salt bridges found

Interfacing residues (not a contact table)

XML Display level: Residues

Inaccessible residues

Solvent-accessible residues

HSDC Residues making **H**ydrogen/**D**isulphide bond, **S**alt bridge or **C**ovalent link

Interfacing residues

ASA Accessible Surface Area, Å² **BSA** Buried Surface Area, Å² **Δ^{i}G** Solvation energy effect, kcal/mol |||| Buried area percentage, one bar per 10%

##	Structure 1	HSDC	ASA	BSA	Δ^{i}G
1	A:CPL 501	H	664.65	347.52 |||||	-0.04

##	Structure 2	HSDC	ASA	BSA	Δ^{i}G
1	A:SER 1		4.04	0.00	0.00
2	A:ARG 2		66.31	0.00	0.00
3	A:CYS 3		0.17	0.00	0.00

4	A:THR	4	31.87	0.00	0.00
5	A:HIS	5	59.60	0.00	0.00
6	A:LEU	6	64.18	0.00	0.00
7	A:GLU	7	147.68	0.00	0.00
8	A:ASN	8	65.13	0.00	0.00
9	A:ARG	9	9.03	0.00	0.00
10	A:ASP	10	20.91	0.00	0.00
11	A:PHE	11	87.35	0.00	0.00
12	A:VAL	12	14.77	0.00	0.00
13	A:THR	13	105.26	0.00	0.00
14	A:GLY	14	18.72	0.00	0.00
15	A:THR	15	82.08	0.00	0.00
16	A:GLN	16	114.48	0.00	0.00
17	A:GLY	17	69.50	0.00	0.00
18	A:THR	18	58.84	0.00	0.00
19	A:THR	19	66.09	0.00	0.00
20	A:ARG	20	143.84	0.00	0.00
21	A:VAL	21	18.47	0.00	0.00
22	A:THR	22	35.53	0.00	0.00
23	A:LEU	23	2.32	0.00	0.00
24	A:VAL	24	4.95	0.00	0.00
25	A:LEU	25	4.35	0.00	0.00
26	A:GLU	26	35.93	0.00	0.00
27	A:LEU	27	34.77	0.00	0.00
28	A:GLY	28	58.11	0.00	0.00
29	A:GLY	29	8.75	0.00	0.00
30	A:CYS	30	5.10	0.00	0.00
31	A:VAL	31	5.69	0.00	0.00
32	A:THR	32	0.00	0.00	0.00
33	A:ILE	33	3.25	0.00	0.00
34	A:THR	34	27.47	0.00	0.00
35	A:ALA	35	20.61	0.00	0.00
36	A:GLU	36	123.01	0.00	0.00
37	A:GLY	37	35.12	0.00	0.00
38	A:LYS	38	52.27	0.00	0.00
39	A:PRO	39	14.13	0.00	0.00
40	A:SER	40	2.54	0.00	0.00
41	A:MET	41	1.17	0.00	0.00
42	A:ASP	42	0.00	0.00	0.00
43	A:VAL	43	0.50	0.00	0.00
44	A:TRP	44	10.10	0.00	0.00
45	A:LEU	45	4.92	0.00	0.00
46	A:ASP	46	64.19	0.00	0.00
47	A:ALA	47	15.55	0.00	0.00
48	A:ILE	48	0.00	0.00	0.00
49	A:TYR	49	45.43	0.00	0.00
50	A:GLN	50	4.00	0.00	0.00
51	A:GLU	51	95.58	0.00	0.00
52	A:ASN	52	104.84	0.00	0.00
53	A:PRO	53	14.62	0.00	0.00
54	A:ALA	54	59.76	0.00	0.00
55	A:LYS	55	99.24	0.00	0.00
56	A:THR	56	42.62	0.00	0.00
57	A:ARG	57	49.14	0.00	0.00
58	A:GLU	58	11.02	0.00	0.00
59	A:TYR	59	0.81	0.00	0.00
60	A:CYS	60	2.98	0.00	0.00
61	A:LEU	61	3.01	0.00	0.00
62	A:HIS	62	41.85	0.00	0.00
63	A:ALA	63	8.87	0.00	0.00
64	A:LYS	64	122.74	0.00	0.00
65	A:LEU	65	46.31	0.00	0.00
66	A:SER	66	57.05	0.00	0.00
67	A:ASP	67	93.81	0.00	0.00
68	A:THR	68	76.71	0.00	0.00
69	A:LYS	69	91.78	0.00	0.00
70	A:VAL	70	60.42	0.00	0.00
71	A:ALA	71	30.84	0.00	0.00
72	A:ALA	72	31.66	0.00	0.00

73	A:ARG 73	111.22	0.00	0.00
74	A:CYS 74	35.06	0.00	0.00
75	A:PRO 75	36.47	0.00	0.00
76	A:THR 76	99.30	0.00	0.00
77	A:MET 77	135.59	0.00	0.00
78	A:GLY 78	33.32	0.00	0.00
79	A:PRO 79	91.13	0.00	0.00
80	A:ALA 80	1.49	0.00	0.00
81	A:THR 81	89.89	0.00	0.00
82	A:LEU 82	28.42	0.00	0.00
83	A:ALA 83	77.82	0.00	0.00
84	A:GLU 84	32.66	0.00	0.00
85	A:GLU 85	52.70	0.00	0.00
86	A:HIS 86	159.33	0.00	0.00
87	A:GLN 87	113.80	0.00	0.00
88	A:GLY 88	52.60	0.00	0.00
89	A:GLY 89	22.38	0.00	0.00
90	A:THR 90	30.19	0.00	0.00
91	A:VAL 91	12.72	0.00	0.00
92	A:CYS 92	41.77	0.00	0.00
93	A:LYS 93	93.70	0.00	0.00
94	A:ARG 94	101.50	0.00	0.00
95	A:ASP 95	60.33	0.00	0.00
96	A:GLN 96	113.89	0.00	0.00
97	A:SER 97	5.11	0.00	0.00
98	A:ASP 98	97.77	0.00	0.00
99	A:ARG 99	35.12	0.00	0.00
100	A:GLY 100	5.27	0.00	0.00
101	A:TRP 101	184.07	0.00	0.00
102	A:GLY 102	79.65	0.00	0.00
103	A:ASN 103	48.25	0.00	0.00
104	A:HIS 104	192.64	0.00	0.00
105	A:CYS 105	19.81	0.00	0.00
106	A:GLY 106	52.63	0.00	0.00
107	A:LEU 107	101.90	0.00	0.00
108	A:PHE 108	139.45	0.00	0.00
109	A:GLY 109	30.32	0.00	0.00
110	A:LYS 110	114.38	0.00	0.00
111	A:GLY 111	4.23	0.00	0.00
112	A:SER 112	18.59	0.00	0.00
113	A:ILE 113	0.00	0.00	0.00
114	A:VAL 114	0.00	0.00	0.00
115	A:ALA 115	0.17	0.00	0.00
116	A:CYS 116	1.83	0.00	0.00
117	A:VAL 117	0.67	0.00	0.00
118	A:LYS 118	93.85	0.00	0.00
119	A:ALA 119	17.43	0.00	0.00
120	A:ALA 120	50.11	0.00	0.00
121	A:CYS 121	24.22	0.00	0.00
122	A:GLU 122	64.47	0.00	0.00
123	A:ALA 123	91.89	0.00	0.00
124	A:LYS 124	138.24	0.00	0.00
125	A:LYS 125	72.00	0.00	0.00
126	A:LYS 126	68.92	0.00	0.00
127	A:ALA 127	3.17	0.00	0.00
128	A:THR 128	11.80	0.00	0.00
129	A:GLY 129	0.00	0.00	0.00
130	A:HIS 130	6.13	0.00	0.00
131	A:VAL 131	46.31	0.00	0.00
132	A:TYR 132	16.12	0.00	0.00
133	A:ASP 133	54.11	0.00	0.00
134	A:ALA 134	49.87	0.00	0.00
135	A:ASN 135	111.23	0.00	0.00
136	A:LYS 136	128.53	0.00	0.00
137	A:ILE 137	0.12	0.00	0.00
138	A:VAL 138	27.17	0.00	0.00
139	A:TYR 139	2.00	0.00	0.00
140	A:THR 140	22.55	0.00	0.00
141	A:VAL 141	0.12	0.00	0.00

142	A:LYS 142	44.49	0.00	0.00
143	A:VAL 143	0.84	0.00	0.00
144	A:GLU 144	0.25	0.00	0.00
145	A:PRO 145	7.38	0.00	0.00
146	A:HIS 146	8.33	0.00	0.00
147	A:THR 147	35.21	0.00	0.00
148	A:GLY 148	16.28	0.00	0.00
149	A:ASP 149	49.00	0.00	0.00
150	A:TYR 150	83.28	0.00	0.00
151	A:VAL 151	27.89	0.00	0.00
152	A:ALA 152	54.35	0.00	0.00
153	A:ALA 153	86.37	0.00	0.00
154	A:ASN 154	145.43	0.00	0.00
155	A:GLU 155	103.24	0.00	0.00
156	A:THR 156	113.48	0.00	0.00
157	A:HIS 157	16.09	0.00	0.00
158	A:SER 158	119.10	0.00	0.00
159	A:GLY 159	21.12	0.00	0.00
160	A:ARG 160	64.09	0.00	0.00
161	A:LYS 161	83.09	0.00	0.00
162	A:THR 162	79.10	0.00	0.00
163	A:ALA 163	6.86	0.00	0.00
164	A:SER 164	77.00	0.00	0.00
165	A:PHE 165	2.37	0.00	0.00
166	A:THR 166	38.22	0.00	0.00
167	A:VAL 167	89.90	0.00	0.00
168	A:SER 168	98.65	0.00	0.00
169	A:SER 169	40.14	0.00	0.00
170	A:GLU 170	146.69	0.00	0.00
171	A:LYS 171	127.77	0.00	0.00
172	A:THR 172	43.48	0.00	0.00
173	A:ILE 173	95.72	0.00	0.00
174	A:LEU 174	21.93	0.00	0.00
175	A:THR 175	81.07	0.00	0.00
176	A:MET 176	13.11	0.00	0.00
177	A:GLY 177	61.62	0.00	0.00
178	A:GLU 178	119.63	0.00	0.00
179	A:TYR 179	30.05	0.00	0.00
180	A:GLY 180	10.72	0.00	0.00
181	A:ASP 181	34.67	0.00	0.00
182	A:VAL 182	2.62	0.00	0.00
183	A:SER 183	23.03	0.00	0.00
184	A:LEU 184	3.01	0.00	0.00
185	A:LEU 185	66.81	0.00	0.00
186	A:CYS 186	7.78	0.00	0.00
187	A:ARG 187	135.28	0.00	0.00
188	A:VAL 188	26.14	0.00	0.00
189	A:ALA 189	87.29	0.00	0.00
190	A:SER 190	19.06	0.00	0.00
191	A:GLY 191	13.78	0.00	0.00
192	A:VAL 192	22.46	0.00	0.00
193	A:ASP 193	88.10	0.00	0.00
194	A:LEU 194	24.08	0.00	0.00
195	A:ALA 195	76.02	0.00	0.00
196	A:GLN 196	80.45	0.00	0.00
197	A:THR 197	10.42	0.00	0.00
198	A:VAL 198	5.69	0.00	0.00
199	A:ILE 199	3.18	0.00	0.00
200	A:LEU 200	1.67	0.00	0.00
201	A:GLU 201	31.01	0.00	0.00
202	A:LEU 202	18.30	0.00	0.00
203	A:ASP 203	38.23	0.00	0.00
204	A:LYS 204	122.14	0.00	0.00
205	A:THR 205	98.46	0.00	0.00
206	A:VAL 206	72.55	0.00	0.00
207	A:GLU 207	144.46	0.00	0.00
208	A:HIS 208	175.54	0.00	0.00
209	A:LEU 209	62.63	0.00	0.00
210	A:PRO 210	59.42	0.00	0.00

211	A:THR 211	28.05	0.00	0.00
212	A:ALA 212	0.00	0.00	0.00
213	A:TRP 213	18.04	0.00	0.00
214	A:GLN 214	35.92	0.00	0.00
215	A:VAL 215	3.16	0.00	0.00
216	A:HIS 216	90.36	0.00	0.00
217	A:ARG 217	89.12	0.00	0.00
218	A:ASP 218	93.01	0.00	0.00
219	A:TRP 219	81.74	0.00	0.00
220	A:PHE 220	1.56	0.00	0.00
221	A:ASN 221	61.19	0.00	0.00
222	A:ASP 222	101.78	0.00	0.00
223	A:LEU 223	40.13	0.00	0.00
224	A:ALA 224	79.42	0.00	0.00
225	A:LEU 225	17.27	0.00	0.00
226	A:PRO 226	4.85	0.00	0.00
227	A:TRP 227	61.86	0.00	0.00
228	A:LYS 228	23.79	0.00	0.00
229	A:HIS 229	98.19	0.00	0.00
230	A:GLU 230	117.78	0.00	0.00
231	A:GLY 231	76.37	0.00	0.00
232	A:ALA 232	48.18	0.00	0.00
233	A:GLN 233	154.25	0.00	0.00
234	A:ASN 234	80.30	0.00	0.00
235	A:TRP 235	39.78	0.00	0.00
236	A:ASN 236	62.89	0.00	0.00
237	A:ASN 237	62.10	0.00	0.00
238	A:ALA 238	16.88	0.00	0.00
239	A:GLU 239	115.24	0.00	0.00
240	A:ARG 240	103.54	0.00	0.00
241	A:LEU 241	5.44	0.00	0.00
242	A:VAL 242	6.16	0.00	0.00
243	A:GLU 243	90.54	0.00	0.00
244	A:PHE 244	36.57	0.00	0.00
245	A:GLY 245	17.83	0.00	0.00
246	A:ALA 246	91.27	0.00	0.00
247	A:PRO 247	31.22	0.00	0.00
248	A:HIS 248	119.92	0.00	0.00
249	A:ALA 249	22.69	0.00	0.00
250	A:VAL 250	79.99	0.00	0.00
251	A:LYS 251	129.50	0.00	0.00
252	A:MET 252	12.69	0.00	0.00
253	A:ASP 253	72.00	0.00	0.00
254	A:VAL 254	34.83	0.00	0.00
255	A:TYR 255	115.21	0.00	0.00
256	A:ASN 256	65.31	0.00	0.00
257	A:LEU 257	99.59	0.00	0.00
258	A:GLY 258	22.46	0.00	0.00
259	A:ASP 259	61.51	0.00	0.00
260	A:GLN 260	41.02	0.00	0.00
261	A:THR 261	31.42	0.00	0.00
262	A:GLY 262	57.35	0.00	0.00
263	A:VAL 263	87.38	0.00	0.00
264	A:LEU 264	11.89	0.00	0.00
265	A:LEU 265	56.33	0.00	0.00
266	A:LYS 266	150.21	0.00	0.00
267	A:ALA 267	62.29	0.00	0.00
268	A:LEU 268	6.29	0.00	0.00
269	A:ALA 269	84.84	0.00	0.00
270	A:GLY 270	76.71	0.00	0.00
271	A:VAL 271	41.72	0.00	0.00
272	A:PRO 272	66.32	0.00	0.00
273	A:VAL 273	72.47	0.00	0.00
274	A:ALA 274	1.47	0.00	0.00
275	A:HIS 275	65.23	0.00	0.00
276	A:ILE 276	15.70	0.00	0.00
277	A:GLU 277	107.27	0.00	0.00
278	A:GLY 278	63.49	0.00	0.00
279	A:THR 279	63.87	0.00	0.00

280	A:LYS 280	81.07	0.00	0.00
281	A:TYR 281	25.03	0.00	0.00
282	A:HIS 282	36.08	0.00	0.00
283	A:LEU 283	1.84	0.00	0.00
284	A:LYS 284	113.23	0.00	0.00
285	A:SER 285	46.56	0.00	0.00
286	A:GLY 286	23.13	0.00	0.00
287	A:HIS 287	29.45	0.00	0.00
288	A:VAL 288	0.32	0.00	0.00
289	A:THR 289	7.50	0.00	0.00
290	A:CYS 290	2.77	0.00	0.00
291	A:GLU 291	45.78	0.00	0.00
292	A:VAL 292	1.66	0.00	0.00
293	A:GLY 293	9.33	0.00	0.00
294	A:LEU 294	4.84	0.00	0.00
295	A:GLU 295	113.26	0.00	0.00
296	A:LYS 296	115.25	0.00	0.00
297	A:LEU 297	9.99	0.00	0.00
298	A:LYS 298	125.57	0.00	0.00
299	A:MET 299	68.26	0.00	0.00
300	A:LYS 300	43.97	0.00	0.00
301	A:GLY 301	10.35	0.00	0.00
302	A:LEU 302	107.33	0.00	0.00
303	A:THR 303	132.02	0.00	0.00
304	A:TYR 304	75.83	0.00	0.00
305	A:THR 305	94.14	0.00	0.00
306	A:MET 306	101.62	0.00	0.00
307	A:CYS 307	10.44	0.00	0.00
308	A:ASP 308	74.30	0.00	0.00
309	A:LYS 309	131.91	0.00	0.00
310	A:THR 310	74.13	0.00	0.00
311	A:LYS 311	103.76	0.00	0.00
312	A:PHE 312	9.59	0.00	0.00
313	A:THR 313	75.95	0.00	0.00
314	A:TRP 314	68.99	0.00	0.00
315	A:LYS 315	124.05	0.00	0.00
316	A:ARG 316	140.85	0.00	0.00
317	A:ALA 317	49.57	0.00	0.00
318	A:PRO 318	7.24	0.00	0.00
319	A:THR 319	71.77	0.00	0.00
320	A:ASP 320	63.30	0.00	0.00
321	A:SER 321	22.85	0.00	0.00
322	A:GLY 322	74.71	0.00	0.00
323	A:HIS 323	40.31	0.00	0.00
324	A:ASP 324	40.41	0.00	0.00
325	A:THR 325	1.17	0.00	0.00
326	A:VAL 326	0.00	0.00	0.00
327	A:VAL 327	29.74	0.00	0.00
328	A:MET 328	3.88	0.00	0.00
329	A:GLU 329	28.12	0.00	0.00
330	A:VAL 330	5.58	0.00	0.00
331	A:THR 331	54.16	0.00	0.00
332	A:PHE 332	10.27	0.00	0.00
333	A:SER 333	64.87	0.00	0.00
334	A:GLY 334	37.20	0.00	0.00
335	A:THR 335	123.57	0.00	0.00
336	A:LYS 336	79.40	0.00	0.00
337	A:PRO 337	63.35	0.00	0.00
338	A:CYS 338	2.34	0.00	0.00
339	A:ARG 339	72.96	0.00	0.00
340	A:ILE 340	10.49	0.00	0.00
341	A:PRO 341	32.34	0.00	0.00
342	A:VAL 342	22.26	0.00	0.00
343	A:ARG 343	94.21	0.00	0.00
344	A:ALA 344	0.00	0.00	0.00
345	A:VAL 345	2.52	0.00	0.00
346	A:ALA 346	36.08	0.00	0.00
347	A:HIS 347	107.21	0.00	0.00
348	A:GLY 348	72.41	0.00	0.00

349	A:SER 349		53.04	0.00		0.00
350	A:PRO 350		90.60	0.00		0.00
351	A:ASP 351		137.90	0.00		0.00
352	A:VAL 352		90.33	0.00		0.00
353	A:ASN 353		85.72	0.00		0.00
354	A:VAL 354		35.62	0.00		0.00
355	A:ALA 355		17.22	0.00		0.00
356	A:MET 356		135.79	0.00		0.00
357	A:LEU 357		55.83	0.00		0.00
358	A:ILE 358		20.12	0.00		0.00
359	A:THR 359		12.89	0.00		0.00
360	A:PRO 360		50.94	0.00		0.00
361	A:ASN 361		31.16	0.00		0.00
362	A:PRO 362		14.22	0.00		0.00
363	A:THR 363		20.57	0.00		0.00
364	A:ILE 364		8.20	0.00		0.00
365	A:GLU 365		35.96	0.00		0.00
366	A:ASN 366		122.06	0.00		0.00
367	A:ASN 367		149.20	0.00		0.00
368	A:GLY 368		33.89	0.00		0.00
369	A:GLY 369		23.84	0.00		0.00
370	A:GLY 370		2.75	0.00		0.00
371	A:PHE 371		15.37	0.00		0.00
372	A:ILE 372		0.00	0.00		0.00
373	A:GLU 373		0.00	0.00		0.00
374	A:MET 374		0.00	0.00		0.00
375	A:GLN 375		51.01	0.00		0.00
376	A:LEU 376		3.69	0.00		0.00
377	A:PRO 377		45.82	0.00		0.00
378	A:PRO 378		54.33	0.00		0.00
379	A:GLY 379		24.22	0.00		0.00
380	A:ASP 380		68.35	0.00		0.00
381	A:ASN 381		2.30	0.00		0.00
382	A:ILE 382		26.77	0.00		0.00
383	A:ILE 383		0.12	0.00		0.00
384	A:TYR 384		56.65	0.00		0.00
385	A:VAL 385		0.15	0.00		0.00
386	A:GLY 386		20.09	0.00		0.00
387	A:GLU 387		109.09	0.00		0.00
388	A:LEU 388		44.39	0.00		0.00
389	A:SER 389		63.19	0.00		0.00
390	A:HIS 390		63.24	0.00		0.00
391	A:GLN 391		117.74	0.00		0.00
392	A:TRP 392		38.02	0.00		0.00
393	A:PHE 393		147.35	0.00		0.00
394	A:GLN 394		3.61	0.00		0.00
395	A:LYS 395		109.76	0.00		0.00
396	A:GLY 396		54.69	0.00		0.00
397	A:SER 397		52.78	0.00		0.00
398	A:SER 398		72.13	0.00		0.00
399	A:ILE 399		147.01	0.00		0.00
400	A:GLY 400		30.98	0.00		0.00
401	A:ARG 401		55.06	0.00		0.00
402	A:VAL 402		98.89	18.53	||	0.29
403	A:PHE 403		135.14	0.00		0.00
404	A:GLN 404		100.64	0.00		0.00
405	A:LYS 405		155.66	25.39	||	0.21
406	A:THR 406		80.60	53.04	|||||||	0.33
407	A:LYS 407		99.25	0.00		0.00
408	A:LYS 408		74.62	1.17	|	0.02
409	A:GLY 409		26.00	24.98	||||||||||	0.36
410	A:ILE 410		102.02	18.05	||	0.29
411	A:GLU 411		83.44	0.00		0.00
412	A:ARG 412	H	43.19	24.60	||||||	0.50
413	A:LEU 413		56.61	21.23	||||	0.34
414	A:THR 414		74.95	0.00		0.00
415	A:VAL 415		107.87	0.00		0.00
416	A:ILE 416		15.46	0.00		0.00
417	A:GLY 417		20.85	0.00		0.00

418	A:GLU 418	61.48	0.00		0.00
419	A:HIS 419	50.51	0.00		0.00
420	A:ALA 420	14.50	13.33	||||||||||	-0.03
421	A:TRP 421	79.24	37.12	|||||	0.53
422	A:ASP 422	28.25	0.00		0.00
423	A:PHE 423	39.74	23.04	||||||	0.04
424	A:GLY 424	32.28	0.00		0.00
425	A:SER 425	34.72	3.51	||	0.06
426	A:ALA 426	116.00	0.00		0.00
427	A:GLY 427	51.23	0.00		0.00
428	A:GLY 428	50.43	0.00		0.00
429	A:PHE 429	167.93	0.00		0.00
430	A:LEU 430	147.49	15.72	||	0.25
431	A:SER 431	15.43	0.00		0.00
432	A:SER 432	66.48	0.00		0.00
433	A:ILE 433	100.74	0.00		0.00
434	A:GLY 434	11.96	0.00		0.00
435	A:LYS 435	76.81	0.00		0.00
436	A:ALA 436	53.20	0.00		0.00
437	A:VAL 437	84.46	0.00		0.00
438	A:HIS 438	56.25	0.00		0.00
439	A:THR 439	65.43	0.00		0.00
440	A:VAL 440	98.97	0.00		0.00
441	A:LEU 441	121.82	0.00		0.00
442	A:GLY 442	32.48	0.00		0.00
443	A:GLY 443	38.24	0.00		0.00
444	A:ALA 444	62.91	0.00		0.00
445	A:PHE 445	41.55	0.00		0.00
446	A:ASN 446	100.51	0.00		0.00
447	A:SER 447	94.79	0.00		0.00
448	A:ILE 448	112.16	0.00		0.00
449	A:PHE 449	25.19	0.00		0.00
450	A:GLY 450	46.09	0.00		0.00
451	A:GLY 451	83.38	0.00		0.00
452	A:VAL 452	56.98	0.00		0.00
453	A:GLY 453	35.78	0.00		0.00
454	A:PHE 454	91.84	0.00		0.00
455	A:LEU 455	120.38	0.00		0.00
456	A:PRO 456	64.32	0.00		0.00
457	A:LYS 457	35.12	0.00		0.00
458	A:LEU 458	52.81	0.00		0.00
459	A:LEU 459	123.34	0.00		0.00
460	A:LEU 460	78.94	0.00		0.00
461	A:GLY 461	0.14	0.00		0.00
462	A:VAL 462	76.66	0.00		0.00
463	A:ALA 463	50.59	0.00		0.00
464	A:LEU 464	55.72	0.00		0.00
465	A:ALA 465	22.48	0.00		0.00
466	A:TRP 466	154.35	0.00		0.00
467	A:LEU 467	80.28	0.00		0.00
468	A:GLY 468	0.17	0.00		0.00
469	A:LEU 469	123.39	0.00		0.00
470	A:ASN 470	87.85	0.00		0.00
471	A:MET 471	58.58	0.00		0.00
472	A:ARG 472	217.43	0.00		0.00
473	A:ASN 473	59.59	0.00		0.00
474	A:PRO 474	98.38	0.00		0.00
475	A:THR 475	100.49	0.00		0.00
476	A:MET 476	93.99	0.00		0.00
477	A:SER 477	13.07	0.00		0.00
478	A:MET 478	141.64	0.00		0.00
479	A:SER 479	70.17	0.00		0.00
480	A:PHE 480	74.65	0.00		0.00
481	A:LEU 481	60.26	0.00		0.00
482	A:LEU 482	115.41	0.00		0.00
483	A:ALA 483	50.75	0.00		0.00
484	A:GLY 484	0.00	0.00		0.00
485	A:GLY 485	33.16	0.00		0.00
486	A:LEU 486	108.69	0.00		0.00

487	A:VAL 487	16.50	0.00	0.00
488	A:LEU 488	40.84	0.00	0.00
489	A:ALA 489	65.30	0.00	0.00
490	A:MET 490	81.75	0.00	0.00
491	A:THR 491	5.80	0.00	0.00
492	A:LEU 492	118.58	0.00	0.00
493	A:GLY 493	63.66	0.00	0.00
494	A:VAL 494	125.71	0.00	0.00

PDBe PISA v1.52 [20/10/2014]

PDBe Protein Data Bank in Europe

is a member of

wwPDB

EMDataBank Unified Data Resource for 3DEM

This website uses cookies. By continuing to browse this site, you are agreeing to the use of our site cookies. To find out more, see our Terms of Use.

OK

PISA Interface.

interface # 27 in ourmodelfortest2.pdb crystal.

Space symmetry group: P 1

interface #27/96

Interface Summary

XML

	Structure 1		Structure 2	
**Selection range**	[CPL]B:501		B	
**class**	Ligand		Protein	
**symmetry operation**	x,y,z		x,y,z	
**symmetry ID**	1_555		0_555	
**Number of atoms**				
**interface**	23	82.1%	43	1.1%
**surface**	27	96.4%	2443	65.2%
**total**	28	100.0%	3746	100.0%
**Number of residues**				
**interface**	1	100.0%	15	3.0%
**surface**	1	100.0%	485	98.2%
**total**	1	100.0%	494	100.0%
**Solvent-accessible area, Å**				
**interface**	351.5	51.8%	302.8	1.1%
**total**	678.8	100.0%	27658.7	100.0%
**Solvation energy, kcal/mol**				
**isolated structure**	5.5	100.0%	-446.5	100.0%
**gain on complex formation**	-3.0	-54.7%	-2.7	0.6%
**average gain**	-4.7	-85.2%	-1.6	0.4%
**P-value**	0.659		0.303	

View structure 1 | interface | structure 2

Download structure 1 | interface | structure 2

This interface scored

0.404

in Complex Formation Significance Score (CSS).

CSS ranges from 0 to 1 as interface relevance to complex formation increases.

Achieved CSS implies that the interface plays an auxiliary role in complex formatior

Hydrogen bonds

XML

##	Structure 1	Dist. [Å]	Structure 2
1	B:CPL 501[O1P]	2.92	B:ARG 412[NH2]
2	B:CPL 501[O31]	2.82	B:THR 406[OG1]

No disulfide bonds found

No covalent bonds found

No salt bridges found

Interfacing residues (not a contact table)

XML Display level: Residues

Inaccessible residues HSDC Residues making **H**ydrogen/**D**isulphide bond, **S**alt bridge or **C**ovalent link

Solvent-accessible residues Interfacing residues

ASA Accessible Surface Area, Å² **BSA** Buried Surface Area, Å² **Δ^iG** Solvation energy effect, kcal/mol |||| Buried area percentage, one bar per 10%

##	Structure 1	HSDC	ASA	BSA	Δ^iG
1	B:CPL 501	H	678.83	351.55 |||||	3.03

##	Structure 2	HSDC	ASA	BSA	Δ^iG
1	B:SER 1		13.19	0.00	0.00
2	B:ARG 2		62.08	0.00	0.00
3	B:CYS 3		0.00	0.00	0.00
4	B:THR 4		26.46	0.00	0.00

5	B:HIS	5	71.79	0.00	0.00
6	B:LEU	6	49.54	0.00	0.00
7	B:GLU	7	172.99	0.00	0.00
8	B:ASN	8	67.62	0.00	0.00
9	B:ARG	9	9.60	0.00	0.00
10	B:ASP	10	14.86	0.00	0.00
11	B:PHE	11	72.10	0.00	0.00
12	B:VAL	12	19.22	0.00	0.00
13	B:THR	13	95.23	0.00	0.00
14	B:GLY	14	18.51	0.00	0.00
15	B:THR	15	97.73	0.00	0.00
16	B:GLN	16	159.83	0.00	0.00
17	B:GLY	17	66.54	0.00	0.00
18	B:THR	18	51.44	0.00	0.00
19	B:THR	19	65.81	0.00	0.00
20	B:ARG	20	144.33	0.00	0.00
21	B:VAL	21	17.37	0.00	0.00
22	B:THR	22	26.28	0.00	0.00
23	B:LEU	23	0.17	0.00	0.00
24	B:VAL	24	3.44	0.00	0.00
25	B:LEU	25	4.19	0.00	0.00
26	B:GLU	26	41.84	0.00	0.00
27	B:LEU	27	34.43	0.00	0.00
28	B:GLY	28	54.39	0.00	0.00
29	B:GLY	29	7.41	0.00	0.00
30	B:CYS	30	9.61	0.00	0.00
31	B:VAL	31	6.52	0.00	0.00
32	B:THR	32	0.24	0.00	0.00
33	B:ILE	33	4.52	0.00	0.00
34	B:THR	34	28.86	0.00	0.00
35	B:ALA	35	18.64	0.00	0.00
36	B:GLU	36	90.86	0.00	0.00
37	B:GLY	37	49.68	0.00	0.00
38	B:LYS	38	59.39	0.00	0.00
39	B:PRO	39	11.15	0.00	0.00
40	B:SER	40	3.71	0.00	0.00
41	B:MET	41	0.50	0.00	0.00
42	B:ASP	42	0.00	0.00	0.00
43	B:VAL	43	1.50	0.00	0.00
44	B:TRP	44	11.17	0.00	0.00
45	B:LEU	45	4.80	0.00	0.00
46	B:ASP	46	63.90	0.00	0.00
47	B:ALA	47	16.50	0.00	0.00
48	B:ILE	48	0.17	0.00	0.00
49	B:TYR	49	42.21	0.00	0.00
50	B:GLN	50	6.18	0.00	0.00
51	B:GLU	51	86.82	0.00	0.00
52	B:ASN	52	106.48	0.00	0.00
53	B:PRO	53	14.50	0.00	0.00
54	B:ALA	54	51.97	0.00	0.00
55	B:LYS	55	107.55	0.00	0.00
56	B:THR	56	36.79	0.00	0.00
57	B:ARG	57	53.86	0.00	0.00
58	B:GLU	58	20.06	0.00	0.00
59	B:TYR	59	2.60	0.00	0.00
60	B:CYS	60	2.65	0.00	0.00
61	B:LEU	61	4.69	0.00	0.00
62	B:HIS	62	50.03	0.00	0.00
63	B:ALA	63	8.96	0.00	0.00
64	B:LYS	64	116.66	0.00	0.00
65	B:LEU	65	46.30	0.00	0.00
66	B:SER	66	55.95	0.00	0.00
67	B:ASP	67	76.08	0.00	0.00
68	B:THR	68	86.97	0.00	0.00
69	B:LYS	69	96.96	0.00	0.00
70	B:VAL	70	62.09	0.00	0.00
71	B:ALA	71	30.26	0.00	0.00
72	B:ALA	72	25.65	0.00	0.00
73	B:ARG	73	108.62	0.00	0.00

74	B:CYS 74	34.99	0.00	0.00
75	B:PRO 75	29.22	0.00	0.00
76	B:THR 76	90.66	0.00	0.00
77	B:MET 77	130.59	0.00	0.00
78	B:GLY 78	33.91	0.00	0.00
79	B:PRO 79	86.74	0.00	0.00
80	B:ALA 80	1.83	0.00	0.00
81	B:THR 81	95.63	0.00	0.00
82	B:LEU 82	37.13	0.00	0.00
83	B:ALA 83	72.12	0.00	0.00
84	B:GLU 84	33.22	0.00	0.00
85	B:GLU 85	59.77	0.00	0.00
86	B:HIS 86	165.39	0.00	0.00
87	B:GLN 87	111.68	0.00	0.00
88	B:GLY 88	49.83	0.00	0.00
89	B:GLY 89	23.11	0.00	0.00
90	B:THR 90	29.93	0.00	0.00
91	B:VAL 91	16.39	0.00	0.00
92	B:CYS 92	39.69	0.00	0.00
93	B:LYS 93	88.51	0.00	0.00
94	B:ARG 94	106.99	0.00	0.00
95	B:ASP 95	52.11	0.00	0.00
96	B:GLN 96	108.11	0.00	0.00
97	B:SER 97	3.35	0.00	0.00
98	B:ASP 98	97.65	0.00	0.00
99	B:ARG 99	32.90	0.00	0.00
100	B:GLY 100	4.01	0.00	0.00
101	B:TRP 101	177.25	0.00	0.00
102	B:GLY 102	76.61	0.00	0.00
103	B:ASN 103	41.34	0.00	0.00
104	B:HIS 104	170.10	0.00	0.00
105	B:CYS 105	6.20	0.00	0.00
106	B:GLY 106	57.55	0.00	0.00
107	B:LEU 107	100.01	0.00	0.00
108	B:PHE 108	138.71	0.00	0.00
109	B:GLY 109	29.80	0.00	0.00
110	B:LYS 110	114.93	0.00	0.00
111	B:GLY 111	0.82	0.00	0.00
112	B:SER 112	25.30	0.00	0.00
113	B:ILE 113	0.12	0.00	0.00
114	B:VAL 114	0.50	0.00	0.00
115	B:ALA 115	2.32	0.00	0.00
116	B:CYS 116	2.09	0.00	0.00
117	B:VAL 117	2.98	0.00	0.00
118	B:LYS 118	82.26	0.00	0.00
119	B:ALA 119	13.89	0.00	0.00
120	B:ALA 120	54.15	0.00	0.00
121	B:CYS 121	22.94	0.00	0.00
122	B:GLU 122	56.40	0.00	0.00
123	B:ALA 123	76.55	0.00	0.00
124	B:LYS 124	144.92	0.00	0.00
125	B:LYS 125	70.16	0.00	0.00
126	B:LYS 126	76.17	0.00	0.00
127	B:ALA 127	3.16	0.00	0.00
128	B:THR 128	18.86	0.00	0.00
129	B:GLY 129	0.00	0.00	0.00
130	B:HIS 130	7.88	0.00	0.00
131	B:VAL 131	45.50	0.00	0.00
132	B:TYR 132	18.90	0.00	0.00
133	B:ASP 133	66.44	0.00	0.00
134	B:ALA 134	53.14	0.00	0.00
135	B:ASN 135	113.50	0.00	0.00
136	B:LYS 136	125.12	0.00	0.00
137	B:ILE 137	2.10	0.00	0.00
138	B:VAL 138	28.40	0.00	0.00
139	B:TYR 139	2.99	0.00	0.00
140	B:THR 140	23.67	0.00	0.00
141	B:VAL 141	0.67	0.00	0.00
142	B:LYS 142	42.00	0.00	0.00

143	B:VAL 143	0.82	0.00	0.00
144	B:GLU 144	0.98	0.00	0.00
145	B:PRO 145	5.08	0.00	0.00
146	B:HIS 146	18.49	0.00	0.00
147	B:THR 147	35.71	0.00	0.00
148	B:GLY 148	19.54	0.00	0.00
149	B:ASP 149	67.55	0.00	0.00
150	B:TYR 150	68.66	0.00	0.00
151	B:VAL 151	38.49	0.00	0.00
152	B:ALA 152	51.62	0.00	0.00
153	B:ALA 153	90.69	0.00	0.00
154	B:ASN 154	138.75	0.00	0.00
155	B:GLU 155	88.06	0.00	0.00
156	B:THR 156	129.25	0.00	0.00
157	B:HIS 157	25.58	0.00	0.00
158	B:SER 158	115.86	0.00	0.00
159	B:GLY 159	17.57	0.00	0.00
160	B:ARG 160	76.96	0.00	0.00
161	B:LYS 161	75.13	0.00	0.00
162	B:THR 162	81.19	0.00	0.00
163	B:ALA 163	5.50	0.00	0.00
164	B:SER 164	72.97	0.00	0.00
165	B:PHE 165	1.29	0.00	0.00
166	B:THR 166	36.71	0.00	0.00
167	B:VAL 167	83.35	0.00	0.00
168	B:SER 168	74.77	0.00	0.00
169	B:SER 169	74.87	0.00	0.00
170	B:GLU 170	65.76	0.00	0.00
171	B:LYS 171	123.55	0.00	0.00
172	B:THR 172	40.75	0.00	0.00
173	B:ILE 173	93.30	0.00	0.00
174	B:LEU 174	17.67	0.00	0.00
175	B:THR 175	90.16	0.00	0.00
176	B:MET 176	12.22	0.00	0.00
177	B:GLY 177	58.43	0.00	0.00
178	B:GLU 178	102.49	0.00	0.00
179	B:TYR 179	29.99	0.00	0.00
180	B:GLY 180	11.49	0.00	0.00
181	B:ASP 181	36.75	0.00	0.00
182	B:VAL 182	2.77	0.00	0.00
183	B:SER 183	26.79	0.00	0.00
184	B:LEU 184	1.28	0.00	0.00
185	B:LEU 185	30.39	0.00	0.00
186	B:CYS 186	2.33	0.00	0.00
187	B:ARG 187	126.37	0.00	0.00
188	B:VAL 188	15.85	0.00	0.00
189	B:ALA 189	99.88	0.00	0.00
190	B:SER 190	34.31	0.00	0.00
191	B:GLY 191	10.49	0.00	0.00
192	B:VAL 192	21.78	0.00	0.00
193	B:ASP 193	88.32	0.00	0.00
194	B:LEU 194	36.05	0.00	0.00
195	B:ALA 195	75.11	0.00	0.00
196	B:GLN 196	82.27	0.00	0.00
197	B:THR 197	18.06	0.00	0.00
198	B:VAL 198	2.02	0.00	0.00
199	B:ILE 199	2.35	0.00	0.00
200	B:LEU 200	1.17	0.00	0.00
201	B:GLU 201	19.84	0.00	0.00
202	B:LEU 202	25.30	0.00	0.00
203	B:ASP 203	40.77	0.00	0.00
204	B:LYS 204	103.91	0.00	0.00
205	B:THR 205	110.40	0.00	0.00
206	B:VAL 206	68.15	0.00	0.00
207	B:GLU 207	133.90	0.00	0.00
208	B:HIS 208	177.89	0.00	0.00
209	B:LEU 209	71.25	0.00	0.00
210	B:PRO 210	50.04	0.00	0.00
211	B:THR 211	29.12	0.00	0.00

212	B:ALA 212	0.00	0.00	0.00
213	B:TRP 213	18.24	0.00	0.00
214	B:GLN 214	28.24	0.00	0.00
215	B:VAL 215	3.13	0.00	0.00
216	B:HIS 216	89.31	0.00	0.00
217	B:ARG 217	87.77	0.00	0.00
218	B:ASP 218	79.23	0.00	0.00
219	B:TRP 219	85.95	0.00	0.00
220	B:PHE 220	0.94	0.00	0.00
221	B:ASN 221	54.50	0.00	0.00
222	B:ASP 222	114.50	0.00	0.00
223	B:LEU 223	40.18	0.00	0.00
224	B:ALA 224	71.61	0.00	0.00
225	B:LEU 225	21.89	0.00	0.00
226	B:PRO 226	4.68	0.00	0.00
227	B:TRP 227	55.04	0.00	0.00
228	B:LYS 228	27.66	0.00	0.00
229	B:HIS 229	106.09	0.00	0.00
230	B:GLU 230	123.89	0.00	0.00
231	B:GLY 231	75.70	0.00	0.00
232	B:ALA 232	49.67	0.00	0.00
233	B:GLN 233	156.83	0.00	0.00
234	B:ASN 234	69.01	0.00	0.00
235	B:TRP 235	34.05	0.00	0.00
236	B:ASN 236	60.76	0.00	0.00
237	B:ASN 237	68.72	0.00	0.00
238	B:ALA 238	15.18	0.00	0.00
239	B:GLU 239	120.70	0.00	0.00
240	B:ARG 240	102.53	0.00	0.00
241	B:LEU 241	6.84	0.00	0.00
242	B:VAL 242	4.07	0.00	0.00
243	B:GLU 243	99.22	0.00	0.00
244	B:PHE 244	34.93	0.00	0.00
245	B:GLY 245	16.83	0.00	0.00
246	B:ALA 246	91.45	0.00	0.00
247	B:PRO 247	32.82	0.00	0.00
248	B:HIS 248	122.22	0.00	0.00
249	B:ALA 249	27.13	0.00	0.00
250	B:VAL 250	76.20	0.00	0.00
251	B:LYS 251	131.22	0.00	0.00
252	B:MET 252	19.74	0.00	0.00
253	B:ASP 253	61.71	0.00	0.00
254	B:VAL 254	43.18	0.00	0.00
255	B:TYR 255	117.45	0.00	0.00
256	B:ASN 256	69.90	0.00	0.00
257	B:LEU 257	94.79	0.00	0.00
258	B:GLY 258	24.65	0.00	0.00
259	B:ASP 259	59.91	0.00	0.00
260	B:GLN 260	37.12	0.00	0.00
261	B:THR 261	34.99	0.00	0.00
262	B:GLY 262	55.92	0.00	0.00
263	B:VAL 263	100.92	0.00	0.00
264	B:LEU 264	14.32	0.00	0.00
265	B:LEU 265	53.67	0.00	0.00
266	B:LYS 266	161.26	0.00	0.00
267	B:ALA 267	64.99	0.00	0.00
268	B:LEU 268	5.89	0.00	0.00
269	B:ALA 269	87.04	0.00	0.00
270	B:GLY 270	76.19	0.00	0.00
271	B:VAL 271	42.49	0.00	0.00
272	B:PRO 272	68.06	0.00	0.00
273	B:VAL 273	75.39	0.00	0.00
274	B:ALA 274	1.15	0.00	0.00
275	B:HIS 275	73.72	0.00	0.00
276	B:ILE 276	12.58	0.00	0.00
277	B:GLU 277	104.42	0.00	0.00
278	B:GLY 278	51.76	0.00	0.00
279	B:THR 279	72.56	0.00	0.00
280	B:LYS 280	64.90	0.00	0.00

281	B:TYR 281	22.54	0.00	0.00
282	B:HIS 282	24.77	0.00	0.00
283	B:LEU 283	2.69	0.00	0.00
284	B:LYS 284	119.43	0.00	0.00
285	B:SER 285	52.89	0.00	0.00
286	B:GLY 286	15.48	0.00	0.00
287	B:HIS 287	28.81	0.00	0.00
288	B:VAL 288	1.84	0.00	0.00
289	B:THR 289	22.84	0.00	0.00
290	B:CYS 290	2.99	0.00	0.00
291	B:GLU 291	55.77	0.00	0.00
292	B:VAL 292	0.99	0.00	0.00
293	B:GLY 293	10.72	0.00	0.00
294	B:LEU 294	12.17	0.00	0.00
295	B:GLU 295	121.57	0.00	0.00
296	B:LYS 296	140.33	0.00	0.00
297	B:LEU 297	9.60	0.00	0.00
298	B:LYS 298	123.85	0.00	0.00
299	B:MET 299	48.56	0.00	0.00
300	B:LYS 300	56.61	0.00	0.00
301	B:GLY 301	15.32	0.00	0.00
302	B:LEU 302	97.49	0.00	0.00
303	B:THR 303	129.80	0.00	0.00
304	B:TYR 304	75.60	0.00	0.00
305	B:THR 305	103.57	0.00	0.00
306	B:MET 306	123.11	0.00	0.00
307	B:CYS 307	9.02	0.00	0.00
308	B:ASP 308	66.38	0.00	0.00
309	B:LYS 309	114.69	0.00	0.00
310	B:THR 310	93.77	0.00	0.00
311	B:LYS 311	91.38	0.00	0.00
312	B:PHE 312	11.54	0.00	0.00
313	B:THR 313	69.77	0.00	0.00
314	B:TRP 314	53.43	0.00	0.00
315	B:LYS 315	136.69	0.00	0.00
316	B:ARG 316	150.20	0.00	0.00
317	B:ALA 317	50.59	0.00	0.00
318	B:PRO 318	7.48	0.00	0.00
319	B:THR 319	72.06	0.00	0.00
320	B:ASP 320	66.32	0.00	0.00
321	B:SER 321	37.47	0.00	0.00
322	B:GLY 322	63.67	0.00	0.00
323	B:HIS 323	37.82	0.00	0.00
324	B:ASP 324	58.99	0.00	0.00
325	B:THR 325	2.57	0.00	0.00
326	B:VAL 326	1.18	0.00	0.00
327	B:VAL 327	30.97	0.00	0.00
328	B:MET 328	3.81	0.00	0.00
329	B:GLU 329	23.93	0.00	0.00
330	B:VAL 330	5.94	0.00	0.00
331	B:THR 331	59.84	0.00	0.00
332	B:PHE 332	17.44	0.00	0.00
333	B:SER 333	88.83	0.00	0.00
334	B:GLY 334	17.04	0.00	0.00
335	B:THR 335	116.38	0.00	0.00
336	B:LYS 336	81.15	0.00	0.00
337	B:PRO 337	64.97	0.00	0.00
338	B:CYS 338	4.68	0.00	0.00
339	B:ARG 339	76.69	0.00	0.00
340	B:ILE 340	6.74	0.00	0.00
341	B:PRO 341	32.53	0.00	0.00
342	B:VAL 342	17.49	0.00	0.00
343	B:ARG 343	86.65	0.00	0.00
344	B:ALA 344	0.00	0.00	0.00
345	B:VAL 345	15.86	0.00	0.00
346	B:ALA 346	27.11	0.00	0.00
347	B:HIS 347	144.76	0.00	0.00
348	B:GLY 348	74.77	0.00	0.00
349	B:SER 349	59.80	0.00	0.00

<table>
<tr><td>350</td><td>B:PRO 350</td><td></td><td>115.21</td><td>0.00</td><td></td><td>0.00</td></tr>
<tr><td>351</td><td>B:ASP 351</td><td></td><td>85.07</td><td>0.00</td><td></td><td>0.00</td></tr>
<tr><td>352</td><td>B:VAL 352</td><td></td><td>90.82</td><td>0.00</td><td></td><td>0.00</td></tr>
<tr><td>353</td><td>B:ASN 353</td><td></td><td>80.15</td><td>0.00</td><td></td><td>0.00</td></tr>
<tr><td>354</td><td>B:VAL 354</td><td></td><td>52.10</td><td>0.00</td><td></td><td>0.00</td></tr>
<tr><td>355</td><td>B:ALA 355</td><td></td><td>16.65</td><td>0.00</td><td></td><td>0.00</td></tr>
<tr><td>356</td><td>B:MET 356</td><td></td><td>134.44</td><td>0.00</td><td></td><td>0.00</td></tr>
<tr><td>357</td><td>B:LEU 357</td><td></td><td>59.91</td><td>0.00</td><td></td><td>0.00</td></tr>
<tr><td>358</td><td>B:ILE 358</td><td></td><td>25.89</td><td>0.00</td><td></td><td>0.00</td></tr>
<tr><td>359</td><td>B:THR 359</td><td></td><td>5.36</td><td>0.00</td><td></td><td>0.00</td></tr>
<tr><td>360</td><td>B:PRO 360</td><td></td><td>40.55</td><td>0.00</td><td></td><td>0.00</td></tr>
<tr><td>361</td><td>B:ASN 361</td><td></td><td>38.07</td><td>0.00</td><td></td><td>0.00</td></tr>
<tr><td>362</td><td>B:PRO 362</td><td></td><td>11.88</td><td>0.00</td><td></td><td>0.00</td></tr>
<tr><td>363</td><td>B:THR 363</td><td></td><td>16.12</td><td>0.00</td><td></td><td>0.00</td></tr>
<tr><td>364</td><td>B:ILE 364</td><td></td><td>10.74</td><td>0.00</td><td></td><td>0.00</td></tr>
<tr><td>365</td><td>B:GLU 365</td><td></td><td>45.26</td><td>0.00</td><td></td><td>0.00</td></tr>
<tr><td>366</td><td>B:ASN 366</td><td></td><td>112.77</td><td>0.00</td><td></td><td>0.00</td></tr>
<tr><td>367</td><td>B:ASN 367</td><td></td><td>161.27</td><td>0.00</td><td></td><td>0.00</td></tr>
<tr><td>368</td><td>B:GLY 368</td><td></td><td>29.38</td><td>0.00</td><td></td><td>0.00</td></tr>
<tr><td>369</td><td>B:GLY 369</td><td></td><td>30.86</td><td>0.00</td><td></td><td>0.00</td></tr>
<tr><td>370</td><td>B:GLY 370</td><td></td><td>2.16</td><td>0.00</td><td></td><td>0.00</td></tr>
<tr><td>371</td><td>B:PHE 371</td><td></td><td>17.00</td><td>0.00</td><td></td><td>0.00</td></tr>
<tr><td>372</td><td>B:ILE 372</td><td></td><td>0.50</td><td>0.00</td><td></td><td>0.00</td></tr>
<tr><td>373</td><td>B:GLU 373</td><td></td><td>0.99</td><td>0.00</td><td></td><td>0.00</td></tr>
<tr><td>374</td><td>B:MET 374</td><td></td><td>1.32</td><td>0.00</td><td></td><td>0.00</td></tr>
<tr><td>375</td><td>B:GLN 375</td><td></td><td>53.70</td><td>0.00</td><td></td><td>0.00</td></tr>
<tr><td>376</td><td>B:LEU 376</td><td></td><td>4.26</td><td>0.00</td><td></td><td>0.00</td></tr>
<tr><td>377</td><td>B:PRO 377</td><td></td><td>40.59</td><td>0.00</td><td></td><td>0.00</td></tr>
<tr><td>378</td><td>B:PRO 378</td><td></td><td>50.59</td><td>0.00</td><td></td><td>0.00</td></tr>
<tr><td>379</td><td>B:GLY 379</td><td></td><td>25.23</td><td>0.00</td><td></td><td>0.00</td></tr>
<tr><td>380</td><td>B:ASP 380</td><td></td><td>71.04</td><td>0.00</td><td></td><td>0.00</td></tr>
<tr><td>381</td><td>B:ASN 381</td><td></td><td>1.62</td><td>0.00</td><td></td><td>0.00</td></tr>
<tr><td>382</td><td>B:ILE 382</td><td></td><td>56.68</td><td>0.00</td><td></td><td>0.00</td></tr>
<tr><td>383</td><td>B:ILE 383</td><td></td><td>0.50</td><td>0.00</td><td></td><td>0.00</td></tr>
<tr><td>384</td><td>B:TYR 384</td><td></td><td>63.45</td><td>0.00</td><td></td><td>0.00</td></tr>
<tr><td>385</td><td>B:VAL 385</td><td></td><td>0.00</td><td>0.00</td><td></td><td>0.00</td></tr>
<tr><td>386</td><td>B:GLY 386</td><td></td><td>11.22</td><td>0.00</td><td></td><td>0.00</td></tr>
<tr><td>387</td><td>B:GLU 387</td><td></td><td>126.03</td><td>0.00</td><td></td><td>0.00</td></tr>
<tr><td>388</td><td>B:LEU 388</td><td></td><td>49.43</td><td>0.00</td><td></td><td>0.00</td></tr>
<tr><td>389</td><td>B:SER 389</td><td></td><td>49.28</td><td>0.00</td><td></td><td>0.00</td></tr>
<tr><td>390</td><td>B:HIS 390</td><td></td><td>61.92</td><td>0.00</td><td></td><td>0.00</td></tr>
<tr><td>391</td><td>B:GLN 391</td><td></td><td>127.30</td><td>0.00</td><td></td><td>0.00</td></tr>
<tr><td>392</td><td>B:TRP 392</td><td></td><td>36.38</td><td>0.00</td><td></td><td>0.00</td></tr>
<tr><td>393</td><td>B:PHE 393</td><td></td><td>135.35</td><td>0.00</td><td></td><td>0.00</td></tr>
<tr><td>394</td><td>B:GLN 394</td><td></td><td>10.28</td><td>0.00</td><td></td><td>0.00</td></tr>
<tr><td>395</td><td>B:LYS 395</td><td></td><td>133.81</td><td>0.00</td><td></td><td>0.00</td></tr>
<tr><td>396</td><td>B:GLY 396</td><td></td><td>61.47</td><td>0.00</td><td></td><td>0.00</td></tr>
<tr><td>397</td><td>B:SER 397</td><td></td><td>55.71</td><td>0.00</td><td></td><td>0.00</td></tr>
<tr><td>398</td><td>B:SER 398</td><td></td><td>70.64</td><td>0.00</td><td></td><td>0.00</td></tr>
<tr><td>399</td><td>B:ILE 399</td><td></td><td>138.48</td><td>0.00</td><td></td><td>0.00</td></tr>
<tr><td>400</td><td>B:GLY 400</td><td></td><td>36.06</td><td>0.00</td><td></td><td>0.00</td></tr>
<tr><td>401</td><td>B:ARG 401</td><td></td><td>60.11</td><td>0.00</td><td></td><td>0.00</td></tr>
<tr><td>402</td><td>B:VAL 402</td><td></td><td>91.08</td><td>19.66</td><td>|||</td><td>0.30</td></tr>
<tr><td>403</td><td>B:PHE 403</td><td></td><td>138.16</td><td>0.00</td><td></td><td>0.00</td></tr>
<tr><td>404</td><td>B:GLN 404</td><td></td><td>84.90</td><td>0.00</td><td></td><td>0.00</td></tr>
<tr><td>405</td><td>B:LYS 405</td><td></td><td>143.70</td><td>23.46</td><td>||</td><td>-0.09</td></tr>
<tr><td>406</td><td>B:THR 406</td><td>H</td><td>73.98</td><td>56.33</td><td>||||||||</td><td>0.40</td></tr>
<tr><td>407</td><td>B:LYS 407</td><td></td><td>111.39</td><td>0.00</td><td></td><td>0.00</td></tr>
<tr><td>408</td><td>B:LYS 408</td><td></td><td>66.82</td><td>0.17</td><td>|</td><td>0.00</td></tr>
<tr><td>409</td><td>B:GLY 409</td><td></td><td>32.84</td><td>30.87</td><td>||||||||||</td><td>0.42</td></tr>
<tr><td>410</td><td>B:ILE 410</td><td></td><td>107.28</td><td>37.22</td><td>||||</td><td>0.60</td></tr>
<tr><td>411</td><td>B:GLU 411</td><td></td><td>107.81</td><td>0.00</td><td></td><td>0.00</td></tr>
<tr><td>412</td><td>B:ARG 412</td><td>H</td><td>43.34</td><td>16.62</td><td>||||</td><td>-0.04</td></tr>
<tr><td>413</td><td>B:LEU 413</td><td></td><td>55.39</td><td>10.88</td><td>||</td><td>0.17</td></tr>
<tr><td>414</td><td>B:THR 414</td><td></td><td>66.49</td><td>0.00</td><td></td><td>0.00</td></tr>
<tr><td>415</td><td>B:VAL 415</td><td></td><td>98.46</td><td>0.00</td><td></td><td>0.00</td></tr>
<tr><td>416</td><td>B:ILE 416</td><td></td><td>17.51</td><td>0.00</td><td></td><td>0.00</td></tr>
<tr><td>417</td><td>B:GLY 417</td><td></td><td>19.64</td><td>0.00</td><td></td><td>0.00</td></tr>
<tr><td>418</td><td>B:GLU 418</td><td></td><td>59.53</td><td>0.00</td><td></td><td>0.00</td></tr>
</table>

419	B:HIS 419	45.77	0.00		0.00
420	B:ALA 420	14.57	11.25	||||||||	-0.03
421	B:TRP 421	90.12	39.82	|||||	0.52
422	B:ASP 422	27.28	0.00		0.00
423	B:PHE 423	39.69	32.90	|||||||||	0.10
424	B:GLY 424	30.50	0.78	|	0.01
425	B:SER 425	29.41	8.87	||||	0.14
426	B:ALA 426	106.61	0.00		0.00
427	B:GLY 427	46.17	0.00		0.00
428	B:GLY 428	53.59	0.00		0.00
429	B:PHE 429	167.72	0.00		0.00
430	B:LEU 430	146.32	12.46	|	0.20
431	B:SER 431	14.03	1.51	||	0.02
432	B:SER 432	65.78	0.00		0.00
433	B:ILE 433	80.16	0.00		0.00
434	B:GLY 434	12.11	0.00		0.00
435	B:LYS 435	71.52	0.00		0.00
436	B:ALA 436	50.68	0.00		0.00
437	B:VAL 437	87.38	0.00		0.00
438	B:HIS 438	54.99	0.00		0.00
439	B:THR 439	64.18	0.00		0.00
440	B:VAL 440	103.08	0.00		0.00
441	B:LEU 441	84.63	0.00		0.00
442	B:GLY 442	27.86	0.00		0.00
443	B:GLY 443	39.09	0.00		0.00
444	B:ALA 444	57.91	0.00		0.00
445	B:PHE 445	40.43	0.00		0.00
446	B:ASN 446	101.94	0.00		0.00
447	B:SER 447	90.99	0.00		0.00
448	B:ILE 448	111.28	0.00		0.00
449	B:PHE 449	36.26	0.00		0.00
450	B:GLY 450	48.58	0.00		0.00
451	B:GLY 451	90.34	0.00		0.00
452	B:VAL 452	63.68	0.00		0.00
453	B:GLY 453	34.37	0.00		0.00
454	B:PHE 454	138.29	0.00		0.00
455	B:LEU 455	108.88	0.00		0.00
456	B:PRO 456	53.43	0.00		0.00
457	B:LYS 457	32.97	0.00		0.00
458	B:LEU 458	57.56	0.00		0.00
459	B:LEU 459	112.68	0.00		0.00
460	B:LEU 460	83.58	0.00		0.00
461	B:GLY 461	0.00	0.00		0.00
462	B:VAL 462	88.05	0.00		0.00
463	B:ALA 463	48.01	0.00		0.00
464	B:LEU 464	48.38	0.00		0.00
465	B:ALA 465	32.39	0.00		0.00
466	B:TRP 466	156.33	0.00		0.00
467	B:LEU 467	69.67	0.00		0.00
468	B:GLY 468	0.00	0.00		0.00
469	B:LEU 469	138.86	0.00		0.00
470	B:ASN 470	96.11	0.00		0.00
471	B:MET 471	45.09	0.00		0.00
472	B:ARG 472	213.35	0.00		0.00
473	B:ASN 473	52.02	0.00		0.00
474	B:PRO 474	109.58	0.00		0.00
475	B:THR 475	96.15	0.00		0.00
476	B:MET 476	90.31	0.00		0.00
477	B:SER 477	16.45	0.00		0.00
478	B:MET 478	127.22	0.00		0.00
479	B:SER 479	58.68	0.00		0.00
480	B:PHE 480	58.08	0.00		0.00
481	B:LEU 481	52.52	0.00		0.00
482	B:LEU 482	115.16	0.00		0.00
483	B:ALA 483	42.73	0.00		0.00
484	B:GLY 484	0.00	0.00		0.00
485	B:GLY 485	35.34	0.00		0.00
486	B:LEU 486	68.85	0.00		0.00
487	B:VAL 487	2.96	0.00		0.00

488	B:LEU 488	49.93	0.00	0.00
489	B:ALA 489	64.21	0.00	0.00
490	B:MET 490	63.51	0.00	0.00
491	B:THR 491	17.31	0.00	0.00
492	B:LEU 492	150.75	0.00	0.00
493	B:GLY 493	54.57	0.00	0.00
494	B:VAL 494	145.39	0.00	0.00

PDBe PISA v1.52 [20/10/2014]

PDBe Protein Data Bank in Europe

is a member of

PDB

EMDataBank Unified Data Resource for 3DEM

This website uses cookies. By continuing to browse this site, you are agreeing to the use of our site cookies. To find out more, see our Terms of Use.

OK

Protein Data Bank in Europe

PDBePISA

pdbe.org/pisa

PDB EMDataBank Unified Data Resource for 3DEM

Bringing Structure to Biology

PISA Interface.

interface # 32 in ourmodelfortest2.pdb crystal.

Space symmetry group: P 1

interface #32/96

Interface Summary

XML

	Structure 1		Structure 2	
**Selection range**	[CPL]C:501		C	
**class**	Ligand		Protein	
**symmetry operation**	x,y,z		x,y,z	
**symmetry ID**	1_555		0_555	
**Number of atoms**				
**interface**	24	85.7%	45	1.2%
**surface**	27	96.4%	2456	65.6%
**total**	28	100.0%	3746	100.0%
**Number of residues**				
**interface**	1	100.0%	15	3.0%
**surface**	1	100.0%	488	98.8%
**total**	1	100.0%	494	100.0%
**Solvent-accessible area, Å**				
**interface**	328.9	49.5%	281.5	1.0%
**total**	664.8	100.0%	28155.3	100.0%
**Solvation energy, kcal/mol**				
**isolated structure**	3.1	100.0%	-443.9	100.0%
**gain on complex formation**	-1.1	-36.2%	-2.0	0.5%
**average gain**	-2.8	-88.9%	-1.7	0.4%
**P-value**	0.645		0.452	

View: structure 1 | interface | structure 2

Download: structure 1 | interface | structure 2

This interface scored

0.463

in Complex Formation Significance Score (CSS).

CSS ranges from 0 to 1 as interface relevance to complex formation increases.

Achieved CSS implies that the interface plays an auxiliary role in complex formatior

Hydrogen bonds

XML

##	Structure 1	Dist. [Å]	Structure 2
1	C:CPL 501[O1P]	2.84	C:ARG 412[NH1]
2	C:CPL 501[O2P]	2.93	C:ARG 412[NH1]
3	C:CPL 501[O31]	2.84	C:THR 406[OG1]

No disulfide bonds found
No covalent bonds found
No salt bridges found

Interfacing residues (not a contact table)

XML | Display level: Residues

Inaccessible residues — HSDC Residues making **H**ydrogen/**D**isulphide bond, **S**alt bridge or **C**ovalent link

Solvent-accessible residues — Interfacing residues

ASA Accessible Surface Area, Å² **BSA** Buried Surface Area, Å² **Δ^{i}G** Solvation energy effect, kcal/mol |||| Buried area percentage, one bar per 10%

##	Structure 1	HSDC	ASA	BSA	Δ^{i}G
1	C:CPL 501	H	664.81	328.94 |||||	1.13

##	Structure 2	HSDC	ASA	BSA	Δ^{i}G
1	C:SER 1		10.29	0.00	0.00
2	C:ARG 2		74.07	0.00	0.00
3	C:CYS 3		0.00	0.00	0.00

4	C:THR	4	36.19	0.00	0.00
5	C:HIS	5	62.95	0.00	0.00
6	C:LEU	6	50.30	0.00	0.00
7	C:GLU	7	173.10	0.00	0.00
8	C:ASN	8	71.91	0.00	0.00
9	C:ARG	9	10.42	0.00	0.00
10	C:ASP	10	25.94	0.90	-0.01
11	C:PHE	11	66.35	0.00	0.00
12	C:VAL	12	20.40	0.00	0.00
13	C:THR	13	102.91	0.00	0.00
14	C:GLY	14	17.76	0.00	0.00
15	C:THR	15	92.86	0.00	0.00
16	C:GLN	16	122.11	0.00	0.00
17	C:GLY	17	76.44	0.00	0.00
18	C:THR	18	56.60	0.00	0.00
19	C:THR	19	53.54	0.00	0.00
20	C:ARG	20	170.92	0.00	0.00
21	C:VAL	21	22.41	0.00	0.00
22	C:THR	22	20.38	0.00	0.00
23	C:LEU	23	1.84	0.00	0.00
24	C:VAL	24	1.72	0.00	0.00
25	C:LEU	25	3.52	0.00	0.00
26	C:GLU	26	48.04	0.00	0.00
27	C:LEU	27	31.70	0.00	0.00
28	C:GLY	28	56.10	0.00	0.00
29	C:GLY	29	8.90	0.00	0.00
30	C:CYS	30	6.26	0.00	0.00
31	C:VAL	31	7.87	0.00	0.00
32	C:THR	32	0.17	0.00	0.00
33	C:ILE	33	5.27	0.00	0.00
34	C:THR	34	34.29	0.00	0.00
35	C:ALA	35	9.84	0.00	0.00
36	C:GLU	36	147.14	0.00	0.00
37	C:GLY	37	36.26	0.00	0.00
38	C:LYS	38	55.88	0.00	0.00
39	C:PRO	39	3.54	0.00	0.00
40	C:SER	40	1.54	0.00	0.00
41	C:MET	41	1.00	0.00	0.00
42	C:ASP	42	0.00	0.00	0.00
43	C:VAL	43	2.17	0.00	0.00
44	C:TRP	44	8.77	0.00	0.00
45	C:LEU	45	3.36	0.00	0.00
46	C:ASP	46	59.00	0.00	0.00
47	C:ALA	47	16.81	0.00	0.00
48	C:ILE	48	0.34	0.00	0.00
49	C:TYR	49	41.84	0.00	0.00
50	C:GLN	50	4.34	0.00	0.00
51	C:GLU	51	91.85	0.00	0.00
52	C:ASN	52	102.69	0.00	0.00
53	C:PRO	53	13.15	0.00	0.00
54	C:ALA	54	54.43	0.00	0.00
55	C:LYS	55	112.86	0.00	0.00
56	C:THR	56	50.77	0.00	0.00
57	C:ARG	57	57.95	0.00	0.00
58	C:GLU	58	12.29	0.00	0.00
59	C:TYR	59	1.93	0.00	0.00
60	C:CYS	60	0.17	0.00	0.00
61	C:LEU	61	0.33	0.00	0.00
62	C:HIS	62	45.88	0.00	0.00
63	C:ALA	63	7.38	0.00	0.00
64	C:LYS	64	108.37	0.00	0.00
65	C:LEU	65	43.42	0.00	0.00
66	C:SER	66	55.99	0.00	0.00
67	C:ASP	67	99.59	0.00	0.00
68	C:THR	68	84.76	0.00	0.00
69	C:LYS	69	93.25	0.00	0.00
70	C:VAL	70	66.15	0.00	0.00
71	C:ALA	71	32.41	0.00	0.00
72	C:ALA	72	30.60	0.00	0.00

73	C:ARG 73	107.47	0.00	0.00
74	C:CYS 74	30.29	0.00	0.00
75	C:PRO 75	38.73	0.00	0.00
76	C:THR 76	94.73	0.00	0.00
77	C:MET 77	125.15	0.00	0.00
78	C:GLY 78	38.73	0.00	0.00
79	C:PRO 79	87.31	0.00	0.00
80	C:ALA 80	2.66	0.00	0.00
81	C:THR 81	95.74	0.00	0.00
82	C:LEU 82	33.34	0.00	0.00
83	C:ALA 83	81.53	0.00	0.00
84	C:GLU 84	32.25	0.00	0.00
85	C:GLU 85	50.27	0.00	0.00
86	C:HIS 86	168.30	0.00	0.00
87	C:GLN 87	126.91	0.00	0.00
88	C:GLY 88	49.47	0.00	0.00
89	C:GLY 89	22.15	0.00	0.00
90	C:THR 90	33.69	0.00	0.00
91	C:VAL 91	14.56	0.00	0.00
92	C:CYS 92	42.37	0.00	0.00
93	C:LYS 93	89.07	0.00	0.00
94	C:ARG 94	100.42	0.00	0.00
95	C:ASP 95	59.65	0.00	0.00
96	C:GLN 96	110.55	0.00	0.00
97	C:SER 97	3.53	0.00	0.00
98	C:ASP 98	97.06	0.00	0.00
99	C:ARG 99	31.02	0.00	0.00
100	C:GLY 100	3.68	0.00	0.00
101	C:TRP 101	185.19	0.00	0.00
102	C:GLY 102	69.86	0.00	0.00
103	C:ASN 103	51.54	0.00	0.00
104	C:HIS 104	174.45	0.00	0.00
105	C:CYS 105	10.24	0.00	0.00
106	C:GLY 106	62.13	0.00	0.00
107	C:LEU 107	94.61	0.00	0.00
108	C:PHE 108	142.39	0.00	0.00
109	C:GLY 109	33.05	0.00	0.00
110	C:LYS 110	125.61	0.00	0.00
111	C:GLY 111	6.72	0.00	0.00
112	C:SER 112	16.29	0.00	0.00
113	C:ILE 113	0.00	0.00	0.00
114	C:VAL 114	1.01	0.00	0.00
115	C:ALA 115	1.01	0.00	0.00
116	C:CYS 116	0.66	0.00	0.00
117	C:VAL 117	3.18	0.00	0.00
118	C:LYS 118	103.54	0.00	0.00
119	C:ALA 119	16.56	0.00	0.00
120	C:ALA 120	46.71	0.00	0.00
121	C:CYS 121	29.43	0.00	0.00
122	C:GLU 122	72.17	0.00	0.00
123	C:ALA 123	85.83	0.00	0.00
124	C:LYS 124	134.45	0.00	0.00
125	C:LYS 125	54.94	0.00	0.00
126	C:LYS 126	75.11	0.00	0.00
127	C:ALA 127	0.51	0.00	0.00
128	C:THR 128	21.19	0.00	0.00
129	C:GLY 129	0.00	0.00	0.00
130	C:HIS 130	5.13	0.00	0.00
131	C:VAL 131	42.27	0.00	0.00
132	C:TYR 132	16.66	0.00	0.00
133	C:ASP 133	56.11	0.00	0.00
134	C:ALA 134	44.73	0.00	0.00
135	C:ASN 135	112.13	0.00	0.00
136	C:LYS 136	127.79	0.00	0.00
137	C:ILE 137	0.51	0.00	0.00
138	C:VAL 138	27.33	0.00	0.00
139	C:TYR 139	1.24	0.00	0.00
140	C:THR 140	22.57	0.00	0.00
141	C:VAL 141	0.82	0.00	0.00

142	C:LYS 142	45.41	0.00	0.00
143	C:VAL 143	2.76	0.00	0.00
144	C:GLU 144	1.11	0.00	0.00
145	C:PRO 145	4.63	0.00	0.00
146	C:HIS 146	7.01	0.00	0.00
147	C:THR 147	55.75	0.00	0.00
148	C:GLY 148	15.03	0.00	0.00
149	C:ASP 149	55.31	0.00	0.00
150	C:TYR 150	78.29	0.00	0.00
151	C:VAL 151	41.66	0.00	0.00
152	C:ALA 152	60.84	0.00	0.00
153	C:ALA 153	86.84	0.00	0.00
154	C:ASN 154	143.30	0.00	0.00
155	C:GLU 155	90.84	0.00	0.00
156	C:THR 156	116.46	0.00	0.00
157	C:HIS 157	25.94	0.00	0.00
158	C:SER 158	112.66	0.00	0.00
159	C:GLY 159	19.92	0.00	0.00
160	C:ARG 160	71.26	0.00	0.00
161	C:LYS 161	78.14	0.00	0.00
162	C:THR 162	78.08	0.00	0.00
163	C:ALA 163	8.16	0.00	0.00
164	C:SER 164	76.70	0.00	0.00
165	C:PHE 165	2.49	0.00	0.00
166	C:THR 166	34.91	0.00	0.00
167	C:VAL 167	75.46	0.00	0.00
168	C:SER 168	102.15	0.00	0.00
169	C:SER 169	41.07	0.00	0.00
170	C:GLU 170	127.31	0.00	0.00
171	C:LYS 171	128.13	0.00	0.00
172	C:THR 172	48.75	0.00	0.00
173	C:ILE 173	100.28	0.00	0.00
174	C:LEU 174	17.98	0.00	0.00
175	C:THR 175	85.12	0.00	0.00
176	C:MET 176	9.50	0.00	0.00
177	C:GLY 177	60.59	0.00	0.00
178	C:GLU 178	114.47	0.00	0.00
179	C:TYR 179	21.75	0.00	0.00
180	C:GLY 180	11.21	0.00	0.00
181	C:ASP 181	45.69	0.00	0.00
182	C:VAL 182	1.96	0.00	0.00
183	C:SER 183	25.39	0.00	0.00
184	C:LEU 184	1.80	0.00	0.00
185	C:LEU 185	69.53	0.00	0.00
186	C:CYS 186	8.84	0.00	0.00
187	C:ARG 187	125.57	0.00	0.00
188	C:VAL 188	23.39	0.00	0.00
189	C:ALA 189	74.81	0.00	0.00
190	C:SER 190	28.61	0.00	0.00
191	C:GLY 191	14.11	0.00	0.00
192	C:VAL 192	27.81	0.00	0.00
193	C:ASP 193	87.85	0.00	0.00
194	C:LEU 194	23.57	0.00	0.00
195	C:ALA 195	67.56	0.00	0.00
196	C:GLN 196	67.55	0.00	0.00
197	C:THR 197	17.52	0.00	0.00
198	C:VAL 198	5.86	0.00	0.00
199	C:ILE 199	2.51	0.00	0.00
200	C:LEU 200	0.51	0.00	0.00
201	C:GLU 201	27.55	0.00	0.00
202	C:LEU 202	22.83	0.00	0.00
203	C:ASP 203	36.36	0.00	0.00
204	C:LYS 204	105.02	0.00	0.00
205	C:THR 205	105.48	0.00	0.00
206	C:VAL 206	65.05	0.00	0.00
207	C:GLU 207	142.94	0.00	0.00
208	C:HIS 208	168.31	0.00	0.00
209	C:LEU 209	71.14	0.00	0.00
210	C:PRO 210	52.54	0.00	0.00

211	C:THR 211	36.45	0.00	0.00
212	C:ALA 212	0.00	0.00	0.00
213	C:TRP 213	17.36	0.00	0.00
214	C:GLN 214	24.73	0.00	0.00
215	C:VAL 215	7.45	0.00	0.00
216	C:HIS 216	87.91	0.00	0.00
217	C:ARG 217	87.65	0.00	0.00
218	C:ASP 218	81.55	0.00	0.00
219	C:TRP 219	88.51	0.00	0.00
220	C:PHE 220	3.28	0.00	0.00
221	C:ASN 221	69.35	0.00	0.00
222	C:ASP 222	113.95	0.00	0.00
223	C:LEU 223	23.90	0.00	0.00
224	C:ALA 224	68.31	0.00	0.00
225	C:LEU 225	19.86	0.00	0.00
226	C:PRO 226	3.66	0.00	0.00
227	C:TRP 227	64.79	0.00	0.00
228	C:LYS 228	23.05	0.00	0.00
229	C:HIS 229	86.16	0.00	0.00
230	C:GLU 230	131.18	0.00	0.00
231	C:GLY 231	77.82	0.00	0.00
232	C:ALA 232	48.84	0.00	0.00
233	C:GLN 233	152.38	0.00	0.00
234	C:ASN 234	76.46	0.00	0.00
235	C:TRP 235	45.28	0.00	0.00
236	C:ASN 236	61.07	0.00	0.00
237	C:ASN 237	67.82	0.00	0.00
238	C:ALA 238	15.88	0.00	0.00
239	C:GLU 239	111.31	0.00	0.00
240	C:ARG 240	113.58	0.00	0.00
241	C:LEU 241	6.06	0.00	0.00
242	C:VAL 242	4.61	0.00	0.00
243	C:GLU 243	85.09	0.00	0.00
244	C:PHE 244	23.04	0.00	0.00
245	C:GLY 245	19.82	0.00	0.00
246	C:ALA 246	92.00	0.00	0.00
247	C:PRO 247	34.98	0.00	0.00
248	C:HIS 248	117.55	0.00	0.00
249	C:ALA 249	22.31	0.00	0.00
250	C:VAL 250	68.91	0.00	0.00
251	C:LYS 251	127.07	0.00	0.00
252	C:MET 252	19.93	0.00	0.00
253	C:ASP 253	67.40	0.00	0.00
254	C:VAL 254	40.04	0.00	0.00
255	C:TYR 255	113.32	0.00	0.00
256	C:ASN 256	67.09	0.00	0.00
257	C:LEU 257	104.87	0.00	0.00
258	C:GLY 258	19.80	0.00	0.00
259	C:ASP 259	73.79	0.00	0.00
260	C:GLN 260	36.21	0.00	0.00
261	C:THR 261	34.95	0.00	0.00
262	C:GLY 262	49.82	0.00	0.00
263	C:VAL 263	89.73	0.00	0.00
264	C:LEU 264	10.04	0.00	0.00
265	C:LEU 265	57.96	0.00	0.00
266	C:LYS 266	156.71	0.00	0.00
267	C:ALA 267	59.76	0.00	0.00
268	C:LEU 268	8.91	0.00	0.00
269	C:ALA 269	88.85	0.00	0.00
270	C:GLY 270	81.91	0.00	0.00
271	C:VAL 271	48.16	0.00	0.00
272	C:PRO 272	64.23	0.00	0.00
273	C:VAL 273	74.99	0.00	0.00
274	C:ALA 274	3.12	0.00	0.00
275	C:HIS 275	69.54	0.00	0.00
276	C:ILE 276	14.78	0.00	0.00
277	C:GLU 277	98.84	0.00	0.00
278	C:GLY 278	65.88	0.00	0.00
279	C:THR 279	69.43	0.00	0.00

280	C:LYS 280	71.79	0.00	0.00
281	C:TYR 281	19.37	0.00	0.00
282	C:HIS 282	23.38	0.00	0.00
283	C:LEU 283	1.97	0.00	0.00
284	C:LYS 284	109.81	0.00	0.00
285	C:SER 285	49.18	0.00	0.00
286	C:GLY 286	16.84	0.00	0.00
287	C:HIS 287	27.22	0.00	0.00
288	C:VAL 288	1.68	0.00	0.00
289	C:THR 289	4.50	0.00	0.00
290	C:CYS 290	2.16	0.00	0.00
291	C:GLU 291	74.55	0.00	0.00
292	C:VAL 292	2.50	0.00	0.00
293	C:GLY 293	14.29	0.00	0.00
294	C:LEU 294	11.78	0.00	0.00
295	C:GLU 295	92.54	0.00	0.00
296	C:LYS 296	167.24	0.00	0.00
297	C:LEU 297	9.49	0.00	0.00
298	C:LYS 298	124.88	0.00	0.00
299	C:MET 299	52.35	0.00	0.00
300	C:LYS 300	45.49	0.00	0.00
301	C:GLY 301	15.87	0.00	0.00
302	C:LEU 302	108.95	0.00	0.00
303	C:THR 303	135.04	0.00	0.00
304	C:TYR 304	71.88	0.00	0.00
305	C:THR 305	99.62	0.00	0.00
306	C:MET 306	110.71	0.00	0.00
307	C:CYS 307	13.66	0.00	0.00
308	C:ASP 308	64.31	0.00	0.00
309	C:LYS 309	116.23	0.00	0.00
310	C:THR 310	84.41	0.00	0.00
311	C:LYS 311	93.37	0.00	0.00
312	C:PHE 312	15.13	0.00	0.00
313	C:THR 313	68.55	0.00	0.00
314	C:TRP 314	55.55	0.00	0.00
315	C:LYS 315	136.35	0.00	0.00
316	C:ARG 316	161.64	0.00	0.00
317	C:ALA 317	53.45	0.00	0.00
318	C:PRO 318	11.04	0.00	0.00
319	C:THR 319	77.83	0.00	0.00
320	C:ASP 320	81.33	0.00	0.00
321	C:SER 321	18.45	0.00	0.00
322	C:GLY 322	75.56	0.00	0.00
323	C:HIS 323	38.70	0.00	0.00
324	C:ASP 324	46.35	0.00	0.00
325	C:THR 325	3.03	0.00	0.00
326	C:VAL 326	1.01	0.00	0.00
327	C:VAL 327	25.41	0.00	0.00
328	C:MET 328	1.96	0.00	0.00
329	C:GLU 329	30.06	0.00	0.00
330	C:VAL 330	2.66	0.00	0.00
331	C:THR 331	55.87	0.00	0.00
332	C:PHE 332	7.65	0.00	0.00
333	C:SER 333	78.00	0.00	0.00
334	C:GLY 334	20.44	0.00	0.00
335	C:THR 335	119.67	0.00	0.00
336	C:LYS 336	83.73	0.00	0.00
337	C:PRO 337	57.90	0.00	0.00
338	C:CYS 338	2.51	0.00	0.00
339	C:ARG 339	67.72	0.00	0.00
340	C:ILE 340	13.20	0.00	0.00
341	C:PRO 341	28.55	0.00	0.00
342	C:VAL 342	22.03	0.00	0.00
343	C:ARG 343	118.13	0.00	0.00
344	C:ALA 344	1.05	0.00	0.00
345	C:VAL 345	15.06	0.00	0.00
346	C:ALA 346	34.04	0.00	0.00
347	C:HIS 347	118.04	0.00	0.00
348	C:GLY 348	77.71	0.00	0.00

349	C:SER 349		52.77	0.00		0.00
350	C:PRO 350		121.49	0.00		0.00
351	C:ASP 351		115.55	0.00		0.00
352	C:VAL 352		99.12	0.00		0.00
353	C:ASN 353		92.20	0.00		0.00
354	C:VAL 354		37.86	0.00		0.00
355	C:ALA 355		14.23	0.00		0.00
356	C:MET 356		125.22	0.00		0.00
357	C:LEU 357		65.02	0.00		0.00
358	C:ILE 358		21.06	0.00		0.00
359	C:THR 359		9.45	0.00		0.00
360	C:PRO 360		50.04	0.00		0.00
361	C:ASN 361		49.59	0.00		0.00
362	C:PRO 362		16.05	0.00		0.00
363	C:THR 363		28.50	0.00		0.00
364	C:ILE 364		6.38	0.00		0.00
365	C:GLU 365		47.42	0.00		0.00
366	C:ASN 366		118.66	0.00		0.00
367	C:ASN 367		139.30	0.00		0.00
368	C:GLY 368		35.01	0.00		0.00
369	C:GLY 369		36.90	0.00		0.00
370	C:GLY 370		4.35	0.00		0.00
371	C:PHE 371		13.14	0.00		0.00
372	C:ILE 372		0.49	0.00		0.00
373	C:GLU 373		0.83	0.00		0.00
374	C:MET 374		0.00	0.00		0.00
375	C:GLN 375		59.93	0.00		0.00
376	C:LEU 376		5.25	0.00		0.00
377	C:PRO 377		46.71	0.00		0.00
378	C:PRO 378		53.28	0.00		0.00
379	C:GLY 379		23.97	0.00		0.00
380	C:ASP 380		66.12	0.00		0.00
381	C:ASN 381		2.04	0.00		0.00
382	C:ILE 382		33.67	0.00		0.00
383	C:ILE 383		0.12	0.00		0.00
384	C:TYR 384		55.02	0.00		0.00
385	C:VAL 385		0.15	0.00		0.00
386	C:GLY 386		21.00	0.00		0.00
387	C:GLU 387		113.88	0.00		0.00
388	C:LEU 388		39.42	0.00		0.00
389	C:SER 389		51.25	0.00		0.00
390	C:HIS 390		59.87	0.00		0.00
391	C:GLN 391		106.66	0.00		0.00
392	C:TRP 392		38.31	0.00		0.00
393	C:PHE 393		136.80	0.00		0.00
394	C:GLN 394		12.91	0.00		0.00
395	C:LYS 395		152.01	0.00		0.00
396	C:GLY 396		65.42	0.00		0.00
397	C:SER 397		51.66	0.00		0.00
398	C:SER 398		72.56	0.00		0.00
399	C:ILE 399		141.96	0.00		0.00
400	C:GLY 400		32.78	0.00		0.00
401	C:ARG 401		54.48	0.00		0.00
402	C:VAL 402		101.34	16.10	||	0.25
403	C:PHE 403		126.89	0.00		0.00
404	C:GLN 404		112.30	0.00		0.00
405	C:LYS 405		152.17	17.47	||	0.13
406	C:THR 406	H	74.93	43.40	||||||	0.34
407	C:LYS 407		117.54	0.00		0.00
408	C:LYS 408		69.45	0.00		0.00
409	C:GLY 409		30.14	28.03	||||||||||	0.41
410	C:ILE 410		105.16	38.56	||||	0.62
411	C:GLU 411		109.48	0.00		0.00
412	C:ARG 412	H	45.73	23.32	||||||	-0.65
413	C:LEU 413		55.54	12.21	|||	0.20
414	C:THR 414		65.22	0.00		0.00
415	C:VAL 415		108.39	0.00		0.00
416	C:ILE 416		20.10	0.00		0.00
417	C:GLY 417		19.39	0.00		0.00

418	C:GLU 418	62.99	0.00		0.00
419	C:HIS 419	43.24	0.00		0.00
420	C:ALA 420	18.12	10.57	||||||	-0.09
421	C:TRP 421	80.05	40.61	||||||	0.54
422	C:ASP 422	19.42	0.00		0.00
423	C:PHE 423	37.05	28.60	||||||||	-0.02
424	C:GLY 424	35.06	1.61	|	0.03
425	C:SER 425	22.09	3.19	||	0.05
426	C:ALA 426	97.96	0.00		0.00
427	C:GLY 427	56.18	0.00		0.00
428	C:GLY 428	51.37	0.00		0.00
429	C:PHE 429	173.97	0.00		0.00
430	C:LEU 430	144.44	7.63	|	0.12
431	C:SER 431	23.85	9.33	||||	0.14
432	C:SER 432	67.42	0.00		0.00
433	C:ILE 433	97.56	0.00		0.00
434	C:GLY 434	11.30	0.00		0.00
435	C:LYS 435	79.81	0.00		0.00
436	C:ALA 436	55.76	0.00		0.00
437	C:VAL 437	75.09	0.00		0.00
438	C:HIS 438	54.99	0.00		0.00
439	C:THR 439	63.28	0.00		0.00
440	C:VAL 440	99.94	0.00		0.00
441	C:LEU 441	112.27	0.00		0.00
442	C:GLY 442	25.14	0.00		0.00
443	C:GLY 443	38.43	0.00		0.00
444	C:ALA 444	55.78	0.00		0.00
445	C:PHE 445	50.20	0.00		0.00
446	C:ASN 446	97.77	0.00		0.00
447	C:SER 447	97.70	0.00		0.00
448	C:ILE 448	125.16	0.00		0.00
449	C:PHE 449	31.61	0.00		0.00
450	C:GLY 450	44.24	0.00		0.00
451	C:GLY 451	91.86	0.00		0.00
452	C:VAL 452	60.33	0.00		0.00
453	C:GLY 453	37.75	0.00		0.00
454	C:PHE 454	151.43	0.00		0.00
455	C:LEU 455	106.10	0.00		0.00
456	C:PRO 456	58.09	0.00		0.00
457	C:LYS 457	35.80	0.00		0.00
458	C:LEU 458	83.72	0.00		0.00
459	C:LEU 459	111.54	0.00		0.00
460	C:LEU 460	85.99	0.00		0.00
461	C:GLY 461	0.24	0.00		0.00
462	C:VAL 462	74.47	0.00		0.00
463	C:ALA 463	46.95	0.00		0.00
464	C:LEU 464	51.21	0.00		0.00
465	C:ALA 465	25.77	0.00		0.00
466	C:TRP 466	163.18	0.00		0.00
467	C:LEU 467	81.75	0.00		0.00
468	C:GLY 468	1.79	0.00		0.00
469	C:LEU 469	129.47	0.00		0.00
470	C:ASN 470	93.08	0.00		0.00
471	C:MET 471	49.84	0.00		0.00
472	C:ARG 472	204.38	0.00		0.00
473	C:ASN 473	149.43	0.00		0.00
474	C:PRO 474	64.87	0.00		0.00
475	C:THR 475	119.08	0.00		0.00
476	C:MET 476	111.99	0.00		0.00
477	C:SER 477	25.89	0.00		0.00
478	C:MET 478	126.87	0.00		0.00
479	C:SER 479	63.70	0.00		0.00
480	C:PHE 480	60.04	0.00		0.00
481	C:LEU 481	68.06	0.00		0.00
482	C:LEU 482	92.54	0.00		0.00
483	C:ALA 483	46.83	0.00		0.00
484	C:GLY 484	0.16	0.00		0.00
485	C:GLY 485	34.52	0.00		0.00
486	C:LEU 486	107.71	0.00		0.00

487	C:VAL 487	9.62	0.00	0.00
488	C:LEU 488	53.04	0.00	0.00
489	C:ALA 489	57.92	0.00	0.00
490	C:MET 490	97.66	0.00	0.00
491	C:THR 491	27.49	0.00	0.00
492	C:LEU 492	140.64	0.00	0.00
493	C:GLY 493	67.87	0.00	0.00
494	C:VAL 494	137.40	0.00	0.00

PDBe PISA v1.52 [20/10/2014]

PDBe Protein Data Bank in Europe

is a member of

wwPDB

EMDataBank Unified Data Resource for 3DEM

This website uses cookies. By continuing to browse this site, you are agreeing to the use of our site cookies. To find out more, see our Terms of Use.

OK

PISA Interface.

interface # 23 in ourmodelfortest2.pdb crystal.

Space symmetry group: P 1

interface #23/96

XML << < > >>

Interface Summary

XML

	Structure 1		Structure 2	
**Selection range**	[CPL]A:500		A	
**class**	Ligand		Protein	
**symmetry operation**	x,y,z		x,y,z	
**symmetry ID**	1_555		0_555	
**Number of atoms**				
**interface**	27	96.4%	48	1.3%
**surface**	27	96.4%	2439	65.1%
**total**	28	100.0%	3746	100.0%
**Number of residues**				
**interface**	1	100.0%	18	3.6%
**surface**	1	100.0%	481	97.4%
**total**	1	100.0%	494	100.0%
**Solvent-accessible area, Å**				
**interface**	381.8	56.4%	344.2	1.2%
**total**	676.9	100.0%	27709.2	100.0%
**Solvation energy, kcal/mol**				
**isolated structure**	3.0	100.0%	-444.3	100.0%
**gain on complex formation**	-3.1	-102.1%	-4.1	0.9%
**average gain**	-3.0	-100.0%	-1.8	0.4%
**P-value**	0.499		0.157	

View structure 1 | interface | structure 2

Download

structure 1 | interface | structure 2

This interface scored

0.590

in Complex Formation Significance Score (CSS).

CSS ranges from 0 to 1 as interface relevance to complex formation increases.

Achieved CSS implies that the interface plays an essential role in complex formatior

Hydrogen bonds

XML

##	Structure 1	Dist. [Å]	Structure 2
1	A:CPL 500[O1P]	3.05	A:HIS 438[NE2]

No disulfide bonds found

No covalent bonds found

No salt bridges found

Interfacing residues (not a contact table)

XML Display level: Residues

Inaccessible residues HSDC Residues making **H**ydrogen/**D**isulphide bond, **S**alt bridge or **C**ovalent link

Solvent-accessible residues Interfacing residues

ASA Accessible Surface Area, Å² **BSA** Buried Surface Area, Å² **Δ^i^G** Solvation energy effect, kcal/mol |||| Buried area percentage, one bar per 10%

##	Structure 1	HSDC	ASA	BSA		ΔiG
1	A:CPL 500	H	676.90	381.75	|||||	3.09

##	Structure 2	HSDC	ASA	BSA	ΔiG
1	A:SER 1		4.04	0.00	0.00
2	A:ARG 2		66.31	0.00	0.00
3	A:CYS 3		0.17	0.00	0.00
4	A:THR 4		31.87	0.00	0.00
5	A:HIS 5		59.60	0.00	0.00
6	A:LEU 6		64.18	0.00	0.00

7	A:GLU	7	147.68	0.00	0.00
8	A:ASN	8	65.13	0.00	0.00
9	A:ARG	9	9.03	0.00	0.00
10	A:ASP	10	20.91	0.00	0.00
11	A:PHE	11	87.35	0.00	0.00
12	A:VAL	12	14.77	0.00	0.00
13	A:THR	13	105.26	0.00	0.00
14	A:GLY	14	18.72	0.00	0.00
15	A:THR	15	82.08	0.00	0.00
16	A:GLN	16	114.48	0.00	0.00
17	A:GLY	17	69.50	0.00	0.00
18	A:THR	18	58.84	0.00	0.00
19	A:THR	19	66.09	0.00	0.00
20	A:ARG	20	143.84	0.00	0.00
21	A:VAL	21	18.47	0.00	0.00
22	A:THR	22	35.53	0.00	0.00
23	A:LEU	23	2.32	0.00	0.00
24	A:VAL	24	4.95	0.00	0.00
25	A:LEU	25	4.35	0.00	0.00
26	A:GLU	26	35.93	0.00	0.00
27	A:LEU	27	34.77	0.00	0.00
28	A:GLY	28	58.11	0.00	0.00
29	A:GLY	29	8.75	0.00	0.00
30	A:CYS	30	5.10	0.00	0.00
31	A:VAL	31	5.69	0.00	0.00
32	A:THR	32	0.00	0.00	0.00
33	A:ILE	33	3.25	0.00	0.00
34	A:THR	34	27.47	0.00	0.00
35	A:ALA	35	20.61	0.00	0.00
36	A:GLU	36	123.01	0.00	0.00
37	A:GLY	37	35.12	0.00	0.00
38	A:LYS	38	52.27	0.00	0.00
39	A:PRO	39	14.13	0.00	0.00
40	A:SER	40	2.54	0.00	0.00
41	A:MET	41	1.17	0.00	0.00
42	A:ASP	42	0.00	0.00	0.00
43	A:VAL	43	0.50	0.00	0.00
44	A:TRP	44	10.10	0.00	0.00
45	A:LEU	45	4.92	0.00	0.00
46	A:ASP	46	64.19	0.00	0.00
47	A:ALA	47	15.55	0.00	0.00
48	A:ILE	48	0.00	0.00	0.00
49	A:TYR	49	45.43	0.00	0.00
50	A:GLN	50	4.00	0.00	0.00
51	A:GLU	51	95.58	0.00	0.00
52	A:ASN	52	104.84	0.00	0.00
53	A:PRO	53	14.62	0.00	0.00
54	A:ALA	54	59.76	0.00	0.00
55	A:LYS	55	99.24	0.00	0.00
56	A:THR	56	42.62	0.00	0.00
57	A:ARG	57	49.14	0.00	0.00
58	A:GLU	58	11.02	0.00	0.00
59	A:TYR	59	0.81	0.00	0.00
60	A:CYS	60	2.98	0.00	0.00
61	A:LEU	61	3.01	0.00	0.00
62	A:HIS	62	41.85	0.00	0.00
63	A:ALA	63	8.87	0.00	0.00
64	A:LYS	64	122.74	0.00	0.00
65	A:LEU	65	46.31	0.00	0.00
66	A:SER	66	57.05	0.00	0.00
67	A:ASP	67	93.81	0.00	0.00
68	A:THR	68	76.71	0.00	0.00
69	A:LYS	69	91.78	0.00	0.00
70	A:VAL	70	60.42	0.00	0.00
71	A:ALA	71	30.84	0.00	0.00
72	A:ALA	72	31.66	0.00	0.00
73	A:ARG	73	111.22	0.00	0.00
74	A:CYS	74	35.06	0.00	0.00
75	A:PRO	75	36.47	0.00	0.00

76	A:THR	76	99.30	0.00	0.00
77	A:MET	77	135.59	0.00	0.00
78	A:GLY	78	33.32	0.00	0.00
79	A:PRO	79	91.13	0.00	0.00
80	A:ALA	80	1.49	0.00	0.00
81	A:THR	81	89.89	0.00	0.00
82	A:LEU	82	28.42	0.00	0.00
83	A:ALA	83	77.82	0.00	0.00
84	A:GLU	84	32.66	0.00	0.00
85	A:GLU	85	52.70	0.00	0.00
86	A:HIS	86	159.33	0.00	0.00
87	A:GLN	87	113.80	0.00	0.00
88	A:GLY	88	52.60	0.00	0.00
89	A:GLY	89	22.38	0.00	0.00
90	A:THR	90	30.19	0.00	0.00
91	A:VAL	91	12.72	0.00	0.00
92	A:CYS	92	41.77	0.00	0.00
93	A:LYS	93	93.70	0.00	0.00
94	A:ARG	94	101.50	0.00	0.00
95	A:ASP	95	60.33	0.00	0.00
96	A:GLN	96	113.89	0.00	0.00
97	A:SER	97	5.11	0.00	0.00
98	A:ASP	98	97.77	0.00	0.00
99	A:ARG	99	35.12	0.00	0.00
100	A:GLY	100	5.27	0.00	0.00
101	A:TRP	101	184.07	0.00	0.00
102	A:GLY	102	79.65	0.00	0.00
103	A:ASN	103	48.25	0.00	0.00
104	A:HIS	104	192.64	0.00	0.00
105	A:CYS	105	19.81	0.00	0.00
106	A:GLY	106	52.63	0.00	0.00
107	A:LEU	107	101.90	0.00	0.00
108	A:PHE	108	139.45	0.00	0.00
109	A:GLY	109	30.32	0.00	0.00
110	A:LYS	110	114.38	0.00	0.00
111	A:GLY	111	4.23	0.00	0.00
112	A:SER	112	18.59	0.00	0.00
113	A:ILE	113	0.00	0.00	0.00
114	A:VAL	114	0.00	0.00	0.00
115	A:ALA	115	0.17	0.00	0.00
116	A:CYS	116	1.83	0.00	0.00
117	A:VAL	117	0.67	0.00	0.00
118	A:LYS	118	93.85	0.00	0.00
119	A:ALA	119	17.43	0.00	0.00
120	A:ALA	120	50.11	0.00	0.00
121	A:CYS	121	24.22	0.00	0.00
122	A:GLU	122	64.47	0.00	0.00
123	A:ALA	123	91.89	0.00	0.00
124	A:LYS	124	138.24	0.00	0.00
125	A:LYS	125	72.00	0.00	0.00
126	A:LYS	126	68.92	0.00	0.00
127	A:ALA	127	3.17	0.00	0.00
128	A:THR	128	11.80	0.00	0.00
129	A:GLY	129	0.00	0.00	0.00
130	A:HIS	130	6.13	0.00	0.00
131	A:VAL	131	46.31	0.00	0.00
132	A:TYR	132	16.12	0.00	0.00
133	A:ASP	133	54.11	0.00	0.00
134	A:ALA	134	49.87	0.00	0.00
135	A:ASN	135	111.23	0.00	0.00
136	A:LYS	136	128.53	0.00	0.00
137	A:ILE	137	0.12	0.00	0.00
138	A:VAL	138	27.17	0.00	0.00
139	A:TYR	139	2.00	0.00	0.00
140	A:THR	140	22.55	0.00	0.00
141	A:VAL	141	0.12	0.00	0.00
142	A:LYS	142	44.49	0.00	0.00
143	A:VAL	143	0.84	0.00	0.00
144	A:GLU	144	0.25	0.00	0.00

145	A:PRO 145	7.38	0.00	0.00
146	A:HIS 146	8.33	0.00	0.00
147	A:THR 147	35.21	0.00	0.00
148	A:GLY 148	16.28	0.00	0.00
149	A:ASP 149	49.00	0.00	0.00
150	A:TYR 150	83.28	0.00	0.00
151	A:VAL 151	27.89	0.00	0.00
152	A:ALA 152	54.35	0.00	0.00
153	A:ALA 153	86.37	0.00	0.00
154	A:ASN 154	145.43	0.00	0.00
155	A:GLU 155	103.24	0.00	0.00
156	A:THR 156	113.48	0.00	0.00
157	A:HIS 157	16.09	0.00	0.00
158	A:SER 158	119.10	0.00	0.00
159	A:GLY 159	21.12	0.00	0.00
160	A:ARG 160	64.09	0.00	0.00
161	A:LYS 161	83.09	0.00	0.00
162	A:THR 162	79.10	0.00	0.00
163	A:ALA 163	6.86	0.00	0.00
164	A:SER 164	77.00	0.00	0.00
165	A:PHE 165	2.37	0.00	0.00
166	A:THR 166	38.22	0.00	0.00
167	A:VAL 167	89.90	0.00	0.00
168	A:SER 168	98.65	0.00	0.00
169	A:SER 169	40.14	0.00	0.00
170	A:GLU 170	146.69	0.00	0.00
171	A:LYS 171	127.77	0.00	0.00
172	A:THR 172	43.48	0.00	0.00
173	A:ILE 173	95.72	0.00	0.00
174	A:LEU 174	21.93	0.00	0.00
175	A:THR 175	81.07	0.00	0.00
176	A:MET 176	13.11	0.00	0.00
177	A:GLY 177	61.62	0.00	0.00
178	A:GLU 178	119.63	0.00	0.00
179	A:TYR 179	30.05	0.00	0.00
180	A:GLY 180	10.72	0.00	0.00
181	A:ASP 181	34.67	0.00	0.00
182	A:VAL 182	2.62	0.00	0.00
183	A:SER 183	23.03	0.00	0.00
184	A:LEU 184	3.01	0.00	0.00
185	A:LEU 185	66.81	0.00	0.00
186	A:CYS 186	7.78	0.00	0.00
187	A:ARG 187	135.28	0.00	0.00
188	A:VAL 188	26.14	0.00	0.00
189	A:ALA 189	87.29	0.00	0.00
190	A:SER 190	19.06	0.00	0.00
191	A:GLY 191	13.78	0.00	0.00
192	A:VAL 192	22.46	0.00	0.00
193	A:ASP 193	88.10	0.00	0.00
194	A:LEU 194	24.08	0.00	0.00
195	A:ALA 195	76.02	0.00	0.00
196	A:GLN 196	80.45	0.00	0.00
197	A:THR 197	10.42	0.00	0.00
198	A:VAL 198	5.69	0.00	0.00
199	A:ILE 199	3.18	0.00	0.00
200	A:LEU 200	1.67	0.00	0.00
201	A:GLU 201	31.01	0.00	0.00
202	A:LEU 202	18.30	0.00	0.00
203	A:ASP 203	38.23	0.00	0.00
204	A:LYS 204	122.14	0.00	0.00
205	A:THR 205	98.46	0.00	0.00
206	A:VAL 206	72.55	0.00	0.00
207	A:GLU 207	144.46	0.00	0.00
208	A:HIS 208	175.54	0.00	0.00
209	A:LEU 209	62.63	0.00	0.00
210	A:PRO 210	59.42	0.00	0.00
211	A:THR 211	28.05	0.00	0.00
212	A:ALA 212	0.00	0.00	0.00
213	A:TRP 213	18.04	0.00	0.00

214	A:GLN 214	35.92	0.00	0.00
215	A:VAL 215	3.16	0.00	0.00
216	A:HIS 216	90.36	0.00	0.00
217	A:ARG 217	89.12	0.00	0.00
218	A:ASP 218	93.01	0.00	0.00
219	A:TRP 219	81.74	0.00	0.00
220	A:PHE 220	1.56	0.00	0.00
221	A:ASN 221	61.19	0.00	0.00
222	A:ASP 222	101.78	0.00	0.00
223	A:LEU 223	40.13	0.00	0.00
224	A:ALA 224	79.42	0.00	0.00
225	A:LEU 225	17.27	0.00	0.00
226	A:PRO 226	4.85	0.00	0.00
227	A:TRP 227	61.86	0.00	0.00
228	A:LYS 228	23.79	0.00	0.00
229	A:HIS 229	98.19	0.00	0.00
230	A:GLU 230	117.78	0.00	0.00
231	A:GLY 231	76.37	0.00	0.00
232	A:ALA 232	48.18	0.00	0.00
233	A:GLN 233	154.25	0.00	0.00
234	A:ASN 234	80.30	0.00	0.00
235	A:TRP 235	39.78	0.00	0.00
236	A:ASN 236	62.89	0.00	0.00
237	A:ASN 237	62.10	0.00	0.00
238	A:ALA 238	16.88	0.00	0.00
239	A:GLU 239	115.24	0.00	0.00
240	A:ARG 240	103.54	0.00	0.00
241	A:LEU 241	5.44	0.00	0.00
242	A:VAL 242	6.16	0.00	0.00
243	A:GLU 243	90.54	0.00	0.00
244	A:PHE 244	36.57	0.00	0.00
245	A:GLY 245	17.83	0.00	0.00
246	A:ALA 246	91.27	0.00	0.00
247	A:PRO 247	31.22	0.00	0.00
248	A:HIS 248	119.92	0.00	0.00
249	A:ALA 249	22.69	0.00	0.00
250	A:VAL 250	79.99	0.00	0.00
251	A:LYS 251	129.50	0.00	0.00
252	A:MET 252	12.69	0.00	0.00
253	A:ASP 253	72.00	0.00	0.00
254	A:VAL 254	34.83	0.00	0.00
255	A:TYR 255	115.21	0.00	0.00
256	A:ASN 256	65.31	0.00	0.00
257	A:LEU 257	99.59	0.00	0.00
258	A:GLY 258	22.46	0.00	0.00
259	A:ASP 259	61.51	0.00	0.00
260	A:GLN 260	41.02	0.00	0.00
261	A:THR 261	31.42	0.00	0.00
262	A:GLY 262	57.35	0.00	0.00
263	A:VAL 263	87.38	0.00	0.00
264	A:LEU 264	11.89	0.00	0.00
265	A:LEU 265	56.33	0.00	0.00
266	A:LYS 266	150.21	0.00	0.00
267	A:ALA 267	62.29	0.00	0.00
268	A:LEU 268	6.29	0.00	0.00
269	A:ALA 269	84.84	0.00	0.00
270	A:GLY 270	76.71	0.00	0.00
271	A:VAL 271	41.72	0.00	0.00
272	A:PRO 272	66.32	0.00	0.00
273	A:VAL 273	72.47	0.00	0.00
274	A:ALA 274	1.47	0.00	0.00
275	A:HIS 275	65.23	0.00	0.00
276	A:ILE 276	15.70	0.00	0.00
277	A:GLU 277	107.27	0.00	0.00
278	A:GLY 278	63.49	0.00	0.00
279	A:THR 279	63.87	0.00	0.00
280	A:LYS 280	81.07	0.00	0.00
281	A:TYR 281	25.03	0.00	0.00
282	A:HIS 282	36.08	0.00	0.00

283	A:LEU 283	1.84	0.00	0.00
284	A:LYS 284	113.23	0.00	0.00
285	A:SER 285	46.56	0.00	0.00
286	A:GLY 286	23.13	0.00	0.00
287	A:HIS 287	29.45	0.00	0.00
288	A:VAL 288	0.32	0.00	0.00
289	A:THR 289	7.50	0.00	0.00
290	A:CYS 290	2.77	0.00	0.00
291	A:GLU 291	45.78	0.00	0.00
292	A:VAL 292	1.66	0.00	0.00
293	A:GLY 293	9.33	0.00	0.00
294	A:LEU 294	4.84	0.00	0.00
295	A:GLU 295	113.26	0.00	0.00
296	A:LYS 296	115.25	0.00	0.00
297	A:LEU 297	9.99	0.00	0.00
298	A:LYS 298	125.57	0.00	0.00
299	A:MET 299	68.26	0.00	0.00
300	A:LYS 300	43.97	0.00	0.00
301	A:GLY 301	10.35	0.00	0.00
302	A:LEU 302	107.33	0.00	0.00
303	A:THR 303	132.02	0.00	0.00
304	A:TYR 304	75.83	0.00	0.00
305	A:THR 305	94.14	0.00	0.00
306	A:MET 306	101.62	0.00	0.00
307	A:CYS 307	10.44	0.00	0.00
308	A:ASP 308	74.30	0.00	0.00
309	A:LYS 309	131.91	0.00	0.00
310	A:THR 310	74.13	0.00	0.00
311	A:LYS 311	103.76	0.00	0.00
312	A:PHE 312	9.59	0.00	0.00
313	A:THR 313	75.95	0.00	0.00
314	A:TRP 314	68.99	0.00	0.00
315	A:LYS 315	124.05	0.00	0.00
316	A:ARG 316	140.85	0.00	0.00
317	A:ALA 317	49.57	0.00	0.00
318	A:PRO 318	7.24	0.00	0.00
319	A:THR 319	71.77	0.00	0.00
320	A:ASP 320	63.30	0.00	0.00
321	A:SER 321	22.85	0.00	0.00
322	A:GLY 322	74.71	0.00	0.00
323	A:HIS 323	40.31	0.00	0.00
324	A:ASP 324	40.41	0.00	0.00
325	A:THR 325	1.17	0.00	0.00
326	A:VAL 326	0.00	0.00	0.00
327	A:VAL 327	29.74	0.00	0.00
328	A:MET 328	3.88	0.00	0.00
329	A:GLU 329	28.12	0.00	0.00
330	A:VAL 330	5.58	0.00	0.00
331	A:THR 331	54.16	0.00	0.00
332	A:PHE 332	10.27	0.00	0.00
333	A:SER 333	64.87	0.00	0.00
334	A:GLY 334	37.20	0.00	0.00
335	A:THR 335	123.57	0.00	0.00
336	A:LYS 336	79.40	0.00	0.00
337	A:PRO 337	63.35	0.00	0.00
338	A:CYS 338	2.34	0.00	0.00
339	A:ARG 339	72.96	0.00	0.00
340	A:ILE 340	10.49	0.00	0.00
341	A:PRO 341	32.34	0.00	0.00
342	A:VAL 342	22.26	0.00	0.00
343	A:ARG 343	94.21	0.00	0.00
344	A:ALA 344	0.00	0.00	0.00
345	A:VAL 345	2.52	0.00	0.00
346	A:ALA 346	36.08	0.00	0.00
347	A:HIS 347	107.21	0.00	0.00
348	A:GLY 348	72.41	0.00	0.00
349	A:SER 349	53.04	0.00	0.00
350	A:PRO 350	90.60	0.00	0.00
351	A:ASP 351	137.90	0.00	0.00

<table>
<tr><td>352</td><td>A:VAL 352</td><td>90.33</td><td>0.00</td><td></td><td>0.00</td></tr>
<tr><td>353</td><td>A:ASN 353</td><td>85.72</td><td>0.00</td><td></td><td>0.00</td></tr>
<tr><td>354</td><td>A:VAL 354</td><td>35.62</td><td>0.00</td><td></td><td>0.00</td></tr>
<tr><td>355</td><td>A:ALA 355</td><td>17.22</td><td>0.00</td><td></td><td>0.00</td></tr>
<tr><td>356</td><td>A:MET 356</td><td>135.79</td><td>0.00</td><td></td><td>0.00</td></tr>
<tr><td>357</td><td>A:LEU 357</td><td>55.83</td><td>0.00</td><td></td><td>0.00</td></tr>
<tr><td>358</td><td>A:ILE 358</td><td>20.12</td><td>0.00</td><td></td><td>0.00</td></tr>
<tr><td>359</td><td>A:THR 359</td><td>12.89</td><td>0.00</td><td></td><td>0.00</td></tr>
<tr><td>360</td><td>A:PRO 360</td><td>50.94</td><td>0.00</td><td></td><td>0.00</td></tr>
<tr><td>361</td><td>A:ASN 361</td><td>31.16</td><td>0.00</td><td></td><td>0.00</td></tr>
<tr><td>362</td><td>A:PRO 362</td><td>14.22</td><td>0.00</td><td></td><td>0.00</td></tr>
<tr><td>363</td><td>A:THR 363</td><td>20.57</td><td>0.00</td><td></td><td>0.00</td></tr>
<tr><td>364</td><td>A:ILE 364</td><td>8.20</td><td>0.00</td><td></td><td>0.00</td></tr>
<tr><td>365</td><td>A:GLU 365</td><td>35.96</td><td>0.00</td><td></td><td>0.00</td></tr>
<tr><td>366</td><td>A:ASN 366</td><td>122.06</td><td>0.00</td><td></td><td>0.00</td></tr>
<tr><td>367</td><td>A:ASN 367</td><td>149.20</td><td>0.00</td><td></td><td>0.00</td></tr>
<tr><td>368</td><td>A:GLY 368</td><td>33.89</td><td>0.00</td><td></td><td>0.00</td></tr>
<tr><td>369</td><td>A:GLY 369</td><td>23.84</td><td>0.00</td><td></td><td>0.00</td></tr>
<tr><td>370</td><td>A:GLY 370</td><td>2.75</td><td>0.00</td><td></td><td>0.00</td></tr>
<tr><td>371</td><td>A:PHE 371</td><td>15.37</td><td>0.00</td><td></td><td>0.00</td></tr>
<tr><td>372</td><td>A:ILE 372</td><td>0.00</td><td>0.00</td><td></td><td>0.00</td></tr>
<tr><td>373</td><td>A:GLU 373</td><td>0.00</td><td>0.00</td><td></td><td>0.00</td></tr>
<tr><td>374</td><td>A:MET 374</td><td>0.00</td><td>0.00</td><td></td><td>0.00</td></tr>
<tr><td>375</td><td>A:GLN 375</td><td>51.01</td><td>0.00</td><td></td><td>0.00</td></tr>
<tr><td>376</td><td>A:LEU 376</td><td>3.69</td><td>0.00</td><td></td><td>0.00</td></tr>
<tr><td>377</td><td>A:PRO 377</td><td>45.82</td><td>0.00</td><td></td><td>0.00</td></tr>
<tr><td>378</td><td>A:PRO 378</td><td>54.33</td><td>0.00</td><td></td><td>0.00</td></tr>
<tr><td>379</td><td>A:GLY 379</td><td>24.22</td><td>0.00</td><td></td><td>0.00</td></tr>
<tr><td>380</td><td>A:ASP 380</td><td>68.35</td><td>0.00</td><td></td><td>0.00</td></tr>
<tr><td>381</td><td>A:ASN 381</td><td>2.30</td><td>0.00</td><td></td><td>0.00</td></tr>
<tr><td>382</td><td>A:ILE 382</td><td>26.77</td><td>0.00</td><td></td><td>0.00</td></tr>
<tr><td>383</td><td>A:ILE 383</td><td>0.12</td><td>0.00</td><td></td><td>0.00</td></tr>
<tr><td>384</td><td>A:TYR 384</td><td>56.65</td><td>0.00</td><td></td><td>0.00</td></tr>
<tr><td>385</td><td>A:VAL 385</td><td>0.15</td><td>0.00</td><td></td><td>0.00</td></tr>
<tr><td>386</td><td>A:GLY 386</td><td>20.09</td><td>0.00</td><td></td><td>0.00</td></tr>
<tr><td>387</td><td>A:GLU 387</td><td>109.09</td><td>0.00</td><td></td><td>0.00</td></tr>
<tr><td>388</td><td>A:LEU 388</td><td>44.39</td><td>0.00</td><td></td><td>0.00</td></tr>
<tr><td>389</td><td>A:SER 389</td><td>63.19</td><td>0.00</td><td></td><td>0.00</td></tr>
<tr><td>390</td><td>A:HIS 390</td><td>63.24</td><td>0.00</td><td></td><td>0.00</td></tr>
<tr><td>391</td><td>A:GLN 391</td><td>117.74</td><td>0.00</td><td></td><td>0.00</td></tr>
<tr><td>392</td><td>A:TRP 392</td><td>38.02</td><td>0.00</td><td></td><td>0.00</td></tr>
<tr><td>393</td><td>A:PHE 393</td><td>147.35</td><td>0.00</td><td></td><td>0.00</td></tr>
<tr><td>394</td><td>A:GLN 394</td><td>3.61</td><td>0.00</td><td></td><td>0.00</td></tr>
<tr><td>395</td><td>A:LYS 395</td><td>109.76</td><td>0.00</td><td></td><td>0.00</td></tr>
<tr><td>396</td><td>A:GLY 396</td><td>54.69</td><td>0.00</td><td></td><td>0.00</td></tr>
<tr><td>397</td><td>A:SER 397</td><td>52.78</td><td>0.00</td><td></td><td>0.00</td></tr>
<tr><td>398</td><td>A:SER 398</td><td>72.13</td><td>0.00</td><td></td><td>0.00</td></tr>
<tr><td>399</td><td>A:ILE 399</td><td>147.01</td><td>0.00</td><td></td><td>0.00</td></tr>
<tr><td>400</td><td>A:GLY 400</td><td>30.98</td><td>0.00</td><td></td><td>0.00</td></tr>
<tr><td>401</td><td>A:ARG 401</td><td>55.06</td><td>0.00</td><td></td><td>0.00</td></tr>
<tr><td>402</td><td>A:VAL 402</td><td>98.89</td><td>0.00</td><td></td><td>0.00</td></tr>
<tr><td>403</td><td>A:PHE 403</td><td>135.14</td><td>0.00</td><td></td><td>0.00</td></tr>
<tr><td>404</td><td>A:GLN 404</td><td>100.64</td><td>0.00</td><td></td><td>0.00</td></tr>
<tr><td>405</td><td>A:LYS 405</td><td>155.66</td><td>0.00</td><td></td><td>0.00</td></tr>
<tr><td>406</td><td>A:THR 406</td><td>80.60</td><td>0.00</td><td></td><td>0.00</td></tr>
<tr><td>407</td><td>A:LYS 407</td><td>99.25</td><td>0.00</td><td></td><td>0.00</td></tr>
<tr><td>408</td><td>A:LYS 408</td><td>74.62</td><td>0.00</td><td></td><td>0.00</td></tr>
<tr><td>409</td><td>A:GLY 409</td><td>26.00</td><td>0.00</td><td></td><td>0.00</td></tr>
<tr><td>410</td><td>A:ILE 410</td><td>102.02</td><td>29.75</td><td>|||</td><td>0.47</td></tr>
<tr><td>411</td><td>A:GLU 411</td><td>83.44</td><td>0.00</td><td></td><td>0.00</td></tr>
<tr><td>412</td><td>A:ARG 412</td><td>43.19</td><td>0.00</td><td></td><td>0.00</td></tr>
<tr><td>413</td><td>A:LEU 413</td><td>56.61</td><td>34.54</td><td>|||||||</td><td>0.44</td></tr>
<tr><td>414</td><td>A:THR 414</td><td>74.95</td><td>33.54</td><td>|||||</td><td>-0.14</td></tr>
<tr><td>415</td><td>A:VAL 415</td><td>107.87</td><td>12.19</td><td>||</td><td>-0.13</td></tr>
<tr><td>416</td><td>A:ILE 416</td><td>15.46</td><td>4.46</td><td>|||</td><td>0.07</td></tr>
<tr><td>417</td><td>A:GLY 417</td><td>20.85</td><td>20.51</td><td>||||||||||</td><td>0.28</td></tr>
<tr><td>418</td><td>A:GLU 418</td><td>61.48</td><td>4.42</td><td>|</td><td>-0.05</td></tr>
<tr><td>419</td><td>A:HIS 419</td><td>50.51</td><td>0.00</td><td></td><td>0.00</td></tr>
<tr><td>420</td><td>A:ALA 420</td><td>14.50</td><td>0.00</td><td></td><td>0.00</td></tr>
</table>

<table>
<tr><td>421</td><td>A:TRP 421</td><td></td><td>79.24</td><td>17.69</td><td>|||</td><td>0.28</td></tr>
<tr><td>422</td><td>A:ASP 422</td><td></td><td>28.25</td><td>0.00</td><td></td><td>0.00</td></tr>
<tr><td>423</td><td>A:PHE 423</td><td></td><td>39.74</td><td>0.00</td><td></td><td>0.00</td></tr>
<tr><td>424</td><td>A:GLY 424</td><td></td><td>32.28</td><td>0.00</td><td></td><td>0.00</td></tr>
<tr><td>425</td><td>A:SER 425</td><td></td><td>34.72</td><td>0.00</td><td></td><td>0.00</td></tr>
<tr><td>426</td><td>A:ALA 426</td><td></td><td>116.00</td><td>0.00</td><td></td><td>0.00</td></tr>
<tr><td>427</td><td>A:GLY 427</td><td></td><td>51.23</td><td>0.00</td><td></td><td>0.00</td></tr>
<tr><td>428</td><td>A:GLY 428</td><td></td><td>50.43</td><td>0.00</td><td></td><td>0.00</td></tr>
<tr><td>429</td><td>A:PHE 429</td><td></td><td>167.93</td><td>0.00</td><td></td><td>0.00</td></tr>
<tr><td>430</td><td>A:LEU 430</td><td></td><td>147.49</td><td>0.00</td><td></td><td>0.00</td></tr>
<tr><td>431</td><td>A:SER 431</td><td></td><td>15.43</td><td>0.00</td><td></td><td>0.00</td></tr>
<tr><td>432</td><td>A:SER 432</td><td></td><td>66.48</td><td>0.00</td><td></td><td>0.00</td></tr>
<tr><td>433</td><td>A:ILE 433</td><td></td><td>100.74</td><td>0.00</td><td></td><td>0.00</td></tr>
<tr><td>434</td><td>A:GLY 434</td><td></td><td>11.96</td><td>3.70</td><td>||||</td><td>0.04</td></tr>
<tr><td>435</td><td>A:LYS 435</td><td></td><td>76.81</td><td>0.00</td><td></td><td>0.00</td></tr>
<tr><td>436</td><td>A:ALA 436</td><td></td><td>53.20</td><td>0.00</td><td></td><td>0.00</td></tr>
<tr><td>437</td><td>A:VAL 437</td><td></td><td>84.46</td><td>16.91</td><td>|||</td><td>0.27</td></tr>
<tr><td>438</td><td>A:HIS 438</td><td>H</td><td>56.25</td><td>54.42</td><td>||||||||||</td><td>0.76</td></tr>
<tr><td>439</td><td>A:THR 439</td><td></td><td>65.43</td><td>0.00</td><td></td><td>0.00</td></tr>
<tr><td>440</td><td>A:VAL 440</td><td></td><td>98.97</td><td>0.00</td><td></td><td>0.00</td></tr>
<tr><td>441</td><td>A:LEU 441</td><td></td><td>121.82</td><td>20.26</td><td>||</td><td>0.32</td></tr>
<tr><td>442</td><td>A:GLY 442</td><td></td><td>32.48</td><td>17.83</td><td>||||||</td><td>0.24</td></tr>
<tr><td>443</td><td>A:GLY 443</td><td></td><td>38.24</td><td>0.00</td><td></td><td>0.00</td></tr>
<tr><td>444</td><td>A:ALA 444</td><td></td><td>62.91</td><td>0.00</td><td></td><td>0.00</td></tr>
<tr><td>445</td><td>A:PHE 445</td><td></td><td>41.55</td><td>2.50</td><td>|</td><td>0.04</td></tr>
<tr><td>446</td><td>A:ASN 446</td><td></td><td>100.51</td><td>4.35</td><td>|</td><td>-0.02</td></tr>
<tr><td>447</td><td>A:SER 447</td><td></td><td>94.79</td><td>0.00</td><td></td><td>0.00</td></tr>
<tr><td>448</td><td>A:ILE 448</td><td></td><td>112.16</td><td>0.00</td><td></td><td>0.00</td></tr>
<tr><td>449</td><td>A:PHE 449</td><td></td><td>25.19</td><td>0.00</td><td></td><td>0.00</td></tr>
<tr><td>450</td><td>A:GLY 450</td><td></td><td>46.09</td><td>0.00</td><td></td><td>0.00</td></tr>
<tr><td>451</td><td>A:GLY 451</td><td></td><td>83.38</td><td>0.00</td><td></td><td>0.00</td></tr>
<tr><td>452</td><td>A:VAL 452</td><td></td><td>56.98</td><td>0.00</td><td></td><td>0.00</td></tr>
<tr><td>453</td><td>A:GLY 453</td><td></td><td>35.78</td><td>0.00</td><td></td><td>0.00</td></tr>
<tr><td>454</td><td>A:PHE 454</td><td></td><td>91.84</td><td>0.00</td><td></td><td>0.00</td></tr>
<tr><td>455</td><td>A:LEU 455</td><td></td><td>120.38</td><td>0.00</td><td></td><td>0.00</td></tr>
<tr><td>456</td><td>A:PRO 456</td><td></td><td>64.32</td><td>0.00</td><td></td><td>0.00</td></tr>
<tr><td>457</td><td>A:LYS 457</td><td></td><td>35.12</td><td>0.00</td><td></td><td>0.00</td></tr>
<tr><td>458</td><td>A:LEU 458</td><td></td><td>52.81</td><td>0.00</td><td></td><td>0.00</td></tr>
<tr><td>459</td><td>A:LEU 459</td><td></td><td>123.34</td><td>0.00</td><td></td><td>0.00</td></tr>
<tr><td>460</td><td>A:LEU 460</td><td></td><td>78.94</td><td>0.00</td><td></td><td>0.00</td></tr>
<tr><td>461</td><td>A:GLY 461</td><td></td><td>0.14</td><td>0.00</td><td></td><td>0.00</td></tr>
<tr><td>462</td><td>A:VAL 462</td><td></td><td>76.66</td><td>0.00</td><td></td><td>0.00</td></tr>
<tr><td>463</td><td>A:ALA 463</td><td></td><td>50.59</td><td>0.00</td><td></td><td>0.00</td></tr>
<tr><td>464</td><td>A:LEU 464</td><td></td><td>55.72</td><td>0.00</td><td></td><td>0.00</td></tr>
<tr><td>465</td><td>A:ALA 465</td><td></td><td>22.48</td><td>0.00</td><td></td><td>0.00</td></tr>
<tr><td>466</td><td>A:TRP 466</td><td></td><td>154.35</td><td>0.00</td><td></td><td>0.00</td></tr>
<tr><td>467</td><td>A:LEU 467</td><td></td><td>80.28</td><td>0.00</td><td></td><td>0.00</td></tr>
<tr><td>468</td><td>A:GLY 468</td><td></td><td>0.17</td><td>0.00</td><td></td><td>0.00</td></tr>
<tr><td>469</td><td>A:LEU 469</td><td></td><td>123.39</td><td>0.00</td><td></td><td>0.00</td></tr>
<tr><td>470</td><td>A:ASN 470</td><td></td><td>87.85</td><td>0.00</td><td></td><td>0.00</td></tr>
<tr><td>471</td><td>A:MET 471</td><td></td><td>58.58</td><td>0.00</td><td></td><td>0.00</td></tr>
<tr><td>472</td><td>A:ARG 472</td><td></td><td>217.43</td><td>0.00</td><td></td><td>0.00</td></tr>
<tr><td>473</td><td>A:ASN 473</td><td></td><td>59.59</td><td>0.00</td><td></td><td>0.00</td></tr>
<tr><td>474</td><td>A:PRO 474</td><td></td><td>98.38</td><td>0.00</td><td></td><td>0.00</td></tr>
<tr><td>475</td><td>A:THR 475</td><td></td><td>100.49</td><td>0.00</td><td></td><td>0.00</td></tr>
<tr><td>476</td><td>A:MET 476</td><td></td><td>93.99</td><td>0.00</td><td></td><td>0.00</td></tr>
<tr><td>477</td><td>A:SER 477</td><td></td><td>13.07</td><td>0.00</td><td></td><td>0.00</td></tr>
<tr><td>478</td><td>A:MET 478</td><td></td><td>141.64</td><td>0.00</td><td></td><td>0.00</td></tr>
<tr><td>479</td><td>A:SER 479</td><td></td><td>70.17</td><td>0.00</td><td></td><td>0.00</td></tr>
<tr><td>480</td><td>A:PHE 480</td><td></td><td>74.65</td><td>0.00</td><td></td><td>0.00</td></tr>
<tr><td>481</td><td>A:LEU 481</td><td></td><td>60.26</td><td>0.00</td><td></td><td>0.00</td></tr>
<tr><td>482</td><td>A:LEU 482</td><td></td><td>115.41</td><td>0.00</td><td></td><td>0.00</td></tr>
<tr><td>483</td><td>A:ALA 483</td><td></td><td>50.75</td><td>0.00</td><td></td><td>0.00</td></tr>
<tr><td>484</td><td>A:GLY 484</td><td></td><td>0.00</td><td>0.00</td><td></td><td>0.00</td></tr>
<tr><td>485</td><td>A:GLY 485</td><td></td><td>33.16</td><td>0.00</td><td></td><td>0.00</td></tr>
<tr><td>486</td><td>A:LEU 486</td><td></td><td>108.69</td><td>0.00</td><td></td><td>0.00</td></tr>
<tr><td>487</td><td>A:VAL 487</td><td></td><td>16.50</td><td>0.00</td><td></td><td>0.00</td></tr>
<tr><td>488</td><td>A:LEU 488</td><td></td><td>40.84</td><td>0.00</td><td></td><td>0.00</td></tr>
<tr><td>489</td><td>A:ALA 489</td><td></td><td>65.30</td><td>0.00</td><td></td><td>0.00</td></tr>
</table>

490	A:MET 490	81.75	32.03 ||||	0.64
491	A:THR 491	5.80	0.00	0.00
492	A:LEU 492	118.58	0.00	0.00
493	A:GLY 493	63.66	0.80		0.01
494	A:VAL 494	125.71	34.28 |||	0.54

PDBe PISA v1.52 [20/10/2014]

PDBe Protein Data Bank in Europe

is a member of

wwPDB

EMDataBank Unified Data Resource for 3DEM

This website uses cookies. By continuing to browse this site, you are agreeing to the use of our site cookies. To find out more, see our Terms of Use.

OK

PISA Interface.

Session Map (id=179-P6-IE2)

Start | Interfaces | Interface Search | Monomers | Assemblies

interface # 21 in ourmodelfortest2.pdb crystal.

Space symmetry group: P 1

interface #21/96 XML << < > >>

Interface Summary XML

	Structure 1		Structure 2	
**Selection range**	[CPL]B:500		B	
**class**	Ligand		Protein	
**symmetry operation**	x,y,z		x,y,z	
**symmetry ID**	1_555		0_555	
**Number of atoms**				
**interface**	27	96.4%	52	1.4%
**surface**	27	96.4%	2443	65.2%
**total**	28	100.0%	3746	100.0%
**Number of residues**				
**interface**	1	100.0%	19	3.8%
**surface**	1	100.0%	485	98.2%
**total**	1	100.0%	494	100.0%
**Solvent-accessible area, Å**				
**interface**	396.9	60.1%	350.3	1.3%
**total**	660.8	100.0%	27658.7	100.0%
**Solvation energy, kcal/mol**				
**isolated structure**	2.3	100.0%	-446.5	100.0%
**gain on complex formation**	-2.8	-119.8%	-4.6	1.0%
**average gain**	-2.3	-100.0%	-1.9	0.4%
**P-value**	0.473		0.120	

View structure 1 | interface | structure 2

Download

structure 1 | interface | structure 2

This interface scored

0.350

in Complex Formation Significance Score (CSS).

CSS ranges from 0 to 1 as interface relevance to complex formation increases.

Achieved CSS implies that the interface plays an auxiliary role in complex formatior

No disulfide bonds found

No covalent bonds found

No hydrogen bonds found

No salt bridges found

Interfacing residues (not a contact table) XML Display level: Residues

Inaccessible residues HSDC Residues making **H**ydrogen/**D**isulphide bond, **S**alt bridge or **C**ovalent link

Solvent-accessible residues Interfacing residues

ASA Accessible Surface Area, Å² **BSA** Buried Surface Area, Å² **Δ^{i}G** Solvation energy effect, kcal/mol |||| Buried area percentage, one bar per 10%

##	Structure 1	HSDC	ASA	BSA	Δ^{i}G
1	B:CPL 500		660.81	396.94 ||||||	2.80

##	Structure 2	HSDC	ASA	BSA	Δ^{i}G
1	B:SER 1		13.19	0.00	0.00
2	B:ARG 2		62.08	0.00	0.00
3	B:CYS 3		0.00	0.00	0.00
4	B:THR 4		26.46	0.00	0.00
5	B:HIS 5		71.79	0.00	0.00

6	B:LEU	6	49.54	0.00	0.00
7	B:GLU	7	172.99	0.00	0.00
8	B:ASN	8	67.62	0.00	0.00
9	B:ARG	9	9.60	0.00	0.00
10	B:ASP	10	14.86	0.00	0.00
11	B:PHE	11	72.10	0.00	0.00
12	B:VAL	12	19.22	0.00	0.00
13	B:THR	13	95.23	0.00	0.00
14	B:GLY	14	18.51	0.00	0.00
15	B:THR	15	97.73	0.00	0.00
16	B:GLN	16	159.83	0.00	0.00
17	B:GLY	17	66.54	0.00	0.00
18	B:THR	18	51.44	0.00	0.00
19	B:THR	19	65.81	0.00	0.00
20	B:ARG	20	144.33	0.00	0.00
21	B:VAL	21	17.37	0.00	0.00
22	B:THR	22	26.28	0.00	0.00
23	B:LEU	23	0.17	0.00	0.00
24	B:VAL	24	3.44	0.00	0.00
25	B:LEU	25	4.19	0.00	0.00
26	B:GLU	26	41.84	0.00	0.00
27	B:LEU	27	34.43	0.00	0.00
28	B:GLY	28	54.39	0.00	0.00
29	B:GLY	29	7.41	0.00	0.00
30	B:CYS	30	9.61	0.00	0.00
31	B:VAL	31	6.52	0.00	0.00
32	B:THR	32	0.24	0.00	0.00
33	B:ILE	33	4.52	0.00	0.00
34	B:THR	34	28.86	0.00	0.00
35	B:ALA	35	18.64	0.00	0.00
36	B:GLU	36	90.86	0.00	0.00
37	B:GLY	37	49.68	0.00	0.00
38	B:LYS	38	59.39	0.00	0.00
39	B:PRO	39	11.15	0.00	0.00
40	B:SER	40	3.71	0.00	0.00
41	B:MET	41	0.50	0.00	0.00
42	B:ASP	42	0.00	0.00	0.00
43	B:VAL	43	1.50	0.00	0.00
44	B:TRP	44	11.17	0.00	0.00
45	B:LEU	45	4.80	0.00	0.00
46	B:ASP	46	63.90	0.00	0.00
47	B:ALA	47	16.50	0.00	0.00
48	B:ILE	48	0.17	0.00	0.00
49	B:TYR	49	42.21	0.00	0.00
50	B:GLN	50	6.18	0.00	0.00
51	B:GLU	51	86.82	0.00	0.00
52	B:ASN	52	106.48	0.00	0.00
53	B:PRO	53	14.50	0.00	0.00
54	B:ALA	54	51.97	0.00	0.00
55	B:LYS	55	107.55	0.00	0.00
56	B:THR	56	36.79	0.00	0.00
57	B:ARG	57	53.86	0.00	0.00
58	B:GLU	58	20.06	0.00	0.00
59	B:TYR	59	2.60	0.00	0.00
60	B:CYS	60	2.65	0.00	0.00
61	B:LEU	61	4.69	0.00	0.00
62	B:HIS	62	50.03	0.00	0.00
63	B:ALA	63	8.96	0.00	0.00
64	B:LYS	64	116.66	0.00	0.00
65	B:LEU	65	46.30	0.00	0.00
66	B:SER	66	55.95	0.00	0.00
67	B:ASP	67	76.08	0.00	0.00
68	B:THR	68	86.97	0.00	0.00
69	B:LYS	69	96.96	0.00	0.00
70	B:VAL	70	62.09	0.00	0.00
71	B:ALA	71	30.26	0.00	0.00
72	B:ALA	72	25.65	0.00	0.00
73	B:ARG	73	108.62	0.00	0.00
74	B:CYS	74	34.99	0.00	0.00

75	B:PRO 75	29.22	0.00	0.00
76	B:THR 76	90.66	0.00	0.00
77	B:MET 77	130.59	0.00	0.00
78	B:GLY 78	33.91	0.00	0.00
79	B:PRO 79	86.74	0.00	0.00
80	B:ALA 80	1.83	0.00	0.00
81	B:THR 81	95.63	0.00	0.00
82	B:LEU 82	37.13	0.00	0.00
83	B:ALA 83	72.12	0.00	0.00
84	B:GLU 84	33.22	0.00	0.00
85	B:GLU 85	59.77	0.00	0.00
86	B:HIS 86	165.39	0.00	0.00
87	B:GLN 87	111.68	0.00	0.00
88	B:GLY 88	49.83	0.00	0.00
89	B:GLY 89	23.11	0.00	0.00
90	B:THR 90	29.93	0.00	0.00
91	B:VAL 91	16.39	0.00	0.00
92	B:CYS 92	39.69	0.00	0.00
93	B:LYS 93	88.51	0.00	0.00
94	B:ARG 94	106.99	0.00	0.00
95	B:ASP 95	52.11	0.00	0.00
96	B:GLN 96	108.11	0.00	0.00
97	B:SER 97	3.35	0.00	0.00
98	B:ASP 98	97.65	0.00	0.00
99	B:ARG 99	32.90	0.00	0.00
100	B:GLY 100	4.01	0.00	0.00
101	B:TRP 101	177.25	0.00	0.00
102	B:GLY 102	76.61	0.00	0.00
103	B:ASN 103	41.34	0.00	0.00
104	B:HIS 104	170.10	0.00	0.00
105	B:CYS 105	6.20	0.00	0.00
106	B:GLY 106	57.55	0.00	0.00
107	B:LEU 107	100.01	0.00	0.00
108	B:PHE 108	138.71	0.00	0.00
109	B:GLY 109	29.80	0.00	0.00
110	B:LYS 110	114.93	0.00	0.00
111	B:GLY 111	0.82	0.00	0.00
112	B:SER 112	25.30	0.00	0.00
113	B:ILE 113	0.12	0.00	0.00
114	B:VAL 114	0.50	0.00	0.00
115	B:ALA 115	2.32	0.00	0.00
116	B:CYS 116	2.09	0.00	0.00
117	B:VAL 117	2.98	0.00	0.00
118	B:LYS 118	82.26	0.00	0.00
119	B:ALA 119	13.89	0.00	0.00
120	B:ALA 120	54.15	0.00	0.00
121	B:CYS 121	22.94	0.00	0.00
122	B:GLU 122	56.40	0.00	0.00
123	B:ALA 123	76.55	0.00	0.00
124	B:LYS 124	144.92	0.00	0.00
125	B:LYS 125	70.16	0.00	0.00
126	B:LYS 126	76.17	0.00	0.00
127	B:ALA 127	3.16	0.00	0.00
128	B:THR 128	18.86	0.00	0.00
129	B:GLY 129	0.00	0.00	0.00
130	B:HIS 130	7.88	0.00	0.00
131	B:VAL 131	45.50	0.00	0.00
132	B:TYR 132	18.90	0.00	0.00
133	B:ASP 133	66.44	0.00	0.00
134	B:ALA 134	53.14	0.00	0.00
135	B:ASN 135	113.50	0.00	0.00
136	B:LYS 136	125.12	0.00	0.00
137	B:ILE 137	2.10	0.00	0.00
138	B:VAL 138	28.40	0.00	0.00
139	B:TYR 139	2.99	0.00	0.00
140	B:THR 140	23.67	0.00	0.00
141	B:VAL 141	0.67	0.00	0.00
142	B:LYS 142	42.00	0.00	0.00
143	B:VAL 143	0.82	0.00	0.00

144	B:GLU 144	0.98	0.00	0.00
145	B:PRO 145	5.08	0.00	0.00
146	B:HIS 146	18.49	0.00	0.00
147	B:THR 147	35.71	0.00	0.00
148	B:GLY 148	19.54	0.00	0.00
149	B:ASP 149	67.55	0.00	0.00
150	B:TYR 150	68.66	0.00	0.00
151	B:VAL 151	38.49	0.00	0.00
152	B:ALA 152	51.62	0.00	0.00
153	B:ALA 153	90.69	0.00	0.00
154	B:ASN 154	138.75	0.00	0.00
155	B:GLU 155	88.06	0.00	0.00
156	B:THR 156	129.25	0.00	0.00
157	B:HIS 157	25.58	0.00	0.00
158	B:SER 158	115.86	0.00	0.00
159	B:GLY 159	17.57	0.00	0.00
160	B:ARG 160	76.96	0.00	0.00
161	B:LYS 161	75.13	0.00	0.00
162	B:THR 162	81.19	0.00	0.00
163	B:ALA 163	5.50	0.00	0.00
164	B:SER 164	72.97	0.00	0.00
165	B:PHE 165	1.29	0.00	0.00
166	B:THR 166	36.71	0.00	0.00
167	B:VAL 167	83.35	0.00	0.00
168	B:SER 168	74.77	0.00	0.00
169	B:SER 169	74.87	0.00	0.00
170	B:GLU 170	65.76	0.00	0.00
171	B:LYS 171	123.55	0.00	0.00
172	B:THR 172	40.75	0.00	0.00
173	B:ILE 173	93.30	0.00	0.00
174	B:LEU 174	17.67	0.00	0.00
175	B:THR 175	90.16	0.00	0.00
176	B:MET 176	12.22	0.00	0.00
177	B:GLY 177	58.43	0.00	0.00
178	B:GLU 178	102.49	0.00	0.00
179	B:TYR 179	29.99	0.00	0.00
180	B:GLY 180	11.49	0.00	0.00
181	B:ASP 181	36.75	0.00	0.00
182	B:VAL 182	2.77	0.00	0.00
183	B:SER 183	26.79	0.00	0.00
184	B:LEU 184	1.28	0.00	0.00
185	B:LEU 185	30.39	0.00	0.00
186	B:CYS 186	2.33	0.00	0.00
187	B:ARG 187	126.37	0.00	0.00
188	B:VAL 188	15.85	0.00	0.00
189	B:ALA 189	99.88	0.00	0.00
190	B:SER 190	34.31	0.00	0.00
191	B:GLY 191	10.49	0.00	0.00
192	B:VAL 192	21.78	0.00	0.00
193	B:ASP 193	88.32	0.00	0.00
194	B:LEU 194	36.05	0.00	0.00
195	B:ALA 195	75.11	0.00	0.00
196	B:GLN 196	82.27	0.00	0.00
197	B:THR 197	18.06	0.00	0.00
198	B:VAL 198	2.02	0.00	0.00
199	B:ILE 199	2.35	0.00	0.00
200	B:LEU 200	1.17	0.00	0.00
201	B:GLU 201	19.84	0.00	0.00
202	B:LEU 202	25.30	0.00	0.00
203	B:ASP 203	40.77	0.00	0.00
204	B:LYS 204	103.91	0.00	0.00
205	B:THR 205	110.40	0.00	0.00
206	B:VAL 206	68.15	0.00	0.00
207	B:GLU 207	133.90	0.00	0.00
208	B:HIS 208	177.89	0.00	0.00
209	B:LEU 209	71.25	0.00	0.00
210	B:PRO 210	50.04	0.00	0.00
211	B:THR 211	29.12	0.00	0.00
212	B:ALA 212	0.00	0.00	0.00

213	B:TRP 213	18.24	0.00	0.00
214	B:GLN 214	28.24	0.00	0.00
215	B:VAL 215	3.13	0.00	0.00
216	B:HIS 216	89.31	0.00	0.00
217	B:ARG 217	87.77	0.00	0.00
218	B:ASP 218	79.23	0.00	0.00
219	B:TRP 219	85.95	0.00	0.00
220	B:PHE 220	0.94	0.00	0.00
221	B:ASN 221	54.50	0.00	0.00
222	B:ASP 222	114.50	0.00	0.00
223	B:LEU 223	40.18	0.00	0.00
224	B:ALA 224	71.61	0.00	0.00
225	B:LEU 225	21.89	0.00	0.00
226	B:PRO 226	4.68	0.00	0.00
227	B:TRP 227	55.04	0.00	0.00
228	B:LYS 228	27.66	0.00	0.00
229	B:HIS 229	106.09	0.00	0.00
230	B:GLU 230	123.89	0.00	0.00
231	B:GLY 231	75.70	0.00	0.00
232	B:ALA 232	49.67	0.00	0.00
233	B:GLN 233	156.83	0.00	0.00
234	B:ASN 234	69.01	0.00	0.00
235	B:TRP 235	34.05	0.00	0.00
236	B:ASN 236	60.76	0.00	0.00
237	B:ASN 237	68.72	0.00	0.00
238	B:ALA 238	15.18	0.00	0.00
239	B:GLU 239	120.70	0.00	0.00
240	B:ARG 240	102.53	0.00	0.00
241	B:LEU 241	6.84	0.00	0.00
242	B:VAL 242	4.07	0.00	0.00
243	B:GLU 243	99.22	0.00	0.00
244	B:PHE 244	34.93	0.00	0.00
245	B:GLY 245	16.83	0.00	0.00
246	B:ALA 246	91.45	0.00	0.00
247	B:PRO 247	32.82	0.00	0.00
248	B:HIS 248	122.22	0.00	0.00
249	B:ALA 249	27.13	0.00	0.00
250	B:VAL 250	76.20	0.00	0.00
251	B:LYS 251	131.22	0.00	0.00
252	B:MET 252	19.74	0.00	0.00
253	B:ASP 253	61.71	0.00	0.00
254	B:VAL 254	43.18	0.00	0.00
255	B:TYR 255	117.45	0.00	0.00
256	B:ASN 256	69.90	0.00	0.00
257	B:LEU 257	94.79	0.00	0.00
258	B:GLY 258	24.65	0.00	0.00
259	B:ASP 259	59.91	0.00	0.00
260	B:GLN 260	37.12	0.00	0.00
261	B:THR 261	34.99	0.00	0.00
262	B:GLY 262	55.92	0.00	0.00
263	B:VAL 263	100.92	0.00	0.00
264	B:LEU 264	14.32	0.00	0.00
265	B:LEU 265	53.67	0.00	0.00
266	B:LYS 266	161.26	0.00	0.00
267	B:ALA 267	64.99	0.00	0.00
268	B:LEU 268	5.89	0.00	0.00
269	B:ALA 269	87.04	0.00	0.00
270	B:GLY 270	76.19	0.00	0.00
271	B:VAL 271	42.49	0.00	0.00
272	B:PRO 272	68.06	0.00	0.00
273	B:VAL 273	75.39	0.00	0.00
274	B:ALA 274	1.15	0.00	0.00
275	B:HIS 275	73.72	0.00	0.00
276	B:ILE 276	12.58	0.00	0.00
277	B:GLU 277	104.42	0.00	0.00
278	B:GLY 278	51.76	0.00	0.00
279	B:THR 279	72.56	0.00	0.00
280	B:LYS 280	64.90	0.00	0.00
281	B:TYR 281	22.54	0.00	0.00

282	B:HIS 282	24.77	0.00	0.00
283	B:LEU 283	2.69	0.00	0.00
284	B:LYS 284	119.43	0.00	0.00
285	B:SER 285	52.89	0.00	0.00
286	B:GLY 286	15.48	0.00	0.00
287	B:HIS 287	28.81	0.00	0.00
288	B:VAL 288	1.84	0.00	0.00
289	B:THR 289	22.84	0.00	0.00
290	B:CYS 290	2.99	0.00	0.00
291	B:GLU 291	55.77	0.00	0.00
292	B:VAL 292	0.99	0.00	0.00
293	B:GLY 293	10.72	0.00	0.00
294	B:LEU 294	12.17	0.00	0.00
295	B:GLU 295	121.57	0.00	0.00
296	B:LYS 296	140.33	0.00	0.00
297	B:LEU 297	9.60	0.00	0.00
298	B:LYS 298	123.85	0.00	0.00
299	B:MET 299	48.56	0.00	0.00
300	B:LYS 300	56.61	0.00	0.00
301	B:GLY 301	15.32	0.00	0.00
302	B:LEU 302	97.49	0.00	0.00
303	B:THR 303	129.80	0.00	0.00
304	B:TYR 304	75.60	0.00	0.00
305	B:THR 305	103.57	0.00	0.00
306	B:MET 306	123.11	0.00	0.00
307	B:CYS 307	9.02	0.00	0.00
308	B:ASP 308	66.38	0.00	0.00
309	B:LYS 309	114.69	0.00	0.00
310	B:THR 310	93.77	0.00	0.00
311	B:LYS 311	91.38	0.00	0.00
312	B:PHE 312	11.54	0.00	0.00
313	B:THR 313	69.77	0.00	0.00
314	B:TRP 314	53.43	0.00	0.00
315	B:LYS 315	136.69	0.00	0.00
316	B:ARG 316	150.20	0.00	0.00
317	B:ALA 317	50.59	0.00	0.00
318	B:PRO 318	7.48	0.00	0.00
319	B:THR 319	72.06	0.00	0.00
320	B:ASP 320	66.32	0.00	0.00
321	B:SER 321	37.47	0.00	0.00
322	B:GLY 322	63.67	0.00	0.00
323	B:HIS 323	37.82	0.00	0.00
324	B:ASP 324	58.99	0.00	0.00
325	B:THR 325	2.57	0.00	0.00
326	B:VAL 326	1.18	0.00	0.00
327	B:VAL 327	30.97	0.00	0.00
328	B:MET 328	3.81	0.00	0.00
329	B:GLU 329	23.93	0.00	0.00
330	B:VAL 330	5.94	0.00	0.00
331	B:THR 331	59.84	0.00	0.00
332	B:PHE 332	17.44	0.00	0.00
333	B:SER 333	88.83	0.00	0.00
334	B:GLY 334	17.04	0.00	0.00
335	B:THR 335	116.38	0.00	0.00
336	B:LYS 336	81.15	0.00	0.00
337	B:PRO 337	64.97	0.00	0.00
338	B:CYS 338	4.68	0.00	0.00
339	B:ARG 339	76.69	0.00	0.00
340	B:ILE 340	6.74	0.00	0.00
341	B:PRO 341	32.53	0.00	0.00
342	B:VAL 342	17.49	0.00	0.00
343	B:ARG 343	86.65	0.00	0.00
344	B:ALA 344	0.00	0.00	0.00
345	B:VAL 345	15.86	0.00	0.00
346	B:ALA 346	27.11	0.00	0.00
347	B:HIS 347	144.76	0.00	0.00
348	B:GLY 348	74.77	0.00	0.00
349	B:SER 349	59.80	0.00	0.00
350	B:PRO 350	115.21	0.00	0.00

```
351  B:ASP 351       85.07     0.00              0.00
352  B:VAL 352       90.82     0.00              0.00
353  B:ASN 353       80.15     0.00              0.00
354  B:VAL 354       52.10     0.00              0.00
355  B:ALA 355       16.65     0.00              0.00
356  B:MET 356      134.44     0.00              0.00
357  B:LEU 357       59.91     0.00              0.00
358  B:ILE 358       25.89     0.00              0.00
359  B:THR 359        5.36     0.00              0.00
360  B:PRO 360       40.55     0.00              0.00
361  B:ASN 361       38.07     0.00              0.00
362  B:PRO 362       11.88     0.00              0.00
363  B:THR 363       16.12     0.00              0.00
364  B:ILE 364       10.74     0.00              0.00
365  B:GLU 365       45.26     0.00              0.00
366  B:ASN 366      112.77     0.00              0.00
367  B:ASN 367      161.27     0.00              0.00
368  B:GLY 368       29.38     0.00              0.00
369  B:GLY 369       30.86     0.00              0.00
370  B:GLY 370        2.16     0.00              0.00
371  B:PHE 371       17.00     0.00              0.00
372  B:ILE 372        0.50     0.00              0.00
373  B:GLU 373        0.99     0.00              0.00
374  B:MET 374        1.32     0.00              0.00
375  B:GLN 375       53.70     0.00              0.00
376  B:LEU 376        4.26     0.00              0.00
377  B:PRO 377       40.59     0.00              0.00
378  B:PRO 378       50.59     0.00              0.00
379  B:GLY 379       25.23     0.00              0.00
380  B:ASP 380       71.04     0.00              0.00
381  B:ASN 381        1.62     0.00              0.00
382  B:ILE 382       56.68     0.00              0.00
383  B:ILE 383        0.50     0.00              0.00
384  B:TYR 384       63.45     0.00              0.00
385  B:VAL 385        0.00     0.00              0.00
386  B:GLY 386       11.22     0.00              0.00
387  B:GLU 387      126.03     0.00              0.00
388  B:LEU 388       49.43     0.00              0.00
389  B:SER 389       49.28     0.00              0.00
390  B:HIS 390       61.92     0.00              0.00
391  B:GLN 391      127.30     0.00              0.00
392  B:TRP 392       36.38     0.00              0.00
393  B:PHE 393      135.35     0.00              0.00
394  B:GLN 394       10.28     0.00              0.00
395  B:LYS 395      133.81     0.00              0.00
396  B:GLY 396       61.47     0.00              0.00
397  B:SER 397       55.71     0.00              0.00
398  B:SER 398       70.64     0.00              0.00
399  B:ILE 399      138.48     0.00              0.00
400  B:GLY 400       36.06     0.00              0.00
401  B:ARG 401       60.11     0.00              0.00
402  B:VAL 402       91.08     0.00              0.00
403  B:PHE 403      138.16     0.00              0.00
404  B:GLN 404       84.90     0.00              0.00
405  B:LYS 405      143.70     0.00              0.00
406  B:THR 406       73.98     0.00              0.00
407  B:LYS 407      111.39     0.00              0.00
408  B:LYS 408       66.82     0.00              0.00
409  B:GLY 409       32.84     0.00              0.00
410  B:ILE 410      107.28    15.90 ||          0.25
411  B:GLU 411      107.81     0.00              0.00
412  B:ARG 412       43.34     0.00              0.00
413  B:LEU 413       55.39    28.00 ||||||      0.40
414  B:THR 414       66.49    37.59 ||||||      0.15
415  B:VAL 415       98.46    14.68 ||         -0.16
416  B:ILE 416       17.51     3.30 ||          0.04
417  B:GLY 417       19.64    19.64 ||||||||||  0.29
418  B:GLU 418       59.53     6.51 ||         -0.07
419  B:HIS 419       45.77     0.00              0.00
```

420	B:ALA 420	14.57	0.00		0.00
421	B:TRP 421	90.12	14.77	||	0.24
422	B:ASP 422	27.28	0.00		0.00
423	B:PHE 423	39.69	0.00		0.00
424	B:GLY 424	30.50	0.00		0.00
425	B:SER 425	29.41	0.00		0.00
426	B:ALA 426	106.61	0.00		0.00
427	B:GLY 427	46.17	0.00		0.00
428	B:GLY 428	53.59	0.00		0.00
429	B:PHE 429	167.72	0.00		0.00
430	B:LEU 430	146.32	0.00		0.00
431	B:SER 431	14.03	0.00		0.00
432	B:SER 432	65.78	0.00		0.00
433	B:ILE 433	80.16	0.00		0.00
434	B:GLY 434	12.11	1.24	||	-0.00
435	B:LYS 435	71.52	0.00		0.00
436	B:ALA 436	50.68	0.00		0.00
437	B:VAL 437	87.38	13.23	||	0.21
438	B:HIS 438	54.99	52.09	||||||||||	0.70
439	B:THR 439	64.18	0.00		0.00
440	B:VAL 440	103.08	0.00		0.00
441	B:LEU 441	84.63	16.15	||	0.26
442	B:GLY 442	27.86	12.77	|||||	0.20
443	B:GLY 443	39.09	0.00		0.00
444	B:ALA 444	57.91	0.00		0.00
445	B:PHE 445	40.43	1.87	|	0.03
446	B:ASN 446	101.94	1.59	|	0.02
447	B:SER 447	90.99	0.00		0.00
448	B:ILE 448	111.28	0.00		0.00
449	B:PHE 449	36.26	0.00		0.00
450	B:GLY 450	48.58	0.00		0.00
451	B:GLY 451	90.34	0.00		0.00
452	B:VAL 452	63.68	0.00		0.00
453	B:GLY 453	34.37	0.00		0.00
454	B:PHE 454	138.29	0.00		0.00
455	B:LEU 455	108.88	0.00		0.00
456	B:PRO 456	53.43	0.00		0.00
457	B:LYS 457	32.97	0.00		0.00
458	B:LEU 458	57.56	0.00		0.00
459	B:LEU 459	112.68	0.00		0.00
460	B:LEU 460	83.58	0.00		0.00
461	B:GLY 461	0.00	0.00		0.00
462	B:VAL 462	88.05	0.00		0.00
463	B:ALA 463	48.01	0.00		0.00
464	B:LEU 464	48.38	0.00		0.00
465	B:ALA 465	32.39	0.00		0.00
466	B:TRP 466	156.33	0.00		0.00
467	B:LEU 467	69.67	0.00		0.00
468	B:GLY 468	0.00	0.00		0.00
469	B:LEU 469	138.86	0.00		0.00
470	B:ASN 470	96.11	0.00		0.00
471	B:MET 471	45.09	0.00		0.00
472	B:ARG 472	213.35	0.00		0.00
473	B:ASN 473	52.02	0.00		0.00
474	B:PRO 474	109.58	0.00		0.00
475	B:THR 475	96.15	0.00		0.00
476	B:MET 476	90.31	0.00		0.00
477	B:SER 477	16.45	0.00		0.00
478	B:MET 478	127.22	0.00		0.00
479	B:SER 479	58.68	0.00		0.00
480	B:PHE 480	58.08	0.00		0.00
481	B:LEU 481	52.52	0.00		0.00
482	B:LEU 482	115.16	0.00		0.00
483	B:ALA 483	42.73	0.00		0.00
484	B:GLY 484	0.00	0.00		0.00
485	B:GLY 485	35.34	0.00		0.00
486	B:LEU 486	68.85	0.00		0.00
487	B:VAL 487	2.96	0.00		0.00
488	B:LEU 488	49.93	0.00		0.00

489	B:ALA 489	64.21	12.12 ||	0.08
490	B:MET 490	63.51	50.08 ||||||||	1.22
491	B:THR 491	17.31	0.00	0.00
492	B:LEU 492	150.75	0.00	0.00
493	B:GLY 493	54.57	15.51 |||	0.25
494	B:VAL 494	145.39	33.26 |||	0.52

PDBe PISA v1.52 [20/10/2014]

PDBe Protein Data Bank in Europe

is a member of

wwPDB

EMDataBank Unified Data Resource for 3DEM

This website uses cookies. By continuing to browse this site, you are agreeing to the use of our site cookies. To find out more, see our Terms of Use.

OK

PISA Interface.

interface # 24 in ourmodelfortest2.pdb crystal.

Space symmetry group: P 1

Interface Summary XML

	Structure 1		Structure 2	
**Selection range**	[CPL]C:500		C	
**class**	Ligand		Protein	
**symmetry operation**	x,y,z		x,y,z	
**symmetry ID**	1_555		0_555	
**Number of atoms**				
**interface**	26	92.9%	46	1.2%
**surface**	27	96.4%	2456	65.6%
**total**	28	100.0%	3746	100.0%
**Number of residues**				
**interface**	1	100.0%	18	3.6%
**surface**	1	100.0%	488	98.8%
**total**	1	100.0%	494	100.0%
**Solvent-accessible area, Å**				
**interface**	363.5	54.9%	334.4	1.2%
**total**	661.8	100.0%	28155.3	100.0%
**Solvation energy, kcal/mol**				
**isolated structure**	1.6	100.0%	-443.9	100.0%
**gain on complex formation**	-2.2	-136.9%	-3.7	0.8%
**average gain**	-1.5	-96.3%	-1.7	0.4%
**P-value**	0.454		0.187	

View: structure 1 | interface | structure 2

Download: structure 1 | interface | structure 2

This interface scored

0.590

in Complex Formation Significance Score (CSS).

CSS ranges from 0 to 1 as interface relevance to complex formation increases.

Achieved CSS implies that the interface plays an essential role in complex formatior

No disulfide bonds found

No covalent bonds found

No hydrogen bonds found

No salt bridges found

Interfacing residues (not a contact table) XML Display level: Residues

Inaccessible residues | HSDC Residues making **H**ydrogen/**D**isulphide bond, **S**alt bridge or **C**ovalent link

Solvent-accessible residues | Interfacing residues

ASA Accessible Surface Area, Å² **BSA** Buried Surface Area, Å² **ΔⁱG** Solvation energy effect, kcal/mol |||| Buried area percentage, one bar per 10%

##	Structure 1	HSDC	ASA	BSA	Δ^iG
1	C:CPL 500		661.85	363.53 |||||	2.17

##	Structure 2	HSDC	ASA	BSA	Δ^iG
1	C:SER 1		10.29	0.00	0.00
2	C:ARG 2		74.07	0.00	0.00
3	C:CYS 3		0.00	0.00	0.00
4	C:THR 4		36.19	0.00	0.00
5	C:HIS 5		62.95	0.00	0.00

6	C:LEU	6	50.30	0.00	0.00
7	C:GLU	7	173.10	0.00	0.00
8	C:ASN	8	71.91	0.00	0.00
9	C:ARG	9	10.42	0.00	0.00
10	C:ASP	10	25.94	0.00	0.00
11	C:PHE	11	66.35	0.00	0.00
12	C:VAL	12	20.40	0.00	0.00
13	C:THR	13	102.91	0.00	0.00
14	C:GLY	14	17.76	0.00	0.00
15	C:THR	15	92.86	0.00	0.00
16	C:GLN	16	122.11	0.00	0.00
17	C:GLY	17	76.44	0.00	0.00
18	C:THR	18	56.60	0.00	0.00
19	C:THR	19	53.54	0.00	0.00
20	C:ARG	20	170.92	0.00	0.00
21	C:VAL	21	22.41	0.00	0.00
22	C:THR	22	20.38	0.00	0.00
23	C:LEU	23	1.84	0.00	0.00
24	C:VAL	24	1.72	0.00	0.00
25	C:LEU	25	3.52	0.00	0.00
26	C:GLU	26	48.04	0.00	0.00
27	C:LEU	27	31.70	0.00	0.00
28	C:GLY	28	56.10	0.00	0.00
29	C:GLY	29	8.90	0.00	0.00
30	C:CYS	30	6.26	0.00	0.00
31	C:VAL	31	7.87	0.00	0.00
32	C:THR	32	0.17	0.00	0.00
33	C:ILE	33	5.27	0.00	0.00
34	C:THR	34	34.29	0.00	0.00
35	C:ALA	35	9.84	0.00	0.00
36	C:GLU	36	147.14	0.00	0.00
37	C:GLY	37	36.26	0.00	0.00
38	C:LYS	38	55.88	0.00	0.00
39	C:PRO	39	3.54	0.00	0.00
40	C:SER	40	1.54	0.00	0.00
41	C:MET	41	1.00	0.00	0.00
42	C:ASP	42	0.00	0.00	0.00
43	C:VAL	43	2.17	0.00	0.00
44	C:TRP	44	8.77	0.00	0.00
45	C:LEU	45	3.36	0.00	0.00
46	C:ASP	46	59.00	0.00	0.00
47	C:ALA	47	16.81	0.00	0.00
48	C:ILE	48	0.34	0.00	0.00
49	C:TYR	49	41.84	0.00	0.00
50	C:GLN	50	4.34	0.00	0.00
51	C:GLU	51	91.85	0.00	0.00
52	C:ASN	52	102.69	0.00	0.00
53	C:PRO	53	13.15	0.00	0.00
54	C:ALA	54	54.43	0.00	0.00
55	C:LYS	55	112.86	0.00	0.00
56	C:THR	56	50.77	0.00	0.00
57	C:ARG	57	57.95	0.00	0.00
58	C:GLU	58	12.29	0.00	0.00
59	C:TYR	59	1.93	0.00	0.00
60	C:CYS	60	0.17	0.00	0.00
61	C:LEU	61	0.33	0.00	0.00
62	C:HIS	62	45.88	0.00	0.00
63	C:ALA	63	7.38	0.00	0.00
64	C:LYS	64	108.37	0.00	0.00
65	C:LEU	65	43.42	0.00	0.00
66	C:SER	66	55.99	0.00	0.00
67	C:ASP	67	99.59	0.00	0.00
68	C:THR	68	84.76	0.00	0.00
69	C:LYS	69	93.25	0.00	0.00
70	C:VAL	70	66.15	0.00	0.00
71	C:ALA	71	32.41	0.00	0.00
72	C:ALA	72	30.60	0.00	0.00
73	C:ARG	73	107.47	0.00	0.00
74	C:CYS	74	30.29	0.00	0.00

75	C:PRO	75	38.73	0.00	0.00
76	C:THR	76	94.73	0.00	0.00
77	C:MET	77	125.15	0.00	0.00
78	C:GLY	78	38.73	0.00	0.00
79	C:PRO	79	87.31	0.00	0.00
80	C:ALA	80	2.66	0.00	0.00
81	C:THR	81	95.74	0.00	0.00
82	C:LEU	82	33.34	0.00	0.00
83	C:ALA	83	81.53	0.00	0.00
84	C:GLU	84	32.25	0.00	0.00
85	C:GLU	85	50.27	0.00	0.00
86	C:HIS	86	168.30	0.00	0.00
87	C:GLN	87	126.91	0.00	0.00
88	C:GLY	88	49.47	0.00	0.00
89	C:GLY	89	22.15	0.00	0.00
90	C:THR	90	33.69	0.00	0.00
91	C:VAL	91	14.56	0.00	0.00
92	C:CYS	92	42.37	0.00	0.00
93	C:LYS	93	89.07	0.00	0.00
94	C:ARG	94	100.42	0.00	0.00
95	C:ASP	95	59.65	0.00	0.00
96	C:GLN	96	110.55	0.00	0.00
97	C:SER	97	3.53	0.00	0.00
98	C:ASP	98	97.06	0.00	0.00
99	C:ARG	99	31.02	0.00	0.00
100	C:GLY	100	3.68	0.00	0.00
101	C:TRP	101	185.19	0.00	0.00
102	C:GLY	102	69.86	0.00	0.00
103	C:ASN	103	51.54	0.00	0.00
104	C:HIS	104	174.45	0.00	0.00
105	C:CYS	105	10.24	0.00	0.00
106	C:GLY	106	62.13	0.00	0.00
107	C:LEU	107	94.61	0.00	0.00
108	C:PHE	108	142.39	0.00	0.00
109	C:GLY	109	33.05	0.00	0.00
110	C:LYS	110	125.61	0.00	0.00
111	C:GLY	111	6.72	0.00	0.00
112	C:SER	112	16.29	0.00	0.00
113	C:ILE	113	0.00	0.00	0.00
114	C:VAL	114	1.01	0.00	0.00
115	C:ALA	115	1.01	0.00	0.00
116	C:CYS	116	0.66	0.00	0.00
117	C:VAL	117	3.18	0.00	0.00
118	C:LYS	118	103.54	0.00	0.00
119	C:ALA	119	16.56	0.00	0.00
120	C:ALA	120	46.71	0.00	0.00
121	C:CYS	121	29.43	0.00	0.00
122	C:GLU	122	72.17	0.00	0.00
123	C:ALA	123	85.83	0.00	0.00
124	C:LYS	124	134.45	0.00	0.00
125	C:LYS	125	54.94	0.00	0.00
126	C:LYS	126	75.11	0.00	0.00
127	C:ALA	127	0.51	0.00	0.00
128	C:THR	128	21.19	0.00	0.00
129	C:GLY	129	0.00	0.00	0.00
130	C:HIS	130	5.13	0.00	0.00
131	C:VAL	131	42.27	0.00	0.00
132	C:TYR	132	16.66	0.00	0.00
133	C:ASP	133	56.11	0.00	0.00
134	C:ALA	134	44.73	0.00	0.00
135	C:ASN	135	112.13	0.00	0.00
136	C:LYS	136	127.79	0.00	0.00
137	C:ILE	137	0.51	0.00	0.00
138	C:VAL	138	27.33	0.00	0.00
139	C:TYR	139	1.24	0.00	0.00
140	C:THR	140	22.57	0.00	0.00
141	C:VAL	141	0.82	0.00	0.00
142	C:LYS	142	45.41	0.00	0.00
143	C:VAL	143	2.76	0.00	0.00

144	C:GLU 144	1.11	0.00	0.00
145	C:PRO 145	4.63	0.00	0.00
146	C:HIS 146	7.01	0.00	0.00
147	C:THR 147	55.75	0.00	0.00
148	C:GLY 148	15.03	0.00	0.00
149	C:ASP 149	55.31	0.00	0.00
150	C:TYR 150	78.29	0.00	0.00
151	C:VAL 151	41.66	0.00	0.00
152	C:ALA 152	60.84	0.00	0.00
153	C:ALA 153	86.84	0.00	0.00
154	C:ASN 154	143.30	0.00	0.00
155	C:GLU 155	90.84	0.00	0.00
156	C:THR 156	116.46	0.00	0.00
157	C:HIS 157	25.94	0.00	0.00
158	C:SER 158	112.66	0.00	0.00
159	C:GLY 159	19.92	0.00	0.00
160	C:ARG 160	71.26	0.00	0.00
161	C:LYS 161	78.14	0.00	0.00
162	C:THR 162	78.08	0.00	0.00
163	C:ALA 163	8.16	0.00	0.00
164	C:SER 164	76.70	0.00	0.00
165	C:PHE 165	2.49	0.00	0.00
166	C:THR 166	34.91	0.00	0.00
167	C:VAL 167	75.46	0.00	0.00
168	C:SER 168	102.15	0.00	0.00
169	C:SER 169	41.07	0.00	0.00
170	C:GLU 170	127.31	0.00	0.00
171	C:LYS 171	128.13	0.00	0.00
172	C:THR 172	48.75	0.00	0.00
173	C:ILE 173	100.28	0.00	0.00
174	C:LEU 174	17.98	0.00	0.00
175	C:THR 175	85.12	0.00	0.00
176	C:MET 176	9.50	0.00	0.00
177	C:GLY 177	60.59	0.00	0.00
178	C:GLU 178	114.47	0.00	0.00
179	C:TYR 179	21.75	0.00	0.00
180	C:GLY 180	11.21	0.00	0.00
181	C:ASP 181	45.69	0.00	0.00
182	C:VAL 182	1.96	0.00	0.00
183	C:SER 183	25.39	0.00	0.00
184	C:LEU 184	1.80	0.00	0.00
185	C:LEU 185	69.53	0.00	0.00
186	C:CYS 186	8.84	0.00	0.00
187	C:ARG 187	125.57	0.00	0.00
188	C:VAL 188	23.39	0.00	0.00
189	C:ALA 189	74.81	0.00	0.00
190	C:SER 190	28.61	0.00	0.00
191	C:GLY 191	14.11	0.00	0.00
192	C:VAL 192	27.81	0.00	0.00
193	C:ASP 193	87.85	0.00	0.00
194	C:LEU 194	23.57	0.00	0.00
195	C:ALA 195	67.56	0.00	0.00
196	C:GLN 196	67.55	0.00	0.00
197	C:THR 197	17.52	0.00	0.00
198	C:VAL 198	5.86	0.00	0.00
199	C:ILE 199	2.51	0.00	0.00
200	C:LEU 200	0.51	0.00	0.00
201	C:GLU 201	27.55	0.00	0.00
202	C:LEU 202	22.83	0.00	0.00
203	C:ASP 203	36.36	0.00	0.00
204	C:LYS 204	105.02	0.00	0.00
205	C:THR 205	105.48	0.00	0.00
206	C:VAL 206	65.05	0.00	0.00
207	C:GLU 207	142.94	0.00	0.00
208	C:HIS 208	168.31	0.00	0.00
209	C:LEU 209	71.14	0.00	0.00
210	C:PRO 210	52.54	0.00	0.00
211	C:THR 211	36.45	0.00	0.00
212	C:ALA 212	0.00	0.00	0.00

213	C:TRP 213	17.36	0.00	0.00
214	C:GLN 214	24.73	0.00	0.00
215	C:VAL 215	7.45	0.00	0.00
216	C:HIS 216	87.91	0.00	0.00
217	C:ARG 217	87.65	0.00	0.00
218	C:ASP 218	81.55	0.00	0.00
219	C:TRP 219	88.51	0.00	0.00
220	C:PHE 220	3.28	0.00	0.00
221	C:ASN 221	69.35	0.00	0.00
222	C:ASP 222	113.95	0.00	0.00
223	C:LEU 223	23.90	0.00	0.00
224	C:ALA 224	68.31	0.00	0.00
225	C:LEU 225	19.86	0.00	0.00
226	C:PRO 226	3.66	0.00	0.00
227	C:TRP 227	64.79	0.00	0.00
228	C:LYS 228	23.05	0.00	0.00
229	C:HIS 229	86.16	0.00	0.00
230	C:GLU 230	131.18	0.00	0.00
231	C:GLY 231	77.82	0.00	0.00
232	C:ALA 232	48.84	0.00	0.00
233	C:GLN 233	152.38	0.00	0.00
234	C:ASN 234	76.46	0.00	0.00
235	C:TRP 235	45.28	0.00	0.00
236	C:ASN 236	61.07	0.00	0.00
237	C:ASN 237	67.82	0.00	0.00
238	C:ALA 238	15.88	0.00	0.00
239	C:GLU 239	111.31	0.00	0.00
240	C:ARG 240	113.58	0.00	0.00
241	C:LEU 241	6.06	0.00	0.00
242	C:VAL 242	4.61	0.00	0.00
243	C:GLU 243	85.09	0.00	0.00
244	C:PHE 244	23.04	0.00	0.00
245	C:GLY 245	19.82	0.00	0.00
246	C:ALA 246	92.00	0.00	0.00
247	C:PRO 247	34.98	0.00	0.00
248	C:HIS 248	117.55	0.00	0.00
249	C:ALA 249	22.31	0.00	0.00
250	C:VAL 250	68.91	0.00	0.00
251	C:LYS 251	127.07	0.00	0.00
252	C:MET 252	19.93	0.00	0.00
253	C:ASP 253	67.40	0.00	0.00
254	C:VAL 254	40.04	0.00	0.00
255	C:TYR 255	113.32	0.00	0.00
256	C:ASN 256	67.09	0.00	0.00
257	C:LEU 257	104.87	0.00	0.00
258	C:GLY 258	19.80	0.00	0.00
259	C:ASP 259	73.79	0.00	0.00
260	C:GLN 260	36.21	0.00	0.00
261	C:THR 261	34.95	0.00	0.00
262	C:GLY 262	49.82	0.00	0.00
263	C:VAL 263	89.73	0.00	0.00
264	C:LEU 264	10.04	0.00	0.00
265	C:LEU 265	57.96	0.00	0.00
266	C:LYS 266	156.71	0.00	0.00
267	C:ALA 267	59.76	0.00	0.00
268	C:LEU 268	8.91	0.00	0.00
269	C:ALA 269	88.85	0.00	0.00
270	C:GLY 270	81.91	0.00	0.00
271	C:VAL 271	48.16	0.00	0.00
272	C:PRO 272	64.23	0.00	0.00
273	C:VAL 273	74.99	0.00	0.00
274	C:ALA 274	3.12	0.00	0.00
275	C:HIS 275	69.54	0.00	0.00
276	C:ILE 276	14.78	0.00	0.00
277	C:GLU 277	98.84	0.00	0.00
278	C:GLY 278	65.88	0.00	0.00
279	C:THR 279	69.43	0.00	0.00
280	C:LYS 280	71.79	0.00	0.00
281	C:TYR 281	19.37	0.00	0.00

282	C:HIS 282	23.38	0.00	0.00
283	C:LEU 283	1.97	0.00	0.00
284	C:LYS 284	109.81	0.00	0.00
285	C:SER 285	49.18	0.00	0.00
286	C:GLY 286	16.84	0.00	0.00
287	C:HIS 287	27.22	0.00	0.00
288	C:VAL 288	1.68	0.00	0.00
289	C:THR 289	4.50	0.00	0.00
290	C:CYS 290	2.16	0.00	0.00
291	C:GLU 291	74.55	0.00	0.00
292	C:VAL 292	2.50	0.00	0.00
293	C:GLY 293	14.29	0.00	0.00
294	C:LEU 294	11.78	0.00	0.00
295	C:GLU 295	92.54	0.00	0.00
296	C:LYS 296	167.24	0.00	0.00
297	C:LEU 297	9.49	0.00	0.00
298	C:LYS 298	124.88	0.00	0.00
299	C:MET 299	52.35	0.00	0.00
300	C:LYS 300	45.49	0.00	0.00
301	C:GLY 301	15.87	0.00	0.00
302	C:LEU 302	108.95	0.00	0.00
303	C:THR 303	135.04	0.00	0.00
304	C:TYR 304	71.88	0.00	0.00
305	C:THR 305	99.62	0.00	0.00
306	C:MET 306	110.71	0.00	0.00
307	C:CYS 307	13.66	0.00	0.00
308	C:ASP 308	64.31	0.00	0.00
309	C:LYS 309	116.23	0.00	0.00
310	C:THR 310	84.41	0.00	0.00
311	C:LYS 311	93.37	0.00	0.00
312	C:PHE 312	15.13	0.00	0.00
313	C:THR 313	68.55	0.00	0.00
314	C:TRP 314	55.55	0.00	0.00
315	C:LYS 315	136.35	0.00	0.00
316	C:ARG 316	161.64	0.00	0.00
317	C:ALA 317	53.45	0.00	0.00
318	C:PRO 318	11.04	0.00	0.00
319	C:THR 319	77.83	0.00	0.00
320	C:ASP 320	81.33	0.00	0.00
321	C:SER 321	18.45	0.00	0.00
322	C:GLY 322	75.56	0.00	0.00
323	C:HIS 323	38.70	0.00	0.00
324	C:ASP 324	46.35	0.00	0.00
325	C:THR 325	3.03	0.00	0.00
326	C:VAL 326	1.01	0.00	0.00
327	C:VAL 327	25.41	0.00	0.00
328	C:MET 328	1.96	0.00	0.00
329	C:GLU 329	30.06	0.00	0.00
330	C:VAL 330	2.66	0.00	0.00
331	C:THR 331	55.87	0.00	0.00
332	C:PHE 332	7.65	0.00	0.00
333	C:SER 333	78.00	0.00	0.00
334	C:GLY 334	20.44	0.00	0.00
335	C:THR 335	119.67	0.00	0.00
336	C:LYS 336	83.73	0.00	0.00
337	C:PRO 337	57.90	0.00	0.00
338	C:CYS 338	2.51	0.00	0.00
339	C:ARG 339	67.72	0.00	0.00
340	C:ILE 340	13.20	0.00	0.00
341	C:PRO 341	28.55	0.00	0.00
342	C:VAL 342	22.03	0.00	0.00
343	C:ARG 343	118.13	0.00	0.00
344	C:ALA 344	1.05	0.00	0.00
345	C:VAL 345	15.06	0.00	0.00
346	C:ALA 346	34.04	0.00	0.00
347	C:HIS 347	118.04	0.00	0.00
348	C:GLY 348	77.71	0.00	0.00
349	C:SER 349	52.77	0.00	0.00
350	C:PRO 350	121.49	0.00	0.00

<table>
<tr><td>351</td><td>C:ASP 351</td><td>115.55</td><td>0.00</td><td></td><td>0.00</td></tr>
<tr><td>352</td><td>C:VAL 352</td><td>99.12</td><td>0.00</td><td></td><td>0.00</td></tr>
<tr><td>353</td><td>C:ASN 353</td><td>92.20</td><td>0.00</td><td></td><td>0.00</td></tr>
<tr><td>354</td><td>C:VAL 354</td><td>37.86</td><td>0.00</td><td></td><td>0.00</td></tr>
<tr><td>355</td><td>C:ALA 355</td><td>14.23</td><td>0.00</td><td></td><td>0.00</td></tr>
<tr><td>356</td><td>C:MET 356</td><td>125.22</td><td>0.00</td><td></td><td>0.00</td></tr>
<tr><td>357</td><td>C:LEU 357</td><td>65.02</td><td>0.00</td><td></td><td>0.00</td></tr>
<tr><td>358</td><td>C:ILE 358</td><td>21.06</td><td>0.00</td><td></td><td>0.00</td></tr>
<tr><td>359</td><td>C:THR 359</td><td>9.45</td><td>0.00</td><td></td><td>0.00</td></tr>
<tr><td>360</td><td>C:PRO 360</td><td>50.04</td><td>0.00</td><td></td><td>0.00</td></tr>
<tr><td>361</td><td>C:ASN 361</td><td>49.59</td><td>0.00</td><td></td><td>0.00</td></tr>
<tr><td>362</td><td>C:PRO 362</td><td>16.05</td><td>0.00</td><td></td><td>0.00</td></tr>
<tr><td>363</td><td>C:THR 363</td><td>28.50</td><td>0.00</td><td></td><td>0.00</td></tr>
<tr><td>364</td><td>C:ILE 364</td><td>6.38</td><td>0.00</td><td></td><td>0.00</td></tr>
<tr><td>365</td><td>C:GLU 365</td><td>47.42</td><td>0.00</td><td></td><td>0.00</td></tr>
<tr><td>366</td><td>C:ASN 366</td><td>118.66</td><td>0.00</td><td></td><td>0.00</td></tr>
<tr><td>367</td><td>C:ASN 367</td><td>139.30</td><td>0.00</td><td></td><td>0.00</td></tr>
<tr><td>368</td><td>C:GLY 368</td><td>35.01</td><td>0.00</td><td></td><td>0.00</td></tr>
<tr><td>369</td><td>C:GLY 369</td><td>36.90</td><td>0.00</td><td></td><td>0.00</td></tr>
<tr><td>370</td><td>C:GLY 370</td><td>4.35</td><td>0.00</td><td></td><td>0.00</td></tr>
<tr><td>371</td><td>C:PHE 371</td><td>13.14</td><td>0.00</td><td></td><td>0.00</td></tr>
<tr><td>372</td><td>C:ILE 372</td><td>0.49</td><td>0.00</td><td></td><td>0.00</td></tr>
<tr><td>373</td><td>C:GLU 373</td><td>0.83</td><td>0.00</td><td></td><td>0.00</td></tr>
<tr><td>374</td><td>C:MET 374</td><td>0.00</td><td>0.00</td><td></td><td>0.00</td></tr>
<tr><td>375</td><td>C:GLN 375</td><td>59.93</td><td>0.00</td><td></td><td>0.00</td></tr>
<tr><td>376</td><td>C:LEU 376</td><td>5.25</td><td>0.00</td><td></td><td>0.00</td></tr>
<tr><td>377</td><td>C:PRO 377</td><td>46.71</td><td>0.00</td><td></td><td>0.00</td></tr>
<tr><td>378</td><td>C:PRO 378</td><td>53.28</td><td>0.00</td><td></td><td>0.00</td></tr>
<tr><td>379</td><td>C:GLY 379</td><td>23.97</td><td>0.00</td><td></td><td>0.00</td></tr>
<tr><td>380</td><td>C:ASP 380</td><td>66.12</td><td>0.00</td><td></td><td>0.00</td></tr>
<tr><td>381</td><td>C:ASN 381</td><td>2.04</td><td>0.00</td><td></td><td>0.00</td></tr>
<tr><td>382</td><td>C:ILE 382</td><td>33.67</td><td>0.00</td><td></td><td>0.00</td></tr>
<tr><td>383</td><td>C:ILE 383</td><td>0.12</td><td>0.00</td><td></td><td>0.00</td></tr>
<tr><td>384</td><td>C:TYR 384</td><td>55.02</td><td>0.00</td><td></td><td>0.00</td></tr>
<tr><td>385</td><td>C:VAL 385</td><td>0.15</td><td>0.00</td><td></td><td>0.00</td></tr>
<tr><td>386</td><td>C:GLY 386</td><td>21.00</td><td>0.00</td><td></td><td>0.00</td></tr>
<tr><td>387</td><td>C:GLU 387</td><td>113.88</td><td>0.00</td><td></td><td>0.00</td></tr>
<tr><td>388</td><td>C:LEU 388</td><td>39.42</td><td>0.00</td><td></td><td>0.00</td></tr>
<tr><td>389</td><td>C:SER 389</td><td>51.25</td><td>0.00</td><td></td><td>0.00</td></tr>
<tr><td>390</td><td>C:HIS 390</td><td>59.87</td><td>0.00</td><td></td><td>0.00</td></tr>
<tr><td>391</td><td>C:GLN 391</td><td>106.66</td><td>0.00</td><td></td><td>0.00</td></tr>
<tr><td>392</td><td>C:TRP 392</td><td>38.31</td><td>0.00</td><td></td><td>0.00</td></tr>
<tr><td>393</td><td>C:PHE 393</td><td>136.80</td><td>0.00</td><td></td><td>0.00</td></tr>
<tr><td>394</td><td>C:GLN 394</td><td>12.91</td><td>0.00</td><td></td><td>0.00</td></tr>
<tr><td>395</td><td>C:LYS 395</td><td>152.01</td><td>0.00</td><td></td><td>0.00</td></tr>
<tr><td>396</td><td>C:GLY 396</td><td>65.42</td><td>0.00</td><td></td><td>0.00</td></tr>
<tr><td>397</td><td>C:SER 397</td><td>51.66</td><td>0.00</td><td></td><td>0.00</td></tr>
<tr><td>398</td><td>C:SER 398</td><td>72.56</td><td>0.00</td><td></td><td>0.00</td></tr>
<tr><td>399</td><td>C:ILE 399</td><td>141.96</td><td>0.00</td><td></td><td>0.00</td></tr>
<tr><td>400</td><td>C:GLY 400</td><td>32.78</td><td>0.00</td><td></td><td>0.00</td></tr>
<tr><td>401</td><td>C:ARG 401</td><td>54.48</td><td>0.00</td><td></td><td>0.00</td></tr>
<tr><td>402</td><td>C:VAL 402</td><td>101.34</td><td>0.00</td><td></td><td>0.00</td></tr>
<tr><td>403</td><td>C:PHE 403</td><td>126.89</td><td>0.00</td><td></td><td>0.00</td></tr>
<tr><td>404</td><td>C:GLN 404</td><td>112.30</td><td>0.00</td><td></td><td>0.00</td></tr>
<tr><td>405</td><td>C:LYS 405</td><td>152.17</td><td>0.00</td><td></td><td>0.00</td></tr>
<tr><td>406</td><td>C:THR 406</td><td>74.93</td><td>0.00</td><td></td><td>0.00</td></tr>
<tr><td>407</td><td>C:LYS 407</td><td>117.54</td><td>0.00</td><td></td><td>0.00</td></tr>
<tr><td>408</td><td>C:LYS 408</td><td>69.45</td><td>0.00</td><td></td><td>0.00</td></tr>
<tr><td>409</td><td>C:GLY 409</td><td>30.14</td><td>0.00</td><td></td><td>0.00</td></tr>
<tr><td>410</td><td>C:ILE 410</td><td>105.16</td><td>18.59</td><td>||</td><td>0.30</td></tr>
<tr><td>411</td><td>C:GLU 411</td><td>109.48</td><td>0.00</td><td></td><td>0.00</td></tr>
<tr><td>412</td><td>C:ARG 412</td><td>45.73</td><td>0.00</td><td></td><td>0.00</td></tr>
<tr><td>413</td><td>C:LEU 413</td><td>55.54</td><td>36.89</td><td>|||||||</td><td>0.56</td></tr>
<tr><td>414</td><td>C:THR 414</td><td>65.22</td><td>34.96</td><td>||||||</td><td>-0.12</td></tr>
<tr><td>415</td><td>C:VAL 415</td><td>108.39</td><td>14.36</td><td>||</td><td>-0.16</td></tr>
<tr><td>416</td><td>C:ILE 416</td><td>20.10</td><td>8.51</td><td>|||||</td><td>-0.03</td></tr>
<tr><td>417</td><td>C:GLY 417</td><td>19.39</td><td>19.39</td><td>||||||||||</td><td>0.31</td></tr>
<tr><td>418</td><td>C:GLU 418</td><td>62.99</td><td>0.87</td><td>|</td><td>-0.01</td></tr>
<tr><td>419</td><td>C:HIS 419</td><td>43.24</td><td>0.00</td><td></td><td>0.00</td></tr>
</table>

420	C:ALA 420	18.12	0.00		0.00
421	C:TRP 421	80.05	16.06	|||	0.26
422	C:ASP 422	19.42	0.00		0.00
423	C:PHE 423	37.05	0.00		0.00
424	C:GLY 424	35.06	0.00		0.00
425	C:SER 425	22.09	0.00		0.00
426	C:ALA 426	97.96	0.00		0.00
427	C:GLY 427	56.18	0.00		0.00
428	C:GLY 428	51.37	0.00		0.00
429	C:PHE 429	173.97	0.00		0.00
430	C:LEU 430	144.44	0.00		0.00
431	C:SER 431	23.85	0.00		0.00
432	C:SER 432	67.42	0.00		0.00
433	C:ILE 433	97.56	0.00		0.00
434	C:GLY 434	11.30	4.46	||||	0.05
435	C:LYS 435	79.81	0.00		0.00
436	C:ALA 436	55.76	0.00		0.00
437	C:VAL 437	75.09	13.39	||	0.21
438	C:HIS 438	54.99	53.49	|||||||||	0.91
439	C:THR 439	63.28	0.00		0.00
440	C:VAL 440	99.94	0.00		0.00
441	C:LEU 441	112.27	10.05			0.16
442	C:GLY 442	25.14	14.22	|||||	0.18
443	C:GLY 443	38.43	0.00		0.00
444	C:ALA 444	55.78	0.00		0.00
445	C:PHE 445	50.20	3.14			0.05
446	C:ASN 446	97.77	9.39			-0.09
447	C:SER 447	97.70	0.00		0.00
448	C:ILE 448	125.16	0.00		0.00
449	C:PHE 449	31.61	0.00		0.00
450	C:GLY 450	44.24	0.00		0.00
451	C:GLY 451	91.86	0.00		0.00
452	C:VAL 452	60.33	0.00		0.00
453	C:GLY 453	37.75	0.00		0.00
454	C:PHE 454	151.43	0.00		0.00
455	C:LEU 455	106.10	0.00		0.00
456	C:PRO 456	58.09	0.00		0.00
457	C:LYS 457	35.80	0.00		0.00
458	C:LEU 458	83.72	0.00		0.00
459	C:LEU 459	111.54	0.00		0.00
460	C:LEU 460	85.99	0.00		0.00
461	C:GLY 461	0.24	0.00		0.00
462	C:VAL 462	74.47	0.00		0.00
463	C:ALA 463	46.95	0.00		0.00
464	C:LEU 464	51.21	0.00		0.00
465	C:ALA 465	25.77	0.00		0.00
466	C:TRP 466	163.18	0.00		0.00
467	C:LEU 467	81.75	0.00		0.00
468	C:GLY 468	1.79	0.00		0.00
469	C:LEU 469	129.47	0.00		0.00
470	C:ASN 470	93.08	0.00		0.00
471	C:MET 471	49.84	0.00		0.00
472	C:ARG 472	204.38	0.00		0.00
473	C:ASN 473	149.43	0.00		0.00
474	C:PRO 474	64.87	0.00		0.00
475	C:THR 475	119.08	0.00		0.00
476	C:MET 476	111.99	0.00		0.00
477	C:SER 477	25.89	0.00		0.00
478	C:MET 478	126.87	0.00		0.00
479	C:SER 479	63.70	0.00		0.00
480	C:PHE 480	60.04	0.00		0.00
481	C:LEU 481	68.06	0.00		0.00
482	C:LEU 482	92.54	0.00		0.00
483	C:ALA 483	46.83	0.00		0.00
484	C:GLY 484	0.16	0.00		0.00
485	C:GLY 485	34.52	0.00		0.00
486	C:LEU 486	107.71	0.00		0.00
487	C:VAL 487	9.62	0.00		0.00
488	C:LEU 488	53.04	0.00		0.00

489	C:ALA 489	57.92	0.00	0.00
490	C:MET 490	97.66	43.08 |||||	0.69
491	C:THR 491	27.49	0.00	0.00
492	C:LEU 492	140.64	0.00	0.00
493	C:GLY 493	67.87	10.98 ||	0.11
494	C:VAL 494	137.40	22.59 ||	0.36

PDBe PISA v1.52 [20/10/2014]

PDBe Protein Data Bank in Europe

is a member of

PDB

EMDataBank Unified Data Resource for 3DEM

This website uses cookies. By continuing to browse this site, you are agreeing to the use of our site cookies. To find out more, see our Terms of Use.

OK

PISA Interface.

Session Map (id=179-P6-IE2)
Start | Interfaces | Interface Search
Monomers
Assemblies

interface # 56 in ourmodelfortest2.pdb crystal.

Space symmetry group: P 1

interface #56/96 XML << < > >>

Interface Summary XML

	Structure 1		Structure 2	
**Selection range**	[CPL]A:500		E	
**class**	Ligand		Protein	
**symmetry operation**	x,y,z		x,y,z	
**symmetry ID**	1_555		0_555	
**Number of atoms**				
**interface**	3	10.7%	9	1.6%
**surface**	27	96.4%	458	79.0%
**total**	28	100.0%	580	100.0%
**Number of residues**				
**interface**	1	100.0%	4	5.4%
**surface**	1	100.0%	74	100.0%
**total**	1	100.0%	74	100.0%
**Solvent-accessible area, Å**				
**interface**	65.2	9.6%	56.4	0.8%
**total**	676.9	100.0%	7155.8	100.0%
**Solvation energy, kcal/mol**				
**isolated structure**	3.0	100.0%	-48.5	100.0%
**gain on complex formation**	2.2	71.1%	0.1	-0.1%
**average gain**	-0.3	-11.1%	-1.0	2.0%
**P-value**	0.937		0.839	

View structure 1 | interface | structure 2

Download
structure 1 | interface | structure 2

This interface scored

0.000

in Complex Formation Significance Score (CSS).
CSS ranges from 0 to 1 as interface relevance to complex formation increases.

Achieved CSS implies that the interface does not play any role in complex formation and seems to be a result of

No disulfide bonds found
No covalent bonds found
No hydrogen bonds found
No salt bridges found

Interfacing residues (not a contact table) XML Display level: Residues

Inaccessible residues HSDC Residues making **H**ydrogen/**D**isulphide bond, **S**alt bridge or **C**ovalent link
Solvent-accessible residues Interfacing residues
ASA Accessible Surface Area, Å² **BSA** Buried Surface Area, Å² **Δ^iG** Solvation energy effect, kcal/mol |||| Buried area percentage, one bar per 10%

##	Structure 1	HSDC	ASA	BSA	Δ^iG	##	Structure 2	HSDC	ASA	BSA	Δ^iG
1	A:CPL 500		676.90	65.22 |	-2.15	1	E:SER 1		162.28	0.00	0.00
						2	E:VAL 2		137.23	0.00	0.00
						3	E:LEU 3		161.24	0.00	0.00
						4	E:ILE 4		147.13	0.00	0.00
						5	E:PRO 5		112.52	0.00	0.00
						6	E:SER 6		110.25	0.00	0.00
						7	E:HIS 7		135.91	0.00	0.00
						8	E:ALA 8		56.90	0.00	0.00
						9	E:GLN 9		111.22	0.00	0.00
						10	E:GLY 10		66.09	13.25 |||	-0.15
						11	E:GLU 11		144.11	12.74 |	-0.06
						12	E:LEU 12		131.89	0.17 |	0.00
						13	E:THR 13		123.08	30.28 |||	0.14
						14	E:GLY 14		54.55	0.00	0.00
						15	E:ARG 15		245.38	0.00	0.00
						16	E:GLY 16		60.33	0.00	0.00
						17	E:HIS 17		196.30	0.00	0.00
						18	E:LYS 18		154.71	0.00	0.00
						19	E:TRP 19		218.21	0.00	0.00
						20	E:LEU 20		127.06	0.00	0.00
						21	E:GLU 21		159.70	0.00	0.00
						22	E:GLY 22		35.97	0.00	0.00
						23	E:ASP 23		111.68	0.00	0.00
						24	E:SER 24		28.80	0.00	0.00
						25	E:LEU 25		125.66	0.00	0.00
						26	E:ARG 26		170.66	0.00	0.00
						27	E:THR 27		71.61	0.00	0.00
						28	E:HIS 28		58.67	0.00	0.00
						29	E:LEU 29		99.01	0.00	0.00
						30	E:THR 30		91.14	0.00	0.00
						31	E:ARG 31		141.69	0.00	0.00
						32	E:VAL 32		15.71	0.00	0.00
						33	E:GLU 33		100.81	0.00	0.00
						34	E:GLY 34		44.02	0.00	0.00
						35	E:TRP 35		57.77	0.00	0.00
						36	E:VAL 36		40.10	0.00	0.00
						37	E:TRP 37		191.72	0.00	0.00
						38	E:LYS 38		167.61	0.00	0.00
						39	E:ASN 39		50.19	0.00	0.00
						40	E:LYS 40		102.83	0.00	0.00
						41	E:LEU 41		141.60	0.00	0.00
						42	E:LEU 42		95.36	0.00	0.00
						43	E:ALA 43		11.07	0.00	0.00
						44	E:LEU 44		112.31	0.00	0.00
						45	E:ALA 45		44.53	0.00	0.00
						46	E:MET 46		31.31	0.00	0.00
						47	E:VAL 47		52.53	0.00	0.00
						48	E:THR 48		74.21	0.00	0.00
						49	E:VAL 49		86.49	0.00	0.00
						50	E:VAL 50		11.06	0.00	0.00
						51	E:TRP 51		162.60	0.00	0.00
						52	E:LEU 52		136.64	0.00	0.00
						53	E:THR 53		85.79	0.00	0.00
						54	E:LEU 54		80.44	0.00	0.00
						55	E:GLU 55		165.15	0.00	0.00
						56	E:SER 56		45.11	0.00	0.00
						57	E:VAL 57		100.08	0.00	0.00
						58	E:VAL 58		112.68	0.00	0.00
						59	E:THR 59		58.47	0.00	0.00
						60	E:ARG 60		33.88	0.00	0.00
						61	E:VAL 61		78.68	0.00	0.00
						62	E:ALA 62		53.34	0.00	0.00
						63	E:VAL 63		58.74	0.00	0.00
						64	E:LEU 64		34.04	0.00	0.00
						65	E:VAL 65		78.01	0.00	0.00
						66	E:VAL 66		77.68	0.00	0.00
						67	E:LEU 67		65.20	0.00	0.00
						68	E:LEU 68		60.55	0.00	0.00
						69	E:CYS 69		47.98	0.00	0.00
						70	E:LEU 70		100.40	0.00	0.00
						71	E:ALA 71		28.11	0.00	0.00
						72	E:PRO 72		15.62	0.00	0.00
						73	E:VAL 73		75.89	0.00	0.00
						74	E:TYR 74		218.50	0.00	0.00

This website uses cookies. By continuing to browse this site, you are agreeing to the use of our site cookies. To find out more, see our Terms of Use.

OK

Protein Data Bank in Europe

PDBePISA

pdbe.org/pisa

Bringing Structure to Biology

PISA Interface.

interface # 57 in ourmodelfortest2.pdb crystal.

Space symmetry group: P 1

interface #57/96

XML << < > >>

Interface Summary

XML

	Structure 1		Structure 2	
**Selection range**	[CPL]B:500		F	
**class**	Ligand		Protein	
**symmetry operation**	x,y,z		x,y,z	
**symmetry ID**	1_555		0_555	
**Number of atoms**				
**interface**	3	10.7%	7	1.2%
**surface**	27	96.4%	440	77.5%
**total**	28	100.0%	568	100.0%
**Number of residues**				
**interface**	1	100.0%	4	5.5%
**surface**	1	100.0%	73	100.0%
**total**	1	100.0%	73	100.0%
**Solvent-accessible area, Å**				
**interface**	61.0	9.2%	56.6	0.8%
**total**	660.8	100.0%	6964.6	100.0%
**Solvation energy, kcal/mol**				
**isolated structure**	2.3	100.0%	-42.8	100.0%
**gain on complex formation**	2.0	86.3%	-0.2	0.4%
**average gain**	-0.3	-11.1%	-0.8	2.0%
**P-value**	0.936		0.773	

View structure 1 | interface | structure 2

Download structure 1 | interface | structure 2

This interface scored

0.000

in Complex Formation Significance Score (CSS).

CSS ranges from 0 to 1 as interface relevance to complex formation increases.

Achieved CSS implies that the interface does not play any role in complex formation and seems to be a result of

No disulfide bonds found

No covalent bonds found

No hydrogen bonds found

No salt bridges found

Interfacing residues (not a contact table) XML Display level: Residues

Inaccessible residues HSDC Residues making **H**ydrogen/**D**isulphide bond, **S**alt bridge or **C**ovalent link

Solvent-accessible residues Interfacing residues

ASA Accessible Surface Area, Å² **BSA** Buried Surface Area, Å² **ΔⁱG** Solvation energy effect, kcal/mol |||| Buried area percentage, one bar per 10%

##	Structure 1	HSDC	ASA	BSA	Δ^i^G	##	Structure 2	HSDC	ASA	BSA	Δ^i^G
1	B:CPL 500		660.81	60.99 |	-2.01	1	F:SER 1		171.32	0.00	0.00
						2	F:VAL 2		141.48	0.00	0.00
						3	F:LEU 3		150.11	0.00	0.00
						4	F:ILE 4		158.00	0.00	0.00
						5	F:PRO 5		100.01	0.00	0.00
						6	F:SER 6		109.34	0.00	0.00
						7	F:HIS 7		142.17	0.00	0.00
						8	F:ALA 8		58.49	0.00	0.00
						9	F:GLN 9		104.15	0.00	0.00
						10	F:GLY 10		68.14	17.90 |||	-0.20
						11	F:GLU 11		142.29	10.38 |	-0.08
						12	F:LEU 12		144.81	0.50 |	0.01
						13	F:THR 13		119.10	27.83 |||	0.44
						14	F:GLY 14		51.05	0.00	0.00
						15	F:ARG 15		244.79	0.00	0.00
						16	F:GLY 16		63.06	0.00	0.00
						17	F:HIS 17		195.74	0.00	0.00
						18	F:LYS 18		139.73	0.00	0.00
						19	F:TRP 19		221.87	0.00	0.00
						20	F:LEU 20		129.13	0.00	0.00
						21	F:GLU 21		121.55	0.00	0.00
						22	F:GLY 22		24.62	0.00	0.00
						23	F:ASP 23		100.64	0.00	0.00
						24	F:SER 24		35.09	0.00	0.00
						25	F:LEU 25		137.96	0.00	0.00
						26	F:ARG 26		161.11	0.00	0.00
						27	F:THR 27		30.15	0.00	0.00
						28	F:HIS 28		74.34	0.00	0.00
						29	F:LEU 29		113.18	0.00	0.00
						30	F:THR 30		90.96	0.00	0.00
						31	F:ARG 31		140.07	0.00	0.00
						32	F:VAL 32		26.26	0.00	0.00
						33	F:GLU 33		90.06	0.00	0.00
						34	F:GLY 34		36.46	0.00	0.00
						35	F:TRP 35		64.77	0.00	0.00
						36	F:VAL 36		37.48	0.00	0.00
						37	F:TRP 37		174.62	0.00	0.00
						38	F:LYS 38		141.86	0.00	0.00
						39	F:ASN 39		28.89	0.00	0.00
						40	F:LYS 40		116.81	0.00	0.00
						41	F:LEU 41		150.44	0.00	0.00
						42	F:LEU 42		87.34	0.00	0.00
						43	F:ALA 43		4.32	0.00	0.00
						44	F:LEU 44		106.33	0.00	0.00
						45	F:ALA 45		32.82	0.00	0.00
						46	F:MET 46		94.80	0.00	0.00
						47	F:VAL 47		47.85	0.00	0.00
						48	F:THR 48		78.21	0.00	0.00
						49	F:VAL 49		79.37	0.00	0.00
						50	F:VAL 50		21.09	0.00	0.00
						51	F:TRP 51		135.03	0.00	0.00
						52	F:LEU 52		155.33	0.00	0.00
						53	F:THR 53		101.07	0.00	0.00
						54	F:LEU 54		44.71	0.00	0.00
						55	F:GLU 55		144.82	0.00	0.00
						56	F:SER 56		51.76	0.00	0.00
						57	F:VAL 57		86.74	0.00	0.00
						58	F:VAL 58		109.01	0.00	0.00
						59	F:THR 59		55.44	0.00	0.00
						60	F:ARG 60		33.48	0.00	0.00
						61	F:VAL 61		75.74	0.00	0.00
						62	F:ALA 62		52.99	0.00	0.00
						63	F:VAL 63		55.55	0.00	0.00
						64	F:LEU 64		25.28	0.00	0.00
						65	F:VAL 65		77.55	0.00	0.00
						66	F:VAL 66		80.18	0.00	0.00
						67	F:LEU 67		81.69	0.00	0.00
						68	F:LEU 68		63.56	0.00	0.00
						69	F:CYS 69		58.15	0.00	0.00
						70	F:LEU 70		119.83	0.00	0.00
						71	F:ALA 71		27.06	0.00	0.00
						72	F:PRO 72		45.84	0.00	0.00
						73	F:VAL 73		179.54	0.00	0.00

PDBe
Protein Data Bank in Europe

is a member of

wwPDB

EMDataBank
Unified Data Resource for 3DEM

This website uses cookies. By continuing to browse this site, you are agreeing to the use of our site cookies. To find out more, see our Terms of Use.

OK

Protein Data Bank in Europe

PDBePISA

pdbe.org/pisa

Bringing Structure to Biology

PISA Interface.

interface # 53 in ourmodelfortest2.pdb crystal.

Space symmetry group: P 1

interface #53/96 XML << < > >>

Interface Summary XML

	Structure 1		Structure 2	
**Selection range**	[CPL]C:500		J	
**class**	Ligand		Protein	
**symmetry operation**	x,y,z		x,y,z	
**symmetry ID**	1_555		0_555	
**Number of atoms**				
**interface**	3	10.7%	8	1.4%
**surface**	27	96.4%	449	77.4%
**total**	28	100.0%	580	100.0%
**Number of residues**				
**interface**	1	100.0%	4	5.4%
**surface**	1	100.0%	74	100.0%
**total**	1	100.0%	74	100.0%
**Solvent-accessible area, Å**				
**interface**	66.4	10.0%	59.7	0.8%
**total**	661.8	100.0%	7152.1	100.0%
**Solvation energy, kcal/mol**				
**isolated structure**	1.6	100.0%	-45.8	100.0%
**gain on complex formation**	2.2	138.4%	0.1	-0.3%
**average gain**	-0.2	-11.1%	-0.9	2.0%
**P-value**	0.943		0.855	

View structure 1 interface structure 2

Download
structure 1 interface structure 2

This interface scored

0.000

in Complex Formation Significance Score (CSS).
CSS ranges from 0 to 1 as interface relevance to complex formation increases.

Achieved CSS implies that the interface does not play any role in complex formation and seems to be a result of

No disulfide bonds found
No covalent bonds found
No hydrogen bonds found
No salt bridges found

Interfacing residues (not a contact table) XML Display level: Residues

Inaccessible residues HSDC Residues making **H**ydrogen/**D**isulphide bond, **S**alt bridge or **C**ovalent link
Solvent-accessible residues Interfacing residues
ASA Accessible Surface Area, Å² **BSA** Buried Surface Area, Å² **ΔⁱG** Solvation energy effect, kcal/mol |||| Buried area percentage, one bar per 10%

##	Structure 1	HSDC	ASA	BSA	Δ^iG	##	Structure 2	HSDC	ASA	BSA	Δ^iG
1	C:CPL 500		661.85	66.41 ‖	-2.19	1	J:SER 1		162.75	0.00	0.00
						2	J:VAL 2		136.01	0.00	0.00
						3	J:LEU 3		156.32	0.00	0.00
						4	J:ILE 4		146.95	0.00	0.00
						5	J:PRO 5		112.87	0.00	0.00
						6	J:SER 6		111.93	0.00	0.00
						7	J:HIS 7		140.01	0.00	0.00
						8	J:ALA 8		56.50	0.00	0.00
						9	J:GLN 9		101.99	0.00	0.00
						10	J:GLY 10		72.91	13.87 ‖	-0.16
						11	J:GLU 11		137.09	11.39 |	0.00
						12	J:LEU 12		132.55	1.17 |	0.02
						13	J:THR 13		130.61	33.23 |||	-0.01
						14	J:GLY 14		53.78	0.00	0.00
						15	J:ARG 15		245.58	0.00	0.00
						16	J:GLY 16		59.77	0.00	0.00
						17	J:HIS 17		196.27	0.00	0.00
						18	J:LYS 18		136.18	0.00	0.00
						19	J:TRP 19		234.99	0.00	0.00
						20	J:LEU 20		138.57	0.00	0.00
						21	J:GLU 21		165.37	0.00	0.00
						22	J:GLY 22		38.46	0.00	0.00
						23	J:ASP 23		116.00	0.00	0.00
						24	J:SER 24		55.09	0.00	0.00
						25	J:LEU 25		138.00	0.00	0.00
						26	J:ARG 26		185.03	0.00	0.00
						27	J:THR 27		56.10	0.00	0.00
						28	J:HIS 28		62.48	0.00	0.00
						29	J:LEU 29		89.84	0.00	0.00
						30	J:THR 30		87.63	0.00	0.00
						31	J:ARG 31		126.79	0.00	0.00
						32	J:VAL 32		7.19	0.00	0.00
						33	J:GLU 33		96.02	0.00	0.00
						34	J:GLY 34		38.16	0.00	0.00
						35	J:TRP 35		61.93	0.00	0.00
						36	J:VAL 36		33.41	0.00	0.00
						37	J:TRP 37		189.93	0.00	0.00
						38	J:LYS 38		151.65	0.00	0.00
						39	J:ASN 39		45.58	0.00	0.00
						40	J:LYS 40		126.10	0.00	0.00
						41	J:LEU 41		148.20	0.00	0.00
						42	J:LEU 42		82.25	0.00	0.00
						43	J:ALA 43		9.33	0.00	0.00
						44	J:LEU 44		113.85	0.00	0.00
						45	J:ALA 45		32.51	0.00	0.00
						46	J:MET 46		85.27	0.00	0.00
						47	J:VAL 47		54.89	0.00	0.00
						48	J:THR 48		73.48	0.00	0.00
						49	J:VAL 49		87.01	0.00	0.00
						50	J:VAL 50		13.06	0.00	0.00
						51	J:TRP 51		141.12	0.00	0.00
						52	J:LEU 52		149.66	0.00	0.00
						53	J:THR 53		85.62	0.00	0.00
						54	J:LEU 54		74.09	0.00	0.00
						55	J:GLU 55		162.28	0.00	0.00
						56	J:SER 56		42.81	0.00	0.00
						57	J:VAL 57		92.00	0.00	0.00
						58	J:VAL 58		110.62	0.00	0.00
						59	J:THR 59		51.38	0.00	0.00
						60	J:ARG 60		25.25	0.00	0.00
						61	J:VAL 61		78.68	0.00	0.00
						62	J:ALA 62		55.30	0.00	0.00
						63	J:VAL 63		52.85	0.00	0.00
						64	J:LEU 64		28.26	0.00	0.00
						65	J:VAL 65		76.16	0.00	0.00
						66	J:VAL 66		85.68	0.00	0.00
						67	J:LEU 67		83.86	0.00	0.00
						68	J:LEU 68		66.46	0.00	0.00
						69	J:CYS 69		49.65	0.00	0.00
						70	J:LEU 70		81.67	0.00	0.00
						71	J:ALA 71		14.22	0.00	0.00
						72	J:PRO 72		25.51	0.00	0.00
						73	J:VAL 73		86.17	0.00	0.00
						74	J:TYR 74		198.56	0.00	0.00

is a member of
