# Supplementary material for: Molecular Organisation of Tick-Borne Encephalitis Virus
Source: Viruses. 2022 Apr 11;14(4):792. doi: 10.3390/v14040792 (PMC9027435; doi:10.3390/v14040792)
Supplement: Supplementary file 1 [file viruses-14-00792-s001.zip › Supplementary figures captions.pdf]

Figure S1 Biochemical characterization of purified TBEV preparation 1 (**a**) SDS-PAGE analysis of the purified TBEV preparation 1 after UV inactivation. Lane 1 shows the TBEV preparation, lane 2 the molecular size marker. The TBEV protein bands and the sizes of the molecular size marker are indicated. (**b**) Anti-E and anti-M immunoblot analysis of the purified TBEV preparation 1 after UV inactivation. Lane 1 shows the TBEV preparation, lane 2 the molecular size marker. The E and M protein bands and the sizes of the molecular size marker are indicated.

Figure S2 Cryo-EM reconstruction workflow (**a**) Representative cryo-EM micrographs of purified TBEV virions from the three preparations. Intact virions are indicated with black arrows and disturbed virions with white arrows. The length of the scale bars is 100 nm. (**b**) Representative 2D class averages of the three datasets. 2D classification was performed in Relion 3.1 (**c**) Reconstructions of the three separate datasets shown as surface views. The global resolutions of the maps (FSC 0.143 criterion), and the number of particles used in the reconstructions are indicated. 3D classification, refinement, and FSC calculations were performed in Relion 3.1. The maps are shown at 2.9 σ above the mean. (**d**) Final map of the combined reconstruction shown as a surface view. The number of particles used in the reconstruction are indicated. The FSC plot of the combined dataset map is shown with the FSC 0.143 cut-off, and the final resolution indicated. Refinement and FSC calculations were performed in Relion 3.1. The map is shown at 2.9 σ above the mean.

Figure S3 Local resolution of the final map (**a**) Isosurface representation of the final map coloured by local resolution calculated in Relion 3.1. The two, three, and five-fold symmetry axes are indicated by an ellipse, a triangle, and by stars, respectively (**b**) Isosurface representation of a central 60 Å slice of the final map coloured by local resolution calculated in Relion 3.1. The maps are shown at 2.35 σ above the mean.

Figure S4 Comparison of M-H1 densities (**a**) Final locally sharpened map with M-H1 helices of chains F and D of the model shown. Residues Glu33, Thr37, and Lys40 of both chains are shown and labelled. The map is shown at 2.2 σ above the mean. The view is from the outside of the virion towards the interior and the slice thickness is 25 Å. (**b**) Final minimally sharpened (B factor -5 Å^2) map with M-H1 helices of chains F and D of the model shown. Residues Glu33, Thr37, and Lys40 of both chains are shown and labelled. The map is shown at 3.5 σ above the mean. The view and slice thickness is the same as in panel A. (**c**) Preparation 1 minimally sharpened (B factor –5 Å^2). The map is shown at 3.5 σ above the mean. The view and slice thickness is the same as in panel A. (**d**) Previously-published TBEV map (EMDB-3752) and TBEV model (PDB-5o6a) with M-H1 helices of chains F and D of the model shown. Glu33, Thr37, and Arg40 of both chains are shown and labelled. The map is shown at 2.5 σ above the mean and the reported sharpening B factor is –115 Å^2). The view and slice thickness is the same as in panel A. (**e**) Previously-published USUV map (EMDB: 23273) and USUV model (PDB: 7lch) with M-H1 helices of chains F and D of the model shown. Glu33, Ile37, and Pro40 of both chains are shown and labelled. The map is shown at 3 σ above the mean and the reported sharpening B factor is –70 Å^2). The view and slice thickness is the same as in panel A.
