# Supplementary material for: Molecular Organisation of Tick-Borne Encephalitis Virus
Source: Viruses. 2022 Apr 11;14(4):792. doi: 10.3390/v14040792 (PMC9027435; doi:10.3390/v14040792)
Supplement: Supplementary file 1 [file viruses-14-00792-s001.zip › Table_S1.pdf]

Table S1. Cryo-EM data collection, reconstruction, and modelling statistics

**Data collection and processing**		
	**Preparation 1**	**Final**
Microscope	Thermo Fisher Titan Krios	Thermo Fisher Titan Krios
Detector	Gatan K2	Gatan K3
Magnification	165 000	81 000
Voltage (kV)	300	300
Electron exposure (e^- / Å^2)	28.3	40
Defocus (µm)	-0.9 – -3.0	-0.7 – -2.7
Defocus increment (µm)	0.3	0.2
Pixel size (Å)	0.82	1.08
Symmetry imposed	I2	I2
Initial particle images (no.)	229 504	763613
Final particle images (no.)	15 150	119 210
Map resolution (Å)	3.5	3.3
FSC threshold	0.143	0.143
Map resolution range (Å)	3.2 – 999	3.0 – 999
**Refinement**		
Map sharpening B factor (Å^2)	-5	-5 / variable (see text)

Model composition		
Non-hydrogen atoms	n/a	13194
Protein residues	n/a	1703
Ligands	n/a	9
R.m.s deviations		
Bond lengths (Å)	n/a	0.29
Bond angles (°)	n/a	0.54
Validation		
MolProbity score	n/a	1.28
Clashscore	n/a	4
Ramachandran plot		
Favored (%)	n/a	98
Allowed (%)	n/a	2
Disallowed (%)	n/a	0
