# Supplementary material for: Molecular Organisation of Tick-Borne Encephalitis Virus
Source: Viruses. 2022 Apr 11;14(4):792. doi: 10.3390/v14040792 (PMC9027435; doi:10.3390/v14040792)
Supplement: Supplementary file 1 [file viruses-14-00792-s001.zip › Table_S2.pdf]

Table S2. Comparative structural analysis of the distances between the M protein membrane-proximal helices (H1) of the two M proteins at the heterotetramer interface.

Virus	PDB	H1-H1 distance at the heterotetramer interface (Å)	Reference
**Tick-borne encephalitis (Kuutsalo-14)**	7Z51	19.1	-
**Chimeric flavivirus between Binjari and Murray Valley Encephalitis**	7KVB	29.2	[17]
**Chimeric flavivirus between Binjari and West Nile (Kunjin)**	7KV9	30.9	[17]
**Chimeric flavivirus between Binjari and Dengue serotype-2**	7KV8	32.9	[17]
**Dengue virus serotype-2**	6ZQU	32.7	[19]
**Dengue virus serotype-2**	7V3F	28.9	[64]
**Dengue virus serotype-2**	3J27	31.6	[65]
**Spondweni**	6ZQV	32.1	[19]
**Tick-borne encephalitis (Hypr)**	5O6A	25.7	[4]
**Usutu**	7LCH	36.7	[18]
**Usutu**	7LCG	36.6	[18]
**West Nile virus (Kunjin)**	7KVA	38.6	[17]
**Zika**	6CO8	35.2	[46]
**Zika**	5IZ7	28.6	[66]
**Zika**	5IRE	35.3	[67]
