# Supplementary figures and images for: Molecular Organisation of Tick-Borne Encephalitis Virus
Source: Viruses. 2022 Apr 11;14(4):792. doi: 10.3390/v14040792 (PMC9027435; doi:10.3390/v14040792)

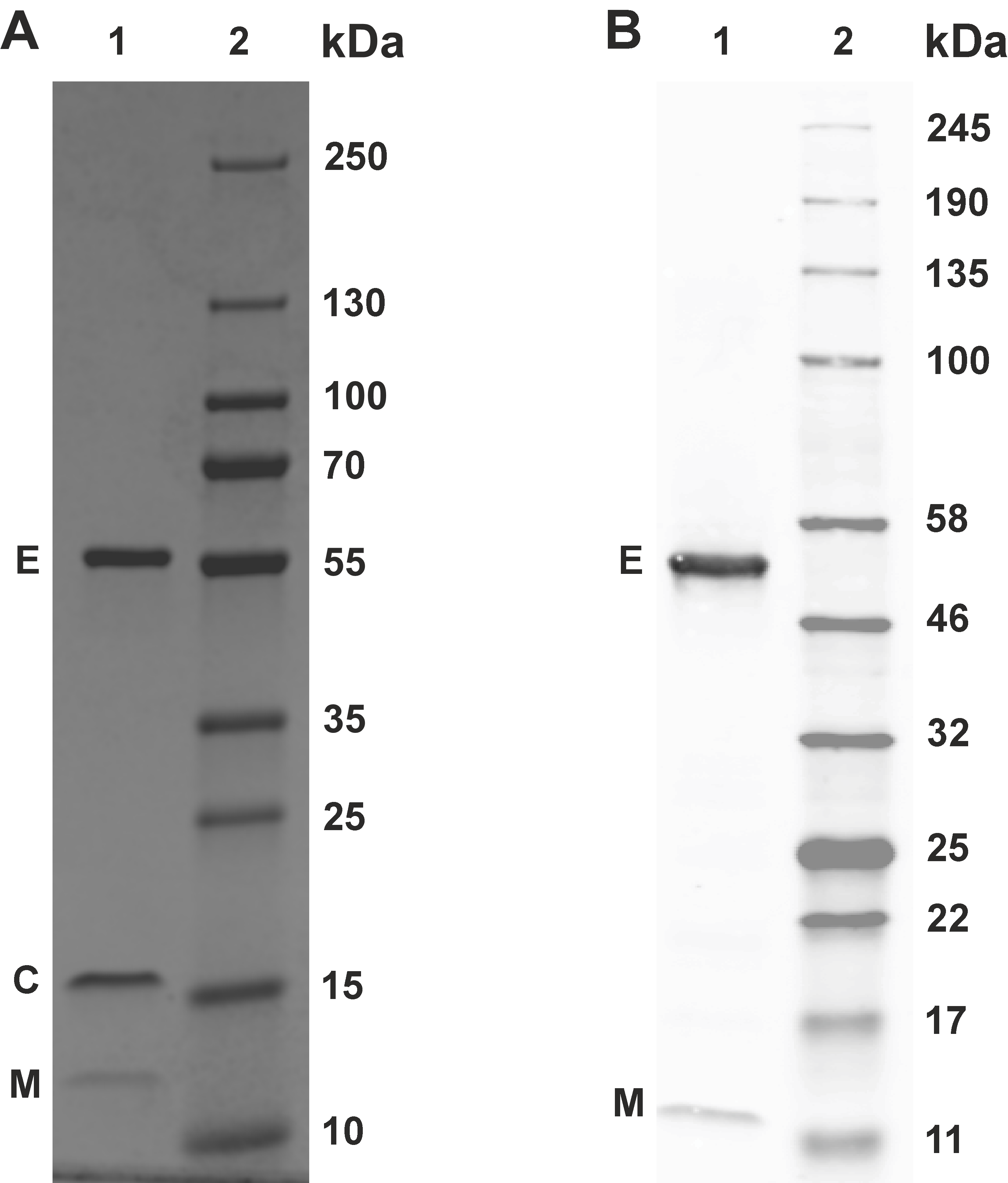

Supplement: Supplementary file 1 [file viruses-14-00792-s001.zip › Figure_S1.tif]

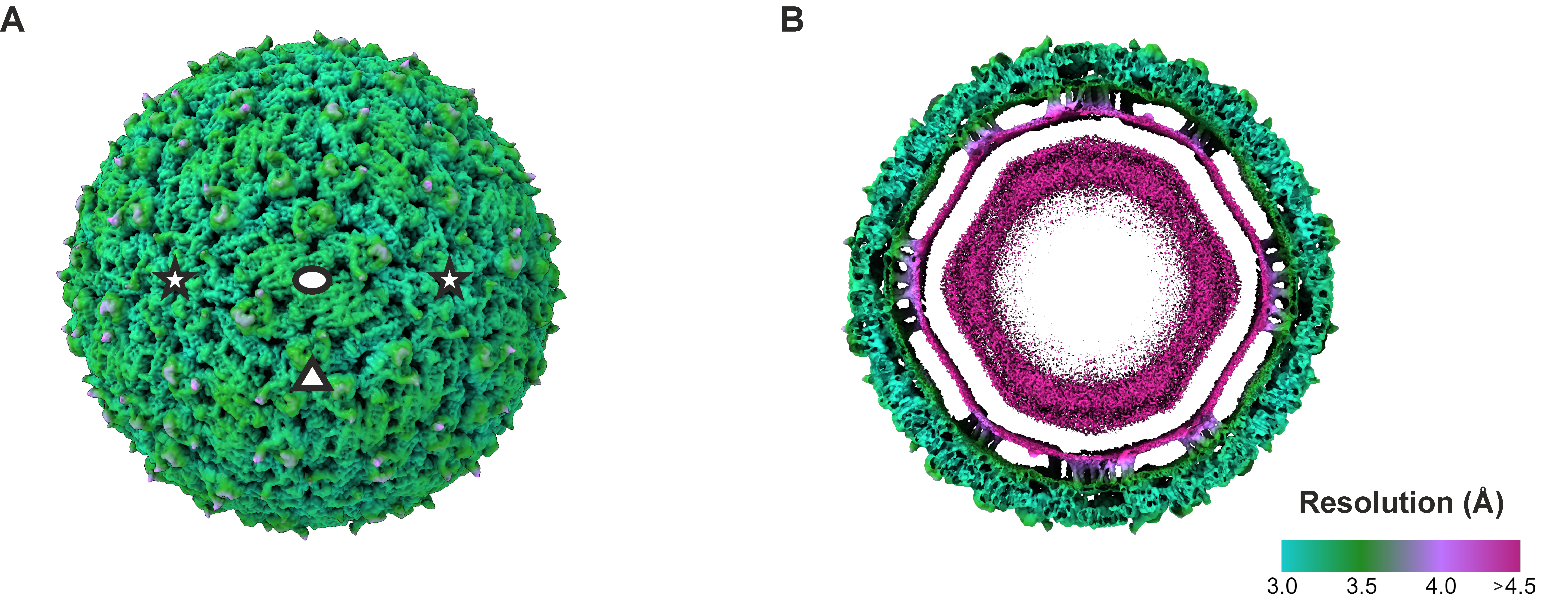

Supplement: Supplementary file 1 [file viruses-14-00792-s001.zip › Figure_S3.tif]

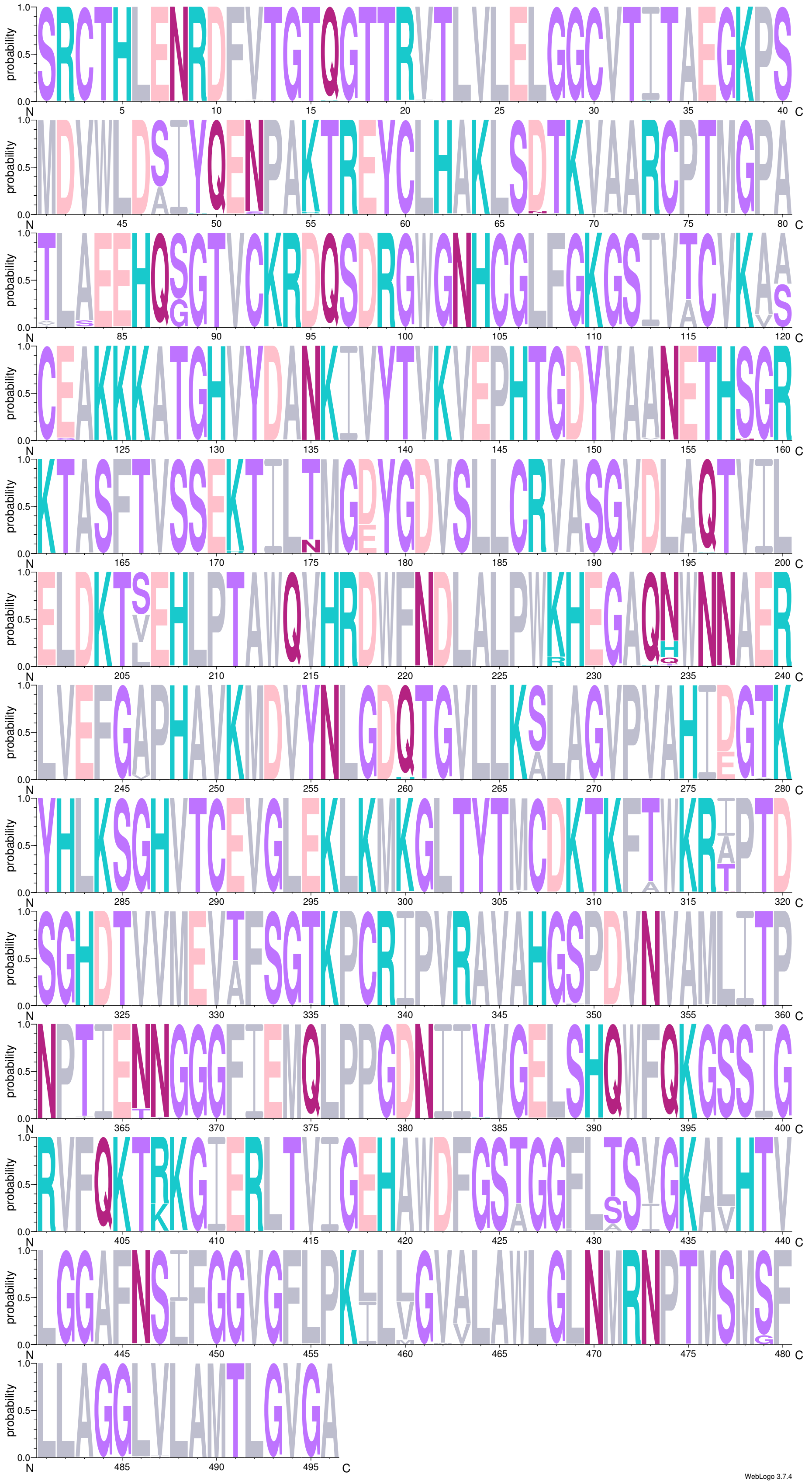

Supplement: Supplementary file 1 [file viruses-14-00792-s001.zip › File_S7_E-Protein-logo.pdf]
