# Supplementary material for: Molecular Organisation of Tick-Borne Encephalitis Virus
Source: Viruses. 2022 Apr 11;14(4):792. doi: 10.3390/v14040792 (PMC9027435; doi:10.3390/v14040792)
Supplement: Supplementary file 1 [file File_S5_M-Protein-logo.pdf › File_S5_M-Protein-logo.pdf]

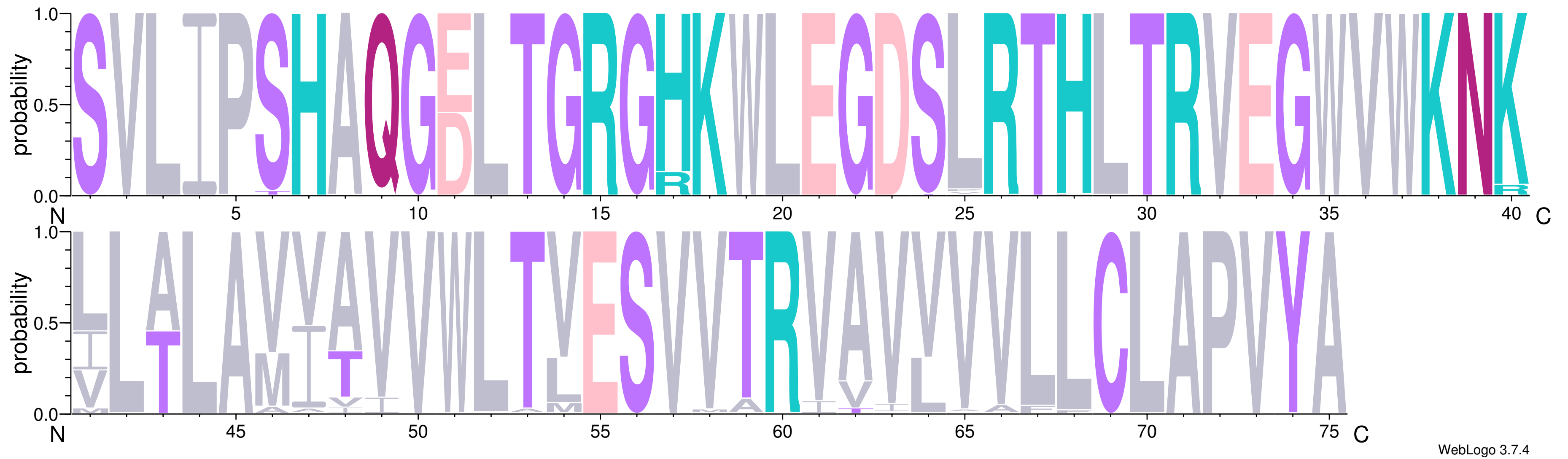

probability
1.0
0.5
0.0
N
5
10
15
20
25
30
35
40
C
45
50
55
60
65
70
75
WebLogo 3.7.4
